# Early Radial Extracorporeal Shockwave Stimulation on Proximal Tibial Circular Osteotomy Site Enhanced Heterotopic Skin Wound Healing via Small Extracellular Vesicles

**DOI:** 10.1002/advs.202517257

**Published:** 2026-01-08

**Authors:** Xiaoping Xie, Songqi Bi, Bang Su, Rongqi Zhou, Xudong Li, Zhixing Yan, Xiaoyang Zhou, Fanxu Wang, Tiecheng Yu

**Affiliations:** ^1^ Department of Orthopedics Orthopedics Center The First Hospital of Jilin University Jilin University Changchun China; ^2^ Department of Hand and Podiatric Surgery Orthopedics Center The First Hospital of Jilin University Jilin University Changchun China

**Keywords:** macrophage polarization, shockwave, small extracellular vesicles, thrombospondin 1, wound healing

## Abstract

Wound healing represents a complex biological process necessitating innovative therapeutic approaches, particularly for chronic wounds such as diabetic foot ulcers (DFUs). Inspired by the regenerative effects of tibial bone transport for DFUs, we explored whether radial extracorporeal shockwave therapy (rESWT) at the tibial annular osteotomy site (TOE) could enhance wound healing in rats. We found that TOE accelerated dorsal foot wound healing via small extracellular vesicles (sEV). This sEVs showed strong regenerative effects and modulated macrophage polarizations in vitro and in vivo. Single‐cell sequencing revealed mesenchymal stem cells as the likely sEV source, with rESWT triggering sEV release through ATP/P2X7R/p38MAPK pathway. Proteomic analysis identified five differentially expressed proteins in sEVs, with Thrombospondin 1 (Thbs1)‐enhanced sEVs enhancing wound healing by promoting IL4‐induced M2 macrophage polarization. This innovative approach represents a promising alternative strategy for wound management, with potential for future DFU treatment.

## Graphical abstract

A schematic diagram of the mechanism where small extracellular vesicles mediate the enhancement of heterotopic wound healing by radial extracorporeal shockwave stimulation at a proximal tibial osteotomy site.

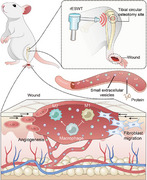

**Abbreviations**
BMDMs: bone marrow‐derived macrophages
BMSCs: bone marrow‐derived mesenchymal stem cells
BT: bone transport
Cdc42: cell division cycle 42
Col1: Collagen I
Col3: Collagen III
DFUs: diabetic foot ulcers
ESWT: extracorporeal shockwave therapy
GO: Gene Ontology
Hspd1: Heat shock protein family D (Hsp60) member 1
ILK: Integrin‐linked kinase
KEGG: Kyoto Encyclopedia of Genes and Genomes) pathway
KOG: Clusters of Orthologous Groups
Lcp1: Lymphocyte cytosolic protein 1
MS: mass spectrometry
MSCs: mesenchymal cell
rESWT: radial extracorporeal shockwave treatment
scRNA‐seq: single‐cell RNA sequencing
sEV^OE‐Thbs1^
: Thbs1‐overexpresing engineered sEVs
sEV^OE‐Vector^
: Vector‐engineered sEVs
sEVs: small extracellular vesicles
Shockwave‐untreated control sEV: (BMSC‐sEVs)
SW: shockwave
SW‐BMSC‐sEVs: shock wave‐treated‐BMSC‐derived sEV
Thbs1: Thrombospondin‐1
TO: tibial osteotomy
TOE: tibial osteotomy site subjected to extracorporeal shockwave stimulation
TTT: tibial transverse transport
VEGFA: vascular endothelial growth factor A

## 1 Introduction

Skin wounds, whether acute or chronic, exhibit diverse etiologies, resulting from external injuries such as trauma, burns, or pressure, as well as internal pathological factors like metabolic disorders or vascular lesions. Large‐area and chronic wounds posed significant clinical challenges due to their susceptibility to infections, impaired healing, and persistent inflammatory responses. The increasing prevalence of diabetes, obesity, and aging populations has further exacerbated the socioeconomic burden of these wounds [1, 2, 3]. Chronic and extensive skin wounds not only compromise the functional and structural integrity of the skin but, if inadequately managed, can lead to limb disability, severe pain, or even mortality. For instance, the mortality rate of chronic diabetic foot ulcers (DFUs) could reach 30.5%, comparable to the 5‐year mortality rate of certain cancers (31%) [4].

Current clinical strategies for wound management generally encompassed surgical debridement, graft transplantation, wound dressings, and physical therapies such as hyperbaric oxygen therapy. However, these approaches are often limited by prolonged healing times, high costs, risks of immune rejection, and susceptibility to infections, which hindered their broad clinical application [5, 6]. The wound repair process involved multiple complex physiological mechanisms, and adverse factors such as chronic inflammation, bacterial infections, and vascular dysfunction could significantly impede recovery, profoundly impacting patients' quality of life. Consequently, there is an urgent need for innovative and effective therapeutic strategies to enhance wound healing, particularly for chronic wounds.

To address these challenges, mechanically driven interventions have demonstrated significant potential in promoting tissue repair. The Ilizarov bone transport (BT) technique, originally developed to manage complex orthopedic disorders, leveraged the tension‐stress principle to stimulate and amplify the regenerative capacity of living tissues, thereby simultaneously promoting the growth and repair of bone, muscle, fascia, vasculature, and nerves [7]. Extensive clinical and preclinical studies had revealed that BT not only enhanced osseous regeneration but also significantly improved the repair of adjacent and even distant soft tissues [8]. A notable derivative, the tibial transverse transport (TTT) technique, has emerged as a highly effective approach for managing DFUs [9, 10], with limb salvage rates up to 90% and one‐year recurrence rates less than 10% in severe cases [11]. A recent large‐scale multicenter cohort study involving 1175 DFU patients further supported TTT as an effective intervention for refractory DFUs [12]. Despite its efficacy, TTT was associated with risks, including fractures, pin‐site infections, and other complications [13]. Consequently, less invasive methods to induce localized tensile stress in bone flaps have emerged as a promising alternative strategy.

Extracorporeal shockwave therapy (ESWT), which employed rapid, short‐duration acoustic waves capable of delivering mechanical energy to biological tissues, represented a unique form of non‐invasive mechanobiological intervention. ESWT promoted tissue repair through mechanotransduction pathways, demonstrating therapeutic benefits in tissue regeneration, wound healing, angiogenesis, bone remodeling, and inflammation modulation [14, 15]. Clinically, low‐intensity ESWT has been shown to be effective in enhancing chronic wound healing, particularly in DFUs and venous ulcers [14, 15], and improving flap survival in acute and chronic soft tissue wounds [16, 17, 18]. In DFUs, emerging evidence indicated that ESWT‐treated patients experienced significantly shorter healing times compared to those receiving standard care [19], a finding supported by preclinical studies demonstrating accelerated wound healing in diabetic animal models [20, 21, 22]. These therapeutic mechanisms appeared multifactorial, involving pro‐angiogenic effects that enhanced perfusion and cellular proliferation [23], suppression of pro‐inflammatory mediators [24], and potential modulation of macrophage behavior to initiate healing [25]. However, its clinical application for plantar ulcers, particularly those with irregular topography in digital regions, is hindered by technical limitations arising from the physical constraints of conventional transducer sizes.

Shockwaves (SW), as transient high‐energy mechanical stimuli, could elicit tensile stress effects at the cellular level [26, 27, 28]. The substantial acoustic impedance difference between osseous and soft tissues led to preferential energy absorption by bone [29], enhancing the biomechanical impact. Inspired by the biomechanical principles of TTT, we proposed replacing TTT's chronic distraction with rapid shockwave stimulation of a tibial osteotomy bone flap to enhance the healing of remote wounds through mechanobiological signaling pathways. In this study, we investigated whether shockwave stimulation of the tibial osteotomy site (TOE) could promote the healing of dorsal foot wounds, and explored whether this process was mediated by the generation of specific small extracellular vesicles (sEVs) that drove wound repair processes. By elucidating the underlying mechanisms, this study offers a novel and clinically viable alternative strategy for wound management.

## 2 Methods

### 2.1 Animals

All animal experimental procedures were approved by the Ethical Committee of the First Hospital of Jilin University (Approval No. JDYY20250651). Male Sprague‐Dawley (SD) rats, aged 6–8 weeks (180–220 g) or 3–4 weeks (100–150 g) were procured from the Jilin Qianhe Model Biotechnology Co., Ltd. (Changchun, China) and were housed under specific pathogen‐free (SPF) conditions. All surgical procedures were conducted under isoflurane (RWD, China) anesthesia delivered in air at a flow rate of 1.0 L/min. Anesthetic depth was monitored by the absence of pedal reflex and stable respiratory rate.

### 2.2 Cell Culture

Bone marrow‐derived mesenchymal stem cells (BMSCs) were isolated from the bone marrow of 3‐week‐old SD rats following previous established protocols [30] and were cultured in DMEM/F12 medium (Gibco, New York, USA) supplemented with 10% exosome‐free fetal bovine serum (FBS; Cellbox, Changsha, China) and 1% penicillin‐streptomycin (P/S, 10 000 U/mL‐10 000 µg/mL, Gibco). Human umbilical vein endothelial cells (HUVEC‐C, Cat#GNHu23, RRID: CVCL_2959), immortalized human keratinocytes cells (HaCaT, Cat# SCSP‐5091, CVCL_0038), and HEK 293T cells (Cat#SCSP‐502, RRID:CVCL_0063) were purchased from the Cell Bank of the Committee on Type Culture Collection, Chinese Academy of Sciences (Shanghai, China) and were maintained in DMEM (Gibco) supplemented with 10% exosome‐free FBS. Human skin fibroblasts (HSFs, Cat#CC‐Y1274, RRID: CVCL_6886) and MC3T3E1 osteoblast cells (Cat#CC‐Y2134, RRID:CVCL_0409) were purchased from the Ek‐Bioscience (Shanghai, China) and HSFs were cultured in DMEM/F12 containing 20% exosome‐free FBS. Bone marrow‐derived macrophages (BMDMs) were isolated by flushing bone marrow from rat femurs and tibias, followed by differentiation in RPMI‐1640 medium (Gibco) supplemented with 10% FBS and 30% L929 cell‐conditioned medium (as a source of macrophage colony‐stimulating factor, M‐CSF) for 7 days. The culture medium was refreshed every 2 days to obtain mature macrophages. All cell cultures exhibited robust growth throughout the cultivation process, with clear culture media and no signs of contamination. Mycoplasma was not detected via PCR analysis of the supernatant. No experiments involving pathogens were conducted in the cell culture facility, and all procedures were performed aseptically with strict measures to prevent cross‐contamination.

### 2.3 In Vitro rESWT for Cultured Cells

To investigate the optimal parameters for radial extracorporeal shockwave treatment (rESWT), BMSCs were isolated to assess the effects of rESWT on cellular viability. Cells from passages 3 to 6 (P3–P6) were used for all experiments. Following enzymatic dissociation, cells were quantified and resuspended in complete DMEM/F12 medium (supplemented with 10% FBS and 1% P/S) at a density of 5 × 10^4^ cells/mL. Aliquots of 1 mL cell suspension were dispensed into sterile 1.5 mL microcentrifuge tubes for shockwave treatment. A radial shockwave generator (CTLNHA, Q80, Shenzhen, China) equipped with a 10 mm focal zone transducer was used to deliver shockwaves at a fixed frequency of 5 Hz. Treatment parameters were systematically varied, with four pressure levels (1, 2, 3, and 4 bar) in combination with four impulse counts (300, 600, 900, and 1200 impulses) being tested [38]. The transducer was coupled to the tube using sterile ultrasound gel. Control samples underwent identical handling without shockwave exposure. All treatments were performed at room temperature.

To elucidate the mechanism underlying shockwave‐induced secretion of sEVs by BMSCs, apyrase (15 U/mL, MCE, USA) was introduced to BMSC cultures prior to shockwave stimulation, while BzATP (a P2X7 receptor agonist, 1 µM, Solarbio, Beijing, China), A‐740003 (a P2X7R inhibitor, 50 nM, Selleck, USA), and SB 203580 (a p38MAPK inhibitor, 10 µM, Selleck) were added 2 h post‐stimulation to assess their roles in the signaling pathway.

### 2.4 Surgical Protocol for Bone Chip via Circular Osteotomy

Male SD rats, aged 6–8 weeks and weighing 200 ± 20 g, were used for all experiments. Under isoflurane anesthesia, the proximal tibia was exposed through a 1.5 cm longitudinal anterior incision. A 3 mm diameter trephine drill was utilized to create a cortical bone defect, centered 3–5 mm distal to the medial tibial plateau. Continuous saline irrigation was applied during drilling to minimize thermal injury. Drilling was ceased after penetrating approximately 2 mm beyond the cortical layer. The cortical bone segment was then carefully mobilized using a 0.8 mm Kirschner wire to form a circular bone chip (3 mm in diameter). The surgical incision was closed in layers using interrupted sutures.

### 2.5 Rat Wound Models

Following preparation of the proximal tibial bone flap, a 4 mm full‐thickness skin defect was surgically created on the dorsal aspect of the hindpaw using a sharp circular dermatome. The wound was subsequently dressed with sterile gauze. Macroscopic evaluation of the skin defects (*n* = 6 per group) was conducted via digital photography on days 1, 3, 5, 7, 10, and 12 post‐injury. Wound boundaries were delineated, and wound areas were quantified using the ImageJ software (National Institutes of Health, USA). Relative wound closure was calculated by normalizing the measured wound areas to baseline values (day 1).

### 2.6 rESWT Transcutaneously on Cortical Bone Chips and GW4869 Administration

Under brief isoflurane anesthesia, rESWT (3 bar, 300 impulses, 5 Hz) were applied transcutaneously to the osteotomy site on days 2 and 4 post‐injury using a 10 mm focal zone transducer. GW4869 (Selleck), an exosome secretion inhibitor, was initially prepared as a stock solution at 8 mg/mL in dimethyl sulfoxide (DMSO). For intravenous administration, the stock solution was diluted in 0.9% normal saline to yield a working solution of 0.3 mg/mL, corresponding to a dose of 2.5 mg/kg body weight. Control animals received 0.9% normal saline containing 3.75% DMSO (v/v) as the vehicle.

### 2.7 sEV Isolation

On day 5 post‐injury, whole blood (8 mL) was collected via cardiac puncture into EDTA‐coated tubes and was stored briefly at 4°C. Samples were centrifuged at 1500 × g for 10 min at 4°C, and the supernatant plasma was carefully aspirated and transferred to fresh tubes. The plasma was further centrifuged at 2000 × g for 10 min at 4°C, then was aliquoted into centrifuge tubes and stored at −80°C for long‐term preservation until subsequent analysis. For sEV isolation, plasma samples and cell culture supernatant were processed as follows: Plasma was diluted 1:1 with sterile phosphate‐buffered saline (PBS), while cell culture supernatant was collected from cells grown in exosome‐depleted FBS medium. Both sample types were then centrifuged at 2000 × g for 30 min at 4°C to remove cellular debris, and the supernatants were transferred to ultracentrifuge tubes. The supernatants underwent sequential ultracentrifugation: that were centrifuged at 10 000 × g for 30 min at 4°C to remove large extracellular vesicles, followed by centrifugation at120 000 × g for 2 h at 4°C to pellet the sEV fraction. The sEV pellets were resuspended in 1 mL PBS, combined if necessary, and were centrifuged again at 120 000 × g for 1 h at 4°C for washing. Finally, purified sEVs were resuspended in 200 µL PBS and stored at −80°C.

### 2.8 sEV Characterization

For morphological characterization, 10 µL of the sEV suspension was applied to a copper grid and was incubated for 1 min at room temperature to allow adsorption. Negative staining was performed by applying 10 µL of 20 mM phosphotungstic acid (pH 7.0, Solarbio) to the sample side for 1 min. The grids were air‐dried and were examined using a transmission electron microscope (TEM, Hitachi HT‐7800, Japan) operated at 80 kV.

The size distribution and particle concentration of sEVs were analyzed using a NanoSight NS300 system (Malvern Panalytical, UK). sEV samples were diluted in sterile PBS to an optimal concentration (2–8 × 10^8^ particles/mL) and were loaded into the chamber. Five 60 s videos were recorded for each sample under standardized settings. Data were processed using NTA 3.4 software to determine particle size distribution and concentration.

For sEV‐specific protein markers, positive (CD63, CD9, TSG101) and negative (Calnexin) markers (Proteintech, Wuhan, China) were detected via Western blot. sEVs were lysed in RIPA buffer with protease inhibitors, and protein concentrations were measured using a BCA assay (Beyotime, Shanghai, China).

### 2.9 Fluorescent Labeling of sEVs

sEVs were fluorescently labeled using either the PKH26 Kit or DiI kit (Solarbio, Beijing) with slight modifications to the manufacturers’ protocols. For PKH26 labeling, sEVs (100 µg/mL in PBS) were suspended in 0.5 mL Diluent C and were incubated with 2 µL PKH26 dye for 4 min at room temperature. For DiI labeling, sEVs (100 µg/mL in PBS) were suspended in 0.5 mL sterile PBS and were incubated with 2 µL DiI (1 mM stock in DMSO) for 10 min at 37°C. Both labeling reactions were quenched by adding 2 mL of 1% bovine serum albumin (BSA) in PBS. To remove unincorporated dye, labeled sEVs were purified by ultracentrifugation at 120 000 × g for 70 min at 4°C. The purified, labeled sEVs were resuspended in sterile PBS for subsequent experiments.

### 2.10 Cellular Uptake Assay

For cellular uptake studies, recipient cells were incubated with PKH26‐labeled sEVs for 12 or 24 h at 37°C in a 5% CO_2_ atmosphere. Post‐incubation, cells were fixed with 4% paraformaldehyde, permeabilized with 0.1% Triton X‐100, and stained with phalloidin (1:200, Beyotime, Shanghai,China) to visualize F‐actin and DAPI (1 µg/mL, Solarbio, Beijing) for nuclear counterstaining. Internalization of sEVs was evaluated using confocal laser scanning microscopy (Nikon AXR, Japan).

### 2.11 Histological Staining

Regenerative skin tissues from rats were harvested on days 5 and 12 post‐injury. Tissues were fixed in 4% paraformaldehyde for 48 h, dehydrated, embedded in paraffin, and sectioned into 4 µm slices. Hematoxylin and eosin (H&E) staining and Masson's staining were performed on the tissue sections according to the manufacturers’ protocols (Solarbio, Beijing). The stained slides were imaged using a VS200 Whole‐Slide Scanning System (Olympus, Tokyo, Japan).

### 2.12 Immunofluorescence Staining and Immunohistochemistry

Paraffin‐embedded tissue sections (4 µm) from regenerative skin tissue were deparaffinized, rehydrated, and subjected to antigen retrieval in Tris‐EDTA buffer (pH 9.0) at 95°C for 20 min. After cooling to room temperature, sections were incubated with a peroxidase blocking solution for 10 min, permeabilized with 0.1% Triton X‐100 in PBS for 10 min, and then blocked with 5% BSA in PBS for 1 h at room temperature. Primary antibodies (Col1, Col3, 1:1000; CD31,1:5000, Proteintech. CD68, iNOS, Arg1, 1:200, Servicebio, Wuhan, China) were applied and incubated overnight at 4°C. Subsequently, for immunofluorescence, sections were incubated with fluorophore‐conjugated secondary antibodies (1:1000, Proteintech) for 1 h, nuclei were counterstained with DAPI for 5 min. For immunohistochemistry, sections were incubated with HRP‐conjugated secondary antibodies (1:500, Proteintech) for 1 h, followed by detection with a DAB substrate for 1–5 min. The stained slides were imaged using a VS200 Whole‐Slide Scanning System (Olympus). Three independent experiments were conducted.

### 2.13 Immunofluorescence Staining of Cells

Cells were fixed with 4% paraformaldehyde for 10 min, permeabilized with 0.1% Triton X‐100 for 10 min, and blocked with 5% BSA in PBS for 1 h. Primary antibodies (CD68, iNOS, Arg1, 1:200, Servicebio) were applied and incubated overnight at 4°C. Subsequently, samples were incubated with fluorophore‐conjugated secondary antibodies (1:1000, Proteintech) for 1 h at room temperature. Nuclei were counterstained with DAPI for 5 min. Fluorescence images were acquired using confocal laser scanning microscopy (Nikon). Three independent experiments were conducted.

### 2.14 Western Blot

Tissues and cells were lysed in RIPA buffer supplemented with protease and phosphatase inhibitors, and protein concentrations were measured using a BCA assay (Beyotime). Proteins (10–20 µg) were separated on 10% SDS‐polyacrylamide gels (Coolaber, China) and were transferred to PVDF membranes (Millipore, Merck, Germany). Membranes were blocked with 5%BSA in TBST for 1 h at room temperature, followed by incubation with primary antibodies (iNOS, Arg1, VEGFA, Col1a1, Thbs1, Lcp1, ILK, HSPD1, Cdc42, GAPDH, β‐actin, α‐Tubulin all 1:1000, Proteintech; Col3a1,1:1000; P38MAPK, p‐P38MAPK, PI3K, p‐PI3K, Akt, p‐Akt, P2X7R, all 1:1000, CST; CD206, 1:1000, HuaBio; Flag, 1:1000, aBclonal) overnight at 4°C. After washing with TBST, membranes were incubated with HRP‐conjugated secondary antibodies (1:10 000, Proteintech) for 1 h at room temperature. Bands were visualized using ECL substrate and imaged with a chemiluminescence detection system. Band intensities were quantified using ImageJ software.

### 2.15 Cell Migration

Cell migration was assessed using 8 µm pore Transwell inserts in a 12‐well format (Corning, USA). HUVECs and HSFs (5 × 10^4^ cells/insert) were seeded in medium containing 2% exosome‐free serum in the upper chamber, while the lower chamber contained complete medium with sEVs (80 µg/mL). After a 24‐h incubation at 37°C in a 5% CO_2_ atmosphere, migrated cells on the lower surface of the membrane were fixed with 4% paraformaldehyde for 10 min, stained with 0.1% crystal violet, and quantified by counting cells in three randomly selected fields per insert under an optical microscope.

### 2.16 Wound Healing Assay In Vitro

HUVECs and HaCaTs were seeded in 6‐well plates and cultured until reaching 90%–100% confluency. A linear scratch wound was generated using a 1 mL pipette tip, and the cells were then incubated in medium containing 2% exosome‐free serum supplemented with sEVs at concentrations of 80 µg/mL. After 12 and 24 h of incubation at 37°C, wound closure was observed under an optical microscope, and quantitative analysis was performed by measuring the wound area in each well.

### 2.17 Tube Formation

Tube formation was assessed using HUVECs seeded on Matrigel (Corning, USA)‐coated 15‐well plates (ibidi, Germany). HUVECs (2 × 10^4^ cells/well) were resuspended in medium containing 10% exosome‐free FBS supplemented with sEVs at concentrations of 80 µg/mL. Cells were incubated at 37°C in a 5% CO_2_ atmosphere for 6 h. Tube‐like structures were observed under an optical microscope, and were analyzed using ImageJ software to quantify the number of tubes.

### 2.18 CCK8

Cell viability was assessed using a Cell Counting Kit‐8 (CCK‐8, Coolaber, China) assay. HUVECs (5 × 10^3^ cells/well) were seeded in 96‐well plates in medium containing 10% exosome‐free FBS supplemented with sEVs at concentrations of 40 and 80 µg/mL. After 24 and 48 h of incubation at 37°C in a 5% CO_2_ atmosphere, 10 µL of CCK‐8 reagent was added to each well, and cells were incubated for an additional 1 h. Absorbance was measured at 450 nm using a microplate reader. Cell viability was calculated relative to the control group.

### 2.19 LC‐MS/MS Analysis of sEVs

Proteomic analysis of the TO‐sEV and TOE‐sEV groups (*n* = 3) was performed by Sangon Biotech (Shanghai, China), encompassing sample quality control, preprocessing, mass spectrometry (MS), and reporting. In brief, proteins were extracted from samples using lysis buffer (8 m urea, 1 mm PMSF, 2 mm EDTA), followed by sonication on ice for 5 min and centrifugation at 15 000 × g for 10 min at 4°C. Protein concentrations were quantified using a BCA assay (Beyotime). For digestion, 100 µg of protein was reduced with 5 mm DTT at 37°C for 45 min, was alkylated with 11 mm iodoacetamide at room temperature for 15 min, and was digested overnight with trypsin (Promega, V5280) in 25 mm NH_4_HCO_3_ at 37°C. The resulting peptides were desalted using C18 columns (Millipore, Billerica, MA) and were quantified with the Pierce Quantitative Peptide Assay (Thermo Fisher Scientific).

Peptides were separated on a NanoElute UHPLC system (IonOpticks, Australia, C18 column, 15 cm × 75 µm, 1.6 µm) using a 20‐min gradient (5–95% B; 0.1% formic acid in acetonitrile) at a flow rate of 500 nL/min. Mass spectrometry was performed using a timsTOF Pro2 (Bruker Daltonics) in ddaPASEF and diaPASEF modes. For ddaPASEF, parameters included a 100–1700 m/z scan range, 0.85–1.3 Vs/cm^2^ ion mobility range, 100 ms accumulation time, 1500 V capillary voltage, and collision energy ramping from 27–45 eV with a 0.53 s cycle time and 4 MS/MS scans. For diaPASEF, 48 windows (400–1200 m/z, 12 Th width) were used with a 1.17 s cycle time, maintaining 3 L/min dry gas at 180°C.

DIA data were processed using DIA‐NN (v1.8.1) in library‐free mode with a Uniprot Rat proteome database (47,930 entries), enabling match‐between‐runs and deep learning‐based spectral prediction, with identifications being filtered at a 1% FDR at both precursor and protein levels.

### 2.20 Polarization Induction of BMDMs

Primary BMDM were cultured in DMEM supplemented with 10% FBS, 1% P/S, and 30% L929 fibroblasts‐conditioned medium at 37°C in a 5% CO_2_ atmosphere for 7 days. Macrophages were then harvested and seeded in 12‐well or 6‐well plates. Cells were treated with sEVs for 2 h before exposure to lipopolysaccharide (LPS, 100 ng/mL, Solarbio) or recombinant mouse IL‐4 (20 ng/mL, PeproTech Inc.) for 24 h in DMEM supplemented with 10% exosome‐free FBS, 1% P/S, and 30% L929‐conditioned medium.

### 2.21 Flow Cytometry

Flow cytometry was performed to evaluate the effect of sEVs on lipopolysaccharide (LPS, 100 ng/mL, Solarbio)‐induced M1 and recombinant mouse IL‐4 (20 ng/mL, PeproTech Inc., USA)‐induced M2 polarization of BMDMs. Briefly, BMDMs (1 × 10^6^ cells/mL) were seeded in 6‐well plates and treated with LPS or IL‐4 in medium containing 10% exosome‐free FBS, with or without sEVs (80 µg/mL), for 24 h at 37°C in a 5% CO_2_ atmosphere. Cells were then harvested, washed, and stained with fluorescently labeled antibodies against macrophages (FITC‐CD68, PE‐CD11b, 1:200, BioLegend, USA), and M1 markers (APC‐CD86, 1:200, BioLegend, USA) for 30 min at 4°C in the dark. For CD163 (M2 markers), cells were incubated with unconjugated anti‐CD163 primary antibody (1:200, Abcam, USA) for 30 min at 4°C, washed, and then incubated with Alexa Fluor647‐conjugated secondary antibody (1:500) for 30 min at 4°C in the dark. After final washing with PBS, cells were analyzed using BD FACSymphony A5(BD, USA), with data from 10 000–20 000 events per sample being processed using FlowJo software (v10.8.1) to quantify M1 and M2 populations.

### 2.22 CellTiter‐Lumi Plus Luminescent Assay for Extracellular ATP Concentration in BMSCs

Extracellular ATP concentrations from BMSCs were measured using the CellTiter‐Lumi Plus Luminescent Assay (Beyotime). After enzymatic dissociation, BMSCs (1 × 10^4^ cells/well) were transferred to the Eppendorf tubes and subjected to rESWT stimulation under inverted conditions. The cells were then centrifuged, and the supernatant was immediately collected. Subsequently, 100 µL of each supernatant was transferred to a white opaque 96‐well plate. An equal volume of CellTiter‐Lumi Plus reagent was added to each well, and the plate was incubated at room temperature in the dark for 10 min. Luminescence intensity was measured using a microplate reader, and ATP concentrations were calculated based on a standard curve.

### 2.23 Plasmid Construction and Transfection

The PCMV‐N‐Flag plasmid was linearized using *Bgl II* restriction enzyme (Takara, Japan). cDNAs for Lcp1, Thbs1, Cdc42, ILK, and Hspd1 (Miaoling Biology, Wuhan,China) were amplified by PCR using high‐fidelity DNA polymerase (Takara) and gene‐specific primers with homologous sequences for seamless cloning: Lcp1 (F: 5'‐ATTCGATATCGTCGACAGATCTATGGCCAGAGGATCCGTGTC‐3', R: 5'‐AGTTCTAGACTCGAGAGATCTTTACACCCTCTTCATCCCTTTC‐3'), Thbs1 (F: 5'‐ATTCGATATCGTCGACAGATCTATGGAGCTCCTCAGGGGACT‐3', R: 5'‐AGTTCTAGACTCGAGAGATCTTTAGGAGTCTCGGCACTCGT‐3'), Cdc42 (F: 5'‐ATTCGATATCGTCGACAGATCTATGCAGACAATTAAGTGTGT‐3', R: 5'‐AGTTCTAGACTCGAGAGATCTTCATAGCAGCACACACCTGC‐3'), ILK (F: 5'‐ATTCGATATCGTCGACAGATCTATGGACGACATTTTCACTCA‐3', R: 5'‐AGTTCTAGACTCGAGAGATCTCTACTTGTCCTGCATCTTCT‐3'), Hspd1 (F: 5'‐ATTCGATATCGTCGACAGATCTATGCTTCGACTACCCACAGT‐3', R: 5'‐AGTTCTAGACTCGAGAGATCTTTAGAACATGCCACCTCCCA‐3'). PCR conditions were 98°C for 30 s, 30 cycles of 98°C for 10 s, 60°C for 20 s, 72°C for 30 s/kb, and 72°C for 5 min. Purified PCR products and linearized plasmid were assembled using a seamless cloning kit (In‐Fusion, Beyotime) at 50°C for 15 min, transformed into DH5α *E. coli* (AlpalifeBio, Guangdong, China), and selected on LB agar with 0.1% ampicillin (100 mg/mL, MCE, USA). Recombinant plasmids were verified by restriction digestion and Sanger sequencing. For plasmid transfection in 293T cells, different doses (0, 1, 6, 12, 24 µg) of plasmid DNA were complexed with PEI (1:1.5 ratio, MCE, USA) and transfected into cells at 70–80% confluency, followed by analysis at 24 and 48 h post‐transfection.

### 2.24 Single‐Cell RNA Sequencing Analysis

Single‐cell sequencing (scRNA‐seq) was primarily performed by OE Biotech (Shanghai, China)., encompassing sample lysis, quality control, sequencing, and data analysis. In brief, single‐cell suspensions were prepared from fresh tissue samples, followed by library construction using the 10x Genomics Chromium platform. After quality control (QC) filtering, high‐quality cells were clustered via Seurat (v4.0) with PCA and UMAP dimensionality reduction. Differential gene expression analysis was performed (|log2FC| > 0.25, adjusted *p* < 0.05), and cell types were annotated using marker genes from reference databases. All analyses were conducted in R (v4.1.0).

### 2.25 Statistical Analysis

All data were analyzed using SPSS (version 25.0, IBM, USA). Results were presented as mean ± standard deviation from at least three independent experiments (*n* = 3). Normality of data distribution was assessed using the Shapiro‐Wilk test, and homogeneity of variances was tested using Levene's test. For comparisons between two groups, an unpaired Student's t‐test was used for normally distributed data with equal variances. For multiple group comparisons, one‐way analysis of variance (ANOVA) with Tukey's post‐hoc test was applied for parametric data with equal variances, while Dunnett's T3 test was used for parametric data with unequal variances. A p‐value < 0.05 was considered statistically significant.

## 3 Results

### 3.1 TOE Accelerates Dorsal Foot Skin Wound Healing via Small Extracellular Vesicles

To determine the optimal rESWT parameters, we initially conducted in vitro experiments to evaluate the effects of rESWT on the viability of BMSC and MC3T3E1 osteoblast cells. The parameter set of 3 bar and 300pulses (5 Hz) was identified as optimal, exhibiting minimal cellular disruption while significantly enhancing the viability of BMSC and MC3T3E1 osteoblasts (Figure S1). Based on these in vitro findings indicating a beneficial and safe profile, we employed this candidate parameter set in subsequent animal experiments to investigate its therapeutic potential.

**FIGURE 1:**
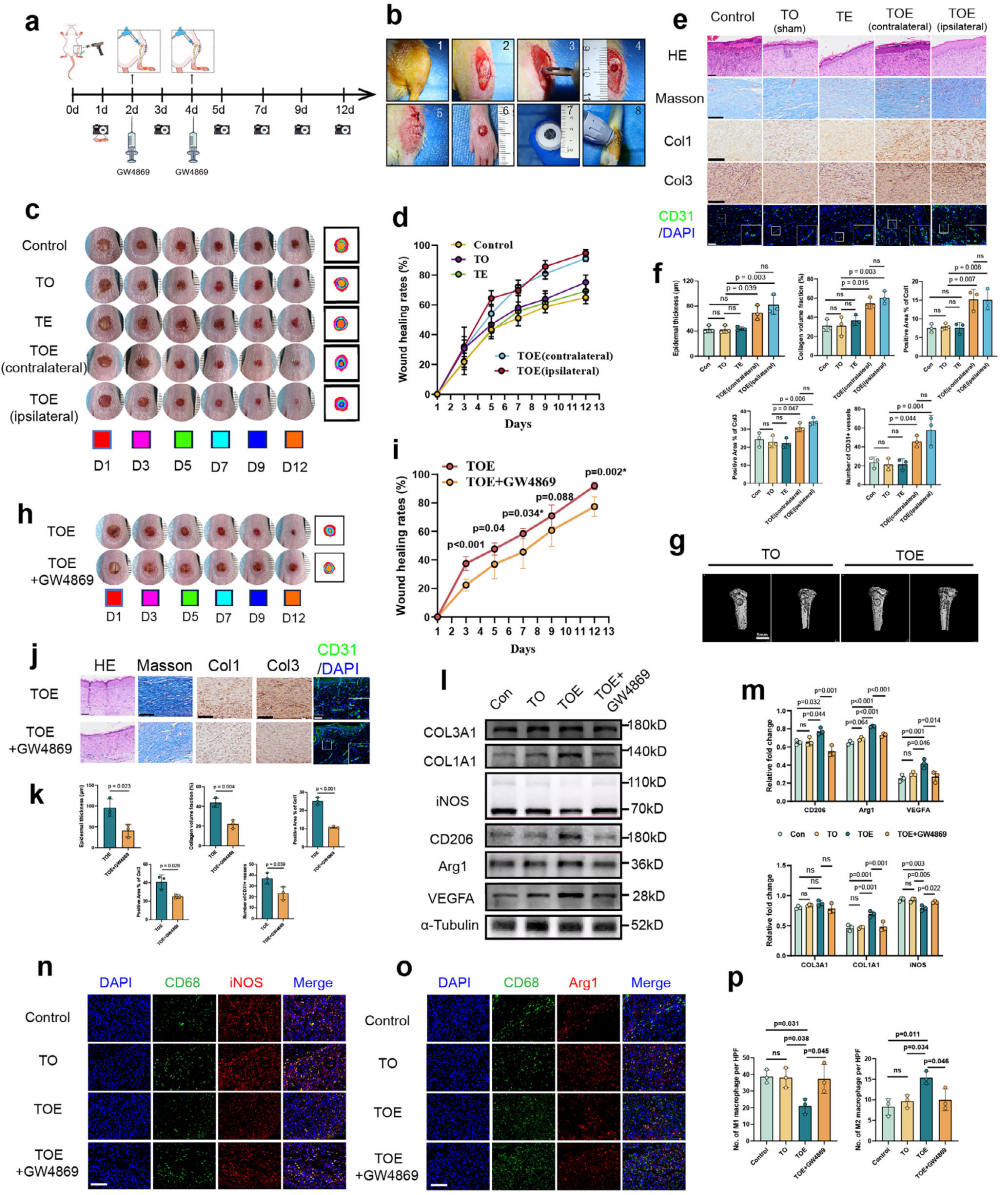
TOE accelerates dorsal foot skin wound healing via small extracellular vesicles. (a) Schematic diagram of the experimental model for accelerated dorsal foot wound healing in rats through shock wave stimulation at the tibial osteotomy site. (b) Surgical procedures for establishing tibial cortical circular osteotomy and dorsal foot full‐thickness wound creation in rat models, along with images of percutaneous rESWT application to tibial osteotomy site (TOE) on day 2 post‐injury. (c) Gross images of rat dorsal foot wounds on days 1, 3, 5, 7, 10, and 12. The control group consisted of a skin wound healing model without any additional interventions. The tibial osteotomy (TO) group underwent tibial osteotomy without subsequent rESWT treatment. The tibial exposure (TE) group underwent tibial exposure without osteotomy but received rESWT treatment. The TOE group received both tibial osteotomy and rESWT, with subsequent monitoring of wound healing progression in ipsilateral or contralateral dorsal foot wounds. (d) Quantitative analysis of wound healing rates across the five groups was performed using ImageJ software. (*n* = 6) (e,f) Representative histological images (H&E and Masson staining) and immunohistochemical/immunofluorescence staining (Col1, Col3, and CD31) are presented for all five groups, with corresponding quantitative analyses are presented for all five experimental groups. (scale bars: 100 µm, *n* = 3). (g) 3D micro‐CT images of tibial osteotomy sites in TO and TOE group at day 12 post‐operation. (h,i) Gross appearance of dorsal foot wounds on days 1, 3, 5, 7, 9, and 12. TOE+GW4869 group underwent TOE combined with GW4869 administration. Quantitative analysis of wound healing rates was performed using ImageJ software. (*n* = 6) (j,k) Representative histological images (H&E and Masson staining) and immunohistochemical/immunofluorescence staining (Col1, Col3, and CD31) are presented for the TOE and TOE+GW4869 groups, with corresponding quantitative analyses (scale bars: 100 µm, *n* = 3). (l,m) Western blot analysis of wound tissues collected on day 5 post‐injury revealed differential expression of COL3A1, COL1A1, iNOS, CD206, Arg1 and VEGFA among the Control, TO, TOE and TOE+GW4869 groups. (*n* = 3) (n–p) Immunofluorescence analysis of wound tissues collected on day 5 post‐injury showed differential expression levels of iNOS (M1 macrophage marker) and CD206 (M2 macrophage marker) among the Control, TO, TOE, and TOE+GW4869 groups. (scale bars: 100 µm, *n* = 3). Data are presented as the mean ± SD. * indicated that these groups were performed using Welch's t‐test due to unequal variances. ns, not significant. *p* < 0.05 was regard as statistically significant.

For this study, forty‐two 8‐week‐old Sprague‐Dawley (SD) rats were utilized to establish animal models (Figure 1a). After establishing tibial circular osteotomy (TO) and creating 4‐mm‐diameter full‐thickness circular dorsal foot skin wounds on day 0, rESWT was applied to the tibial osteotomy site (TOE group) on days 2 and 4 post‐injury, while the TO group did not receive rESWT stimulation (Figure 1b).

After observing statistically significant differences in wound closure rates (TOE vs. TO and control groups, p<0.05) at day 5 post‐injury, subsequent rESWT applications were discontinued (Table S1). Notably, enhanced wound healing rates were observed not only in the ipsilateral but also in the contralateral dorsal foot wounds at days 7, 9, and 12 compared to the TO and control groups (Figure 1c,d; and Table S1). Micro‐CT and histological analyses at days 12 post‐injury revealed enhanced trabecular bone formation both surrounding and within the bone flap in the TOE group, confirming the efficacy and safety of this therapeutic approach (Figure 1g; Figure S2d).

Histopathological analysis revealed significantly increased epidermal thickness, collagen deposition, and neovascularization in the TOE group compared to the TO and control groups. Additionally, expression levels of collagen I (Col1) and collagen III (Col3) were markedly elevated in the TOE group (Figure 1e,f).

These findings suggested that rESWT applied to the osteotomy site might have promoted promoted heterotopic wound healing via circulating factors. Given the potential role of sEVs as key mediators of wound healing, we pharmacologically inhibited sEV secretion using intravenous administration of GW4869 to evaluate their functional contribution. As anticipated, GW4869 administration significantly impaired wound healing progression in the TOE group at days 5, 7, and 12 post‐injury, accompanied by reduced epidermal thickness, collagen deposition, and neovascularization compared to untreated TOE controls (Figure 1h–k).

On days 5 post‐injury, wound tissues from the TOE group exhibited significantly elevated protein expression of the pro‐healing mediators, including vascular endothelial growth factor A (VEGFA), Col1, Col3, and CD206 (a M2 macrophage marker), alongside reduced expression of iNOS (a M1 macrophage marker) compared to the TO group. Conversely, GW4869 administration significantly attenuated expression of VEGFA, Col1, Col3, and CD206 in TOE‐treated wounds, while increasing iNOS expression (Figure 1l,m). Immunofluorescence analysis revealed that the TOE treatment significantly reduced M1 macrophage infiltration while promoting M2 polarization in wound tissues on days 5 post‐injury compared to the TO controls. However, GW4869 administration reversed this effect, resulting in increased M1 macrophage markers and decreased M2 macrophage markers (Figure 1n–p). These results suggested that TOE might accelerate skin wound healing by promoting a shift from M1 to M2 phenotypes in the wound tissue, and that sEV release was required for TOE‐mediated healing enhancement.

We further compared the efficacy of TOE, rESWT alone, and tibial transverse transport (TTT) on wound healing. The results demonstrated that TOE exhibited superior therapeutic efficacy compared to localized rESWT alone at days 5, 7, 9 and 12 post‐injury; however, no significant difference was observed relative to TTT. Moreover, it was found that tibial osteotomy with bone flap preservation upregulated the rates of wound healing following rESWT compared to non‐preserved groups at days 5, 7, 9 and 12 post‐injury (Figure S2a–c; and Table S4).

### 3.2 Plasma‐Derived sEVs from the TOE Group Exhibit Regenerative and Immunomodulatory Activities In Vitro

**FIGURE 2:**
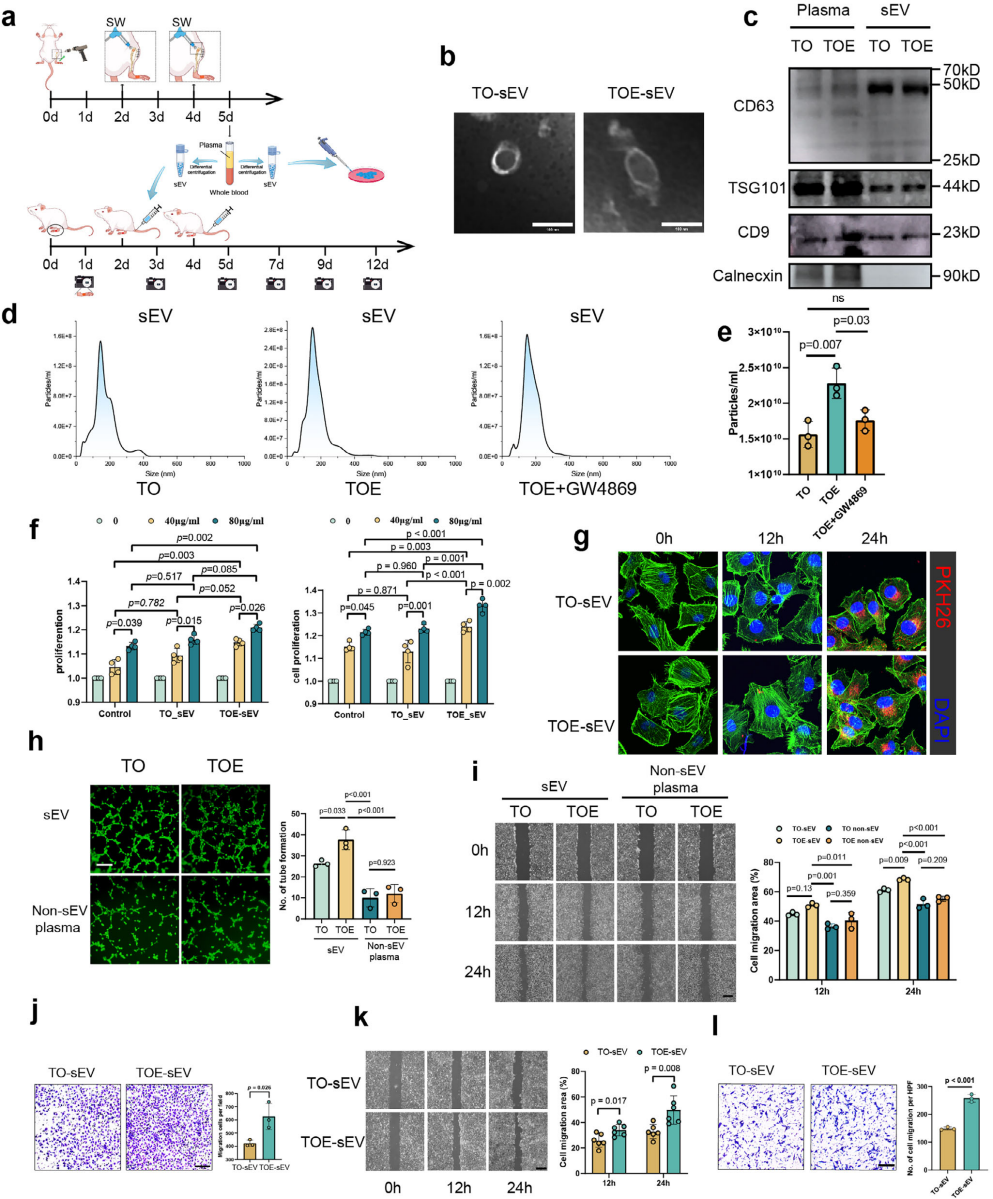
Plasma‐derived sEVs from the TOE group exhibited regenerative activities in vitro. (a) Schematic diagram illustrating the experimental workflow of sEV isolation from day 5 post‐injury whole blood from TO and TOE groups, followed by functional validation in both in vitro and in vivo wound healing models. (b) Transmission electron microscopy (TEM) revealed characteristic cup‐shaped, double‐membrane structures of isolated TO‐sEVs and TOE‐sEVs, confirming their exosomal morphology. (scale bar: 100 nm) (c) Western blot analysis confirmed the presence of sEV markers (CD63, CD9, and TSG101) in plasma‐derived sEVs isolated from the TO and TOE group. (d,e) NTA analysis revealed a monodisperse size distribution of sEVs (peak < 200 nm) across all groups, with TOE‐derived sEVs exhibiting significantly higher particle concentrations compared to both TO and TOE+GW4869 groups. (*n* = 3) (f) CCK‐8 assay revealed the dose‐ and time‐dependent effects of sEVs on HUVEC viability at 24 and 48 h post‐treatment. (*n* = 4) (g) PKH26‐labeled TO‐ and TOE‐derived sEVs were internalized by HUVECs within 12 and 24 h. (scale bar: 50 µm) (h) sEV and non‐sEV component were isolated from the plasma of TO or TOE rats. HUVEC tube formation assay demonstrated the differential angiogenic potential of sEVs and non‐sEV factors form TO and TOE, respectively. (scale bar: 200 µm, *n* = 3) (i) Quantitative analysis of scratch wound closure demonstrated the differential HUVEC migration potential of sEVs and non‐sEV factors form TO and TOE, respectively. (scale bar: 500 µm, *n* = 3) (j) Transwell migration assays revealed that TOE‐sEVs enhanced HUVEC migration compared to TO‐sEVs at 24 h. (scale bar: 500 µm, *n* = 3) (k) Quantitative analysis of scratch wound closure revealed that TOE‐sEVs enhanced Hacat cells migration compared to TO‐sEVs at 12 and 24 h. (scale bar: 500 µm, *n* = 3) (l) Transwell assay assessed the effects of TO‐ and TOE‐derived sEVs on HSF migration at 24 h. (scale bar: 200 µm, *n* = 3). Data are presented as the mean ± SD. ns, not significant. *p* < 0.05 was regard as statistically significant.

To further validate the therapeutic effects of plasma‐derived sEVs on skin wound healing in vitro, we established tibial circular osteotomy models with rESWT (TOE group) or without rESWT (TO group). Plasma sEVs were isolated from the whole blood via differential ultracentrifugation at 24 h after the second rESWT (day 5 post‐injury, Figure 2a). Purified sEVs from both TOE and TO groups exhibited typical cup‐shaped, bilayered membrane morphology under the TEM analysis and expressed exosomal markers (CD63, CD9, and TSG101) while being negative for Calnexin. Nanoparticle tracking analysis (NTA) revealed a predominantly unimodal size distribution with peak diameters within the 200 nm range. Notably, TOE‐derived sEVs exhibited significantly higher particle concentrations compared to TO‐derived sEVs (Figure 2b–e).

Subsequent in vitro experiments demonstrated that TOE‐sEVs significantly promoted HUVEC proliferation in a concentration‐dependent manner (Figure 2f). Fluorescence tracking of PKH26‐labeled sEVs exhibited time‐dependent cellular uptake patterns for both TO‐ and TOE‐derived sEVs, with comparable internalization efficiencies observed across groups at each time point (Figure 2g). Furthermore, to investigate whether other plasma components mediated the promotion of ectopic wound healing by TOE, we separated TOE plasma into purified sEVs and non‐sEV plasma. As anticipated, at a protein concentration of 80 µg/ml, purified sEVs from TOE group exhibited a markedly enhanced capacity to promote HUVEC tube formation and migration in vitro than the non‐sEV plasma (Figure 2h,i). Moreover, at a concentration of 80 µg/mL, TOE‐sEVs significantly enhanced the migration of HUVECs, HaCaTs, and HSFs, while also promoting HUVEC tube formation compared to TO‐sEVs, indicative of their pro‐angiogenic and pro‐migratory potential for wound healing (Figure 2h–l).

**FIGURE 3:**
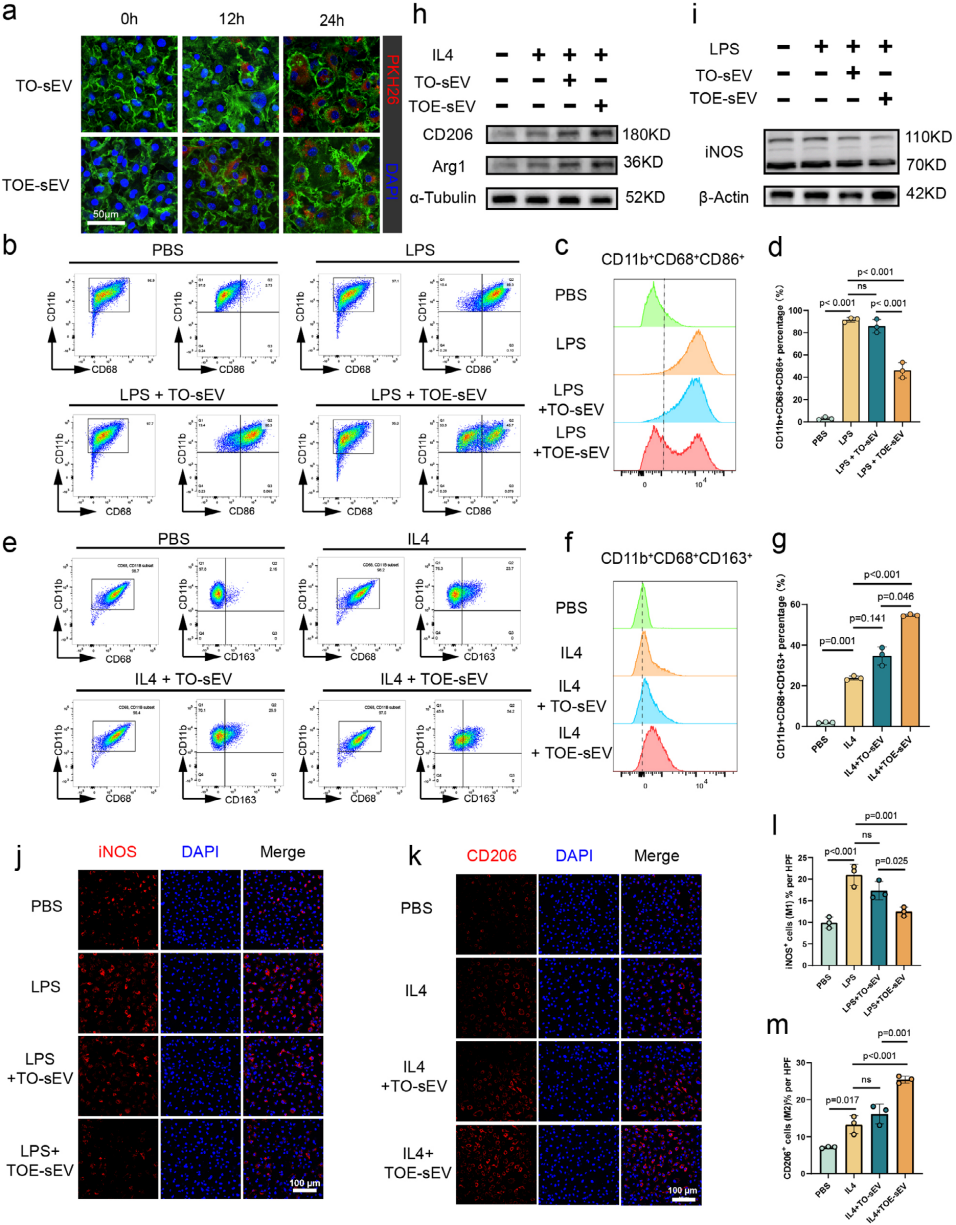
Plasma‐derived sEVs from the TOE group exhibit immunomodulatory activities in vitro. (a) PKH26‐labeled TO‐ and TOE‐derived sEVs were internalized by BMDMs within 12 and 24 h. (scale bar: 50 µm) (b–d) Flow cytometry was performed to evaluate the effects of TO‐sEVs and TOE‐sEVs on LPS‐induced M1 BMDM macrophage polarization. (e–g) Flow cytometry was performed to evaluate the effects of TO‐sEVs and TOE‐sEVs on IL4‐induced M2 BMDM macrophage polarization. (h) Western blot analysis examined the effects of TO‐sEVs and TOE‐sEVs on IL4‐induced M2 BMDM macrophage polarization marker expression (Arg1 and CD206). (i) Western blot analysis examined the effects of TO‐sEVs and TOE‐sEVs on LPS‐induced M1 BMDM macrophage polarization marker expression (iNOS). (j,l) Immunofluorescence imaging analysis was performed to measure the effects of TO‐sEVs and TOE‐sEVs on LPS‐induced M1 BMDM macrophage polarization. (scale bar: 100 µm, *n* = 3). (k,m) Immunofluorescence imaging analysis was performed to measure the effects of TO‐sEVs and TOE‐sEVs on IL‐4‐induced M2 BMDM macrophage polarization (scale bar: 50 µm, *n* = 3). Data are presented as the mean ± SD. ns, not significant. *p* < 0.05 was regard as statistically significant.

To explore the immunomodulatory properties of TOE‐sEVs, BMDMs were isolated and their polarization status was assessed following sEV treatment. First, PKH26 fluorescence labeling and subsequent co‐culture with BMDMs confirmed time‐dependent cellular internalization of both TO‐sEVs and TOE‐sEVs, with equivalent uptake efficiencies at 24 h (Figure 3a). Flow cytometry analysis verified the high purity of isolated BMDMs, with over 95% of cells being double‐positive for CD68 and CD11b, which were established surface markers for macrophages. Furthermore, LPS stimulation significantly upregulated M1 macrophage markers in BMDMs, while TO‐sEV treatment had minimal impact on this M1 polarization. In contrast, TOE‐sEV treatment significantly attenuated LPS‐induced M1 polarization. Similarly, IL‐4 stimulation enhanced M2 macrophage marker expression, which was not substantially altered by TO‐sEVs but was markedly augmented by TOE‐sEVs (Figure 3b–g). Western blot analysis corroborated these findings, showing that LPS‐induced M1 marker expression (iNOS) was significantly reduced by TOE‐sEVs but not by TO‐sEVs. Similarly, IL‐4‐induced M2 marker expression (Arg1, CD206) was significantly enhanced by TOE‐sEVs compared to TO‐sEVs (Figure 3h,i). Immunofluorescence staining of M1 (iNOS+) and M2 (CD206+) BMDMs consistently confirmed that TOE‐sEVs, but not TO‐sEVs, significantly suppressed LPS‐driven M1 polarization and potentiated IL‐4‐mediated M2 polarization (Figure 3j–m). Collectively, these results demonstrated that TOE‐sEVs possessed potent regenerative and immunomodulatory properties in vitro, significantly reducing LPS‐induced M1 polarization while enhancing IL‐4‐mediated M2 polarization.

### 3.3 Plasma‐Derived sEV from the TOE Group Enhanced In Vivo Wound Healing with Angiogenesis and Modulation of Macrophage Polarization

**FIGURE 4:**
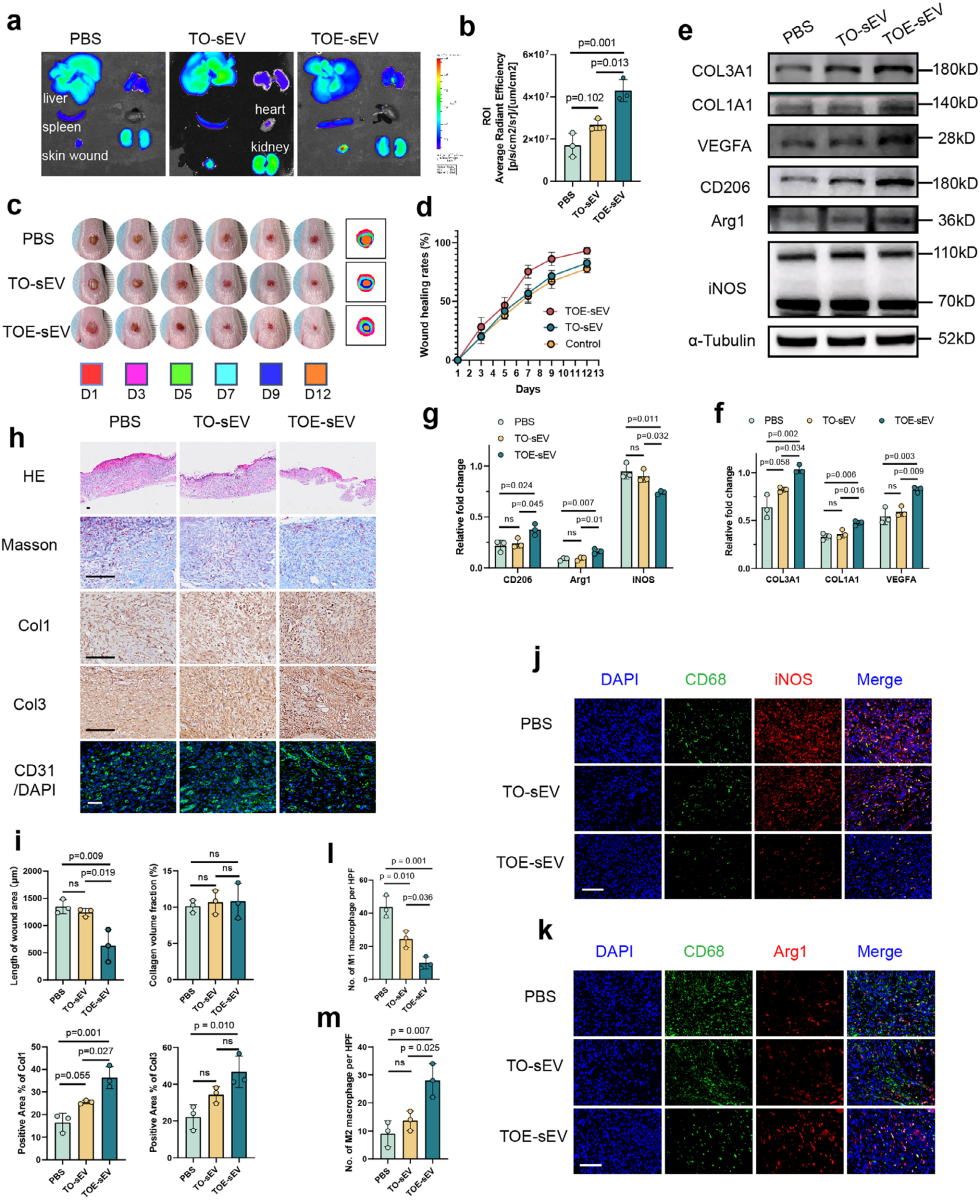
Plasma‐derived sEV from the TOE group enhanced in vivo wound healing with angiogenesis and modulation of macrophage polarization. (a,b) In vivo tracking of Dil‐labeled TO‐sEVs and TOE‐sEVs at 24 h post‐intravenous administration (day 2 post‐wounding) revealed enhanced tropism of TOE‐sEVs to skin wound sites compared to TO‐sEVs. (*n* = 3) (c) Gross images of rat dorsal foot wounds at days 1, 3, 5, 7, 10, and 12 post‐injury showed the healing progression following TO‐sEV or TOE‐sEV administration via tail vein injection on days 2 and 4 after wound creation. (*n* = 6) (d) Quantitative analysis of wound healing rates across the three groups was performed using ImageJ software. (*n* = 6) (e–g) Western blot analysis of wound tissues day collected on day 5 post‐injury revealed differential expression of COL3A1, COL1A1, iNOS, CD206, Arg1 and VEGFA among PBS, TO‐sEV, and TOE‐sEV groups. (*n* = 3) (h,i) Representative histological images (H&E and Masson staining) and immunohistochemical/immunofluorescence staining (Col1, Col3, and CD31) with corresponding quantitative analyses are presented for the PBS, TO‐sEV, and TOE‐sEV group. (scale bars: 100 µm, *n* = 3). (j–m) Immunofluorescence analysis of wound tissues at day 5 post‐injury demonstrated differential iNOS (M1 macrophage marker) and CD206 (M2 macrophage marker) expression levels among the PBS, TO, and TOE groups. (scale bars: 100 µm, *n* = 3) Data are presented as the mean ± SD. ns, not significant. *p* < 0.05 was regard as statistically significant.

To investigate the in vivo therapeutic effects of plasma‐derived sEVs, a dorsal foot skin defect model was established using nine 8‐week‐old SD rats. On days 2 post‐injury, DiI‐labeled sEVs were administered via tail vein injection. After a 24 h circulation period, tissue samples from major organs and wound sites were harvested for fluorescence imaging to evaluate sEV biodistribution. Both TO‐sEV and TOE‐sEV exhibited chemotactic targeting capability to the wound site. However, quantitative analysis revealed significantly higher fluorescence intensity of TOE‐sEV compared to TO‐sEV in the wound area, indicating superior accumulation efficiency of TOE‐derived sEV (Figure 4a,b).

Aligned with the timing of rESWT applied to tibial bone flaps, TOE‐sEV injections were administered on days 2 and 4 post‐injury. Our findings revealed that TOE‐sEV treatment significantly accelerated wound healing compared to the PBS group but not to the TO group on days 5 post‐injury, whereas on days 7, 10, and 12 post‐injury, differences were observed compared to both the PBS and TO groups (Figure 4c,d; and Table S2). On days 5 post‐injury, TOE‐sEV‐treated wound tissues exhibited decreased expression of the M1 macrophage marker iNOS and increased expression of the M2 macrophage marker CD206, accompanied by upregulated expression of VEGFA, Col1, and Col3, compared to both the PBS and TO groups (Figure 4e–g).

Histopathological analysis revealed significantly increased epidermal thickness, and collagen deposition in the TOE‐sEV group compared to the TO‐sEV and PBS control groups. Immunofluorescence staining of wound tissues further confirmed that TOE‐sEVs enhanced neovascularization, as indicated by increased CD31 expression, while TOE‐sEVs markedly reduced M1 macrophage marker expression and enhanced M2 macrophage marker compared to the TO‐sEV and PBS groups (Figure 4h–m), underscoring their immunomodulatory role in vivo.

Collectively, these findings demonstrated that TOE‐sEVs promoted skin wound healing in vivo by enhancing angiogenesis and modulating the inflammatory microenvironment through a shift from M1 to M2 macrophage polarization.

### 3.4 The sEVs that Mediate the Pro‐Healing Effects of TOE Might Have Been Derived from MSCs

**FIGURE 5:**
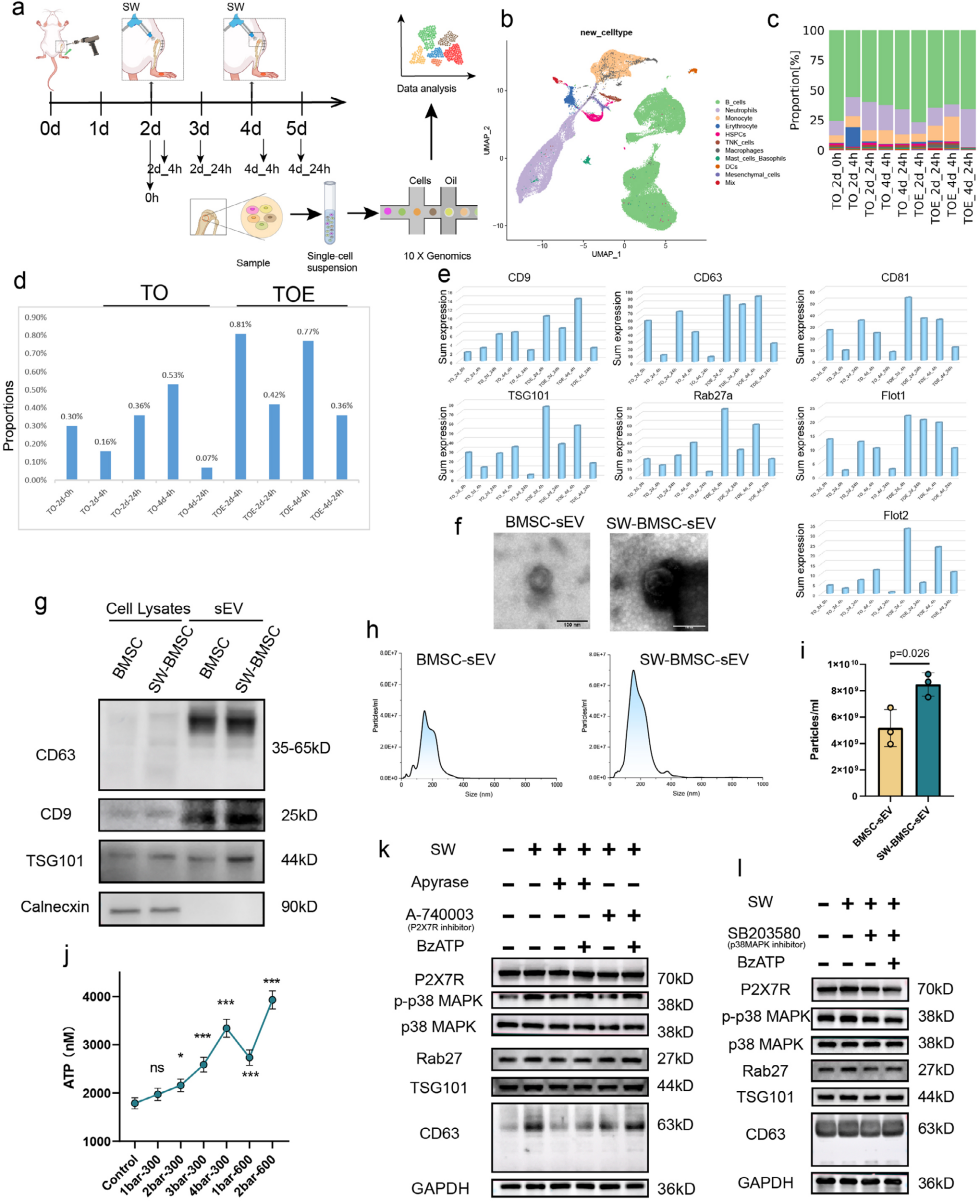
The sEVs that mediate the pro‐healing effects of TOE may be derived from BMSCs. (a) Schematic diagram illustrating single‐cell RNA sequencing (scRNA‐seq) performed on tissue samples from tibial osteotomy sites collected at 4 and 24 h following each shock wave treatment in the TOE group, with non‐treated samples from the TO group serving as controls. (b) UMAP embedding of all sampled cells with annotated clusters. (c) Proportions of indicated cell types in each group. TO‐2d‐0 h denotes the TO group at 2 days post‐injury without shock wave treatment, while TO‐2d‐4 h and TOE‐2d‐4 h serve as comparators, representing samples from the tibial osteotomy site collected 4 h after shock wave application on day 2 post‐injury. Similarly, TO‐2d‐24 h and TOE‐2d‐24 h indicate samples obtained 24 h after shock wave application on day 2 (day 3 post‐injury). The conditions for day 4 post‐injury follow an analogous pattern. (d) Proportions of mesenchymal stem cells in each group. (e) Bar chart showed the overall expression levels of exosomal markers (CD9, CD63, CD81, TSG101, Rab27a, Flot1, and Flot2) in mesenchymal stem cells (calculated as the product of expression and cell counts). (f) TEM images of sEVs extracted from BMSC cells subjected to shock wave (SW‐BMSC‐sEV) stimulation and from unstimulated BMSC cells (BMSC‐sEV as a control). (g) Western blot analysis confirmed the presence of sEV markers (CD63, CD9, and TSG101) in BMSC‐sEV and SW‐BMSC‐sEV groups. (h,i) NTA analysis revealed a monodisperse size distribution of sEVs (peak < 200 nm) both in BMSC‐sEV and SW‐BMSC‐sEV groups, with the particle concentration of sEVs extracted from BMSC cells subjected to SW stimulation exhibiting significantly higher compared to cells without SW stimulation. (*n* = 3) (j) ATP concentrations in BMSC cell supernatants following shock wave stimulation at varying pressures and pulse numbers. (k,l) SW stimulation enhances the expression of exosomal markers (CD63, TSG101, and Rab27), but this effect was attenuated by ATP hydrolase, P2X7R inhibitor, and p38 MAPK inhibitor. Supplementation with BzATP alleviates this inhibition, indicating that SW may promote sEV release through the ATP‐P2X7R‐p38MAPK signaling pathway.

To elucidate the cellular origins of sEVs responsible for the pro‐healing effects of the TOE, we performed single‐cell RNA sequencing (scRNA‐seq) on tissue samples collected at 4 and 24 h after each shock wave treatment (TOE group), with non‐treated samples (TO group) serving as controls (Figure 5a). Following clustering and annotation, scRNA‐seq identified 11 distinct cell populations, with their proportional distributions shown in Figure 5b,c and Figure S3. The proportion of mesenchymal cells (MSCs) was significantly higher at 4 and 24 h following each shock wave stimulation (TOE) compared to the non‐stimulated control group (TO). Furthermore, although the violin plots revealed no discernible pattern in the distribution of exosomal markers (CD9, CD63, CD81, TSG101, Rab27a, Flot1, and Flot2) across different cell types (Figure S5), the product of expression levels of these markers and cell numbers in MSCs exhibited a consistent upward trend, suggesting that MSCs likely served as the primary cellular source of the therapeutic sEVs mediating wound healing effects (Figure 5d,e).

Subsequently, we isolated primary bone marrow‐derived mesenchymal stem cells (BMSCs) and performed shock wave stimulation to validate their capacity for sEV secretion. To further confirm the capacity of BMSCs to secrete sEVs, primary BMSCs at passage 2 (P2) were subjected to rESWT (3 bar, 300 pulses, 5 Hz), and sEVs were isolated from culture supernatants 48 h post‐stimulation. Both shock wave‐treated (SW‐BMSC‐sEVs) and untreated control sEV (BMSC‐sEVs) displayed characteristic exosomal features, including typical cup‐shaped or spherical morphology with a bilayered membrane structure under the TEM analysis, positive expression of exosomal markers (CD63, CD9, and TSG101), and absence of Calnexin. NTA confirmed a monodisperse size distribution with peak diameters under 200 nm for both groups. Notably, the SW‐BMSC‐sEV group exhibited significantly higher particle concentrations compared to the BMSC‐sEV group (Figure 5f–i), reinforcing the hypothesis that BMSCs served as a key source of functional sEVs in TOE‐mediated wound healing.

To explore the molecular mechanisms underlying rESWT‐induced sEV secretion, we conducted Gene Ontology (GO) and KEGG pathway analysis of differentially expressed genes in MSCs after the TOE treatments (Figure S4). Across the three pairwise comparisons (TO‐2d‐4 h vs. TOE‐2d‐4 h, TO‐2d‐24 h vs. TOE‐2d‐24 h, and TO‐4d‐24 h vs. TOE‐4d‐24 h), KEGG pathway enrichment consistently highlighted the MAPK signaling pathway. In the TO‐2d‐24 h vs. TOE‐2d‐24 h comparison, GO biological process (BP) terms were primarily enriched in “response to mechanical stimulus.” In the TO‐4d‐24 h vs. TOE‐4d‐24 h comparison, BP enrichments were predominantly associated with positive regulation of transcription by RNA polymerase II and negative regulation of NF‐κB transcription factor activity. This suggested that shock wave stimulation on days 2 post‐injury might have primarily elicited a mechanical stimulus response in MSCs at the tibial osteotomy site, whereas stimulation on days 4 post‐injury might have induced transcriptional modulation and inflammatory control activities (Figure S4). Building on prior findings [31], we subsequently examined whether SW promoted sEV release through activation of the ATP/P2X7R/p38MAPK axis. The sEV secretion capacity was indirectly assessed by intracellular levels of exosome‐related markers (CD63, TSG101, and Rab27). Our results demonstrated that SW (3 bar, 300 pulses, 5 Hz) significantly increased extracellular ATP concentration in BMSC supernatants. Concurrently, SW significantly upregulated the expression of exosomal marker proteins and phosphorylated p38MAPK (p‐p38MAPK), while P2X7 receptor (P2X7R) expression remained negligible. However, these effects were attenuated by treatment with apyrase, an ATP‐hydrolyzing enzyme. Notably, the suppression of exosomal marker expression and p‐p38MAPK levels was effectively rescued by BzATP, a P2X7R agonist. Similarly, inhibition of P2X7R or p38MAPK reduced exosomal marker protein and p‐p38MAPK levels, but these effects were reversed by BzATP supplementation (Figure 5j–l). These findings collectively indicated that BMSCs might have been a primary source of therapeutic sEVs mediating wound‐healing effects, and the enhanced sEV secretion induced by SW was regulated through the ATP/P2X7R/p38MAPK signaling axis.

### 3.5 Proteomic Analysis Revealed Distinct Protein Profiles between TO‐sEV and TOE‐sEV

**FIGURE 6:**
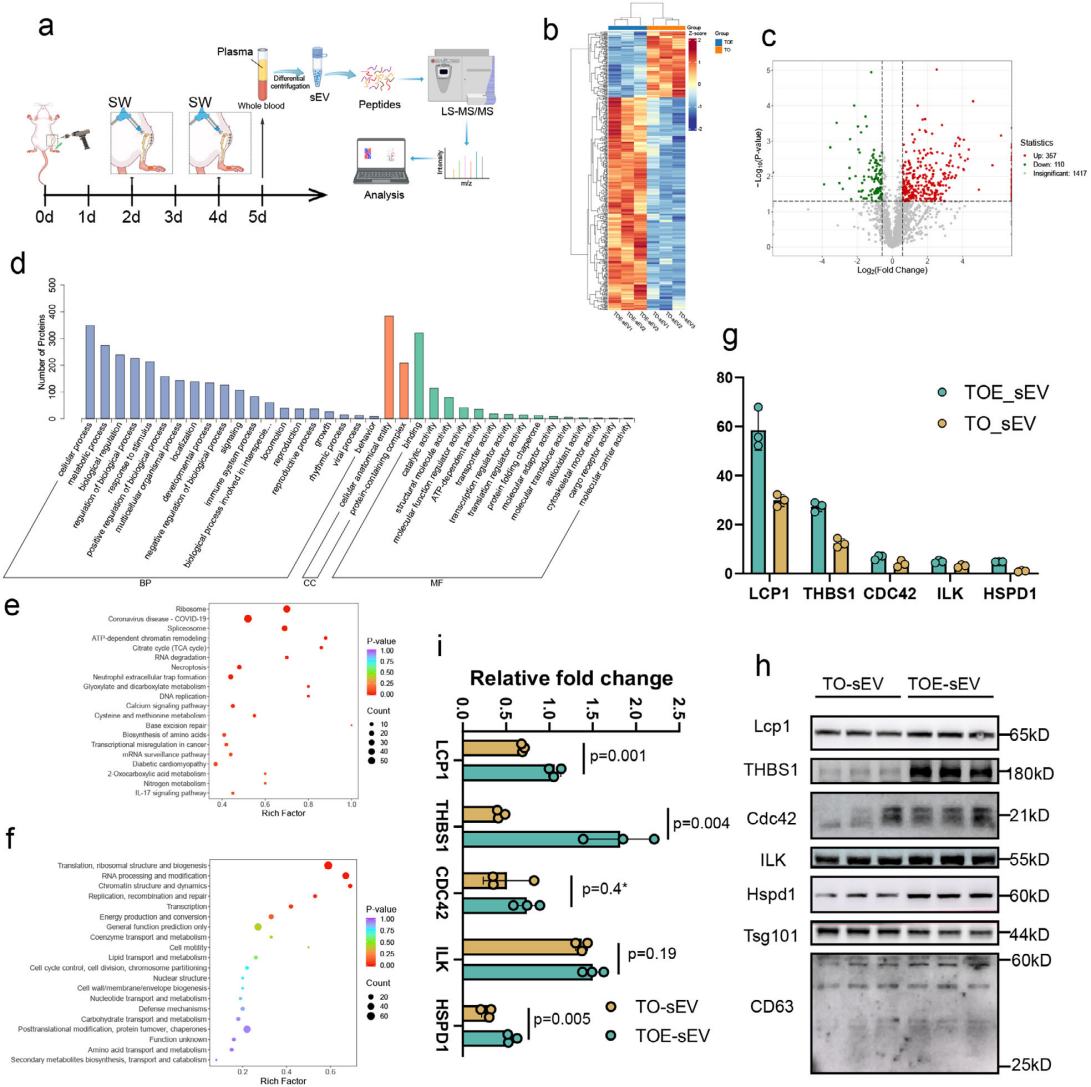
Proteomic analysis revealed distinct protein profiles between TO‐sEV and TOE‐sEV. (a) Schematic diagram illustrating the experimental workflow involving sEV isolation from day 5 post‐injury whole blood of TO and TOE groups, followed by proteomic analysis. (b) Heatmap analysis revealed the upregulated and downregulated proteins between the TOE‐sEV and TO‐sEV groups. (*n* = 3) (c) Volcano plot showed significantly upregulated and downregulated proteins between the TOE‐sEV and TO‐sEV groups. (d) Gene Ontology (GO) enrichment analysis of differentially expressed proteins was performed across three functional categories: biological process (BP), cellular component (CC), and molecular function (MF). (e) KEGG pathway enrichment analysis of total differentially expressed proteins. (f) KOG pathway enrichment analysis of total differentially expressed proteins. (g) Five candidate proteins were selected from the differentially expressed protein pool for further functional validation studies. (h,i) Western blot analysis was performed to validate the expression levels of the five candidate proteins in TO‐sEV and TOE‐sEV group. (*n* = 3) * indicated that statistical analysis was performed using the Mann‐Whitney U test. The remaining data were normally distributed, and presented as the mean ± SD. ns, not significant. *p* < 0.05 was regard as statistically significant.

Plasma‐derived sEVs were purified from TO and TOE groups at 24 h post‐second rESWT (day 5 post‐injury) and were subjected to quantitative proteomic profiling using data‐independent acquisition (DIA) mass spectrometry (Figure 6a). The volcano plot and heatmap analyses revealed that there were 357 significantly upregulated and 110 downregulated proteins in TOE‐sEVs compared to TO‐sEVs, based on a fold change (FC) threshold of ≥1.5 or ≤0.6667 and a significance level of *p* < 0.05 (Figure 6b,c). GO enrichment analysis of differentially expressed proteins showed that “cellular process” was the predominant category in biological processes, “cellular anatomical entity” in the cellular components, and “binding” and “catalytic activity” in the molecular functions, suggesting potential roles in cellular regulation and metabolic modulation (Figure 6d). KEGG pathway analysis revealed significant enrichment in the “Ribosome” pathway, while Clusters of Orthologous Groups (KOG) classification highlighted predominant enrichment in “Translation, ribosomal structure and biogenesis” (Figure 6e,f). These findings consistently underscored the central role of ribosome‐related functions and translational processes in TOE‐sEV‐mediated effects.

Based on proteomic statistical criteria (FC >1.5 and *p* < 0.05) and functional relevance to wound healing processes, we identified five significantly upregulated candidate proteins (Lcp1, Thbs1, Cdc42, ILK, and Hspd1) for subsequent functional validation (Figure 6g). Western blot analysis confirmed significant upregulation of Lcp1, Thbs1, and Hspd1 in TOE‐sEVs (all *p* < 0.05), consistent with the proteomic findings. However, no significant differences were observed in the expression levels of ILK and Cdc42 between the TO‐sEV and TOE‐sEV groups, indicating partial validation of the proteomic data (Figure 6h,i). This discrepancy might primarily arise from methodological differences between DIA mass spectrometry and Western blot analysis.

### 3.6 The Thbs1‐enriched Engineered sEV Enhanced Angiogenesis and Macrophage M2 Polarization In Vitro

**FIGURE 7:**
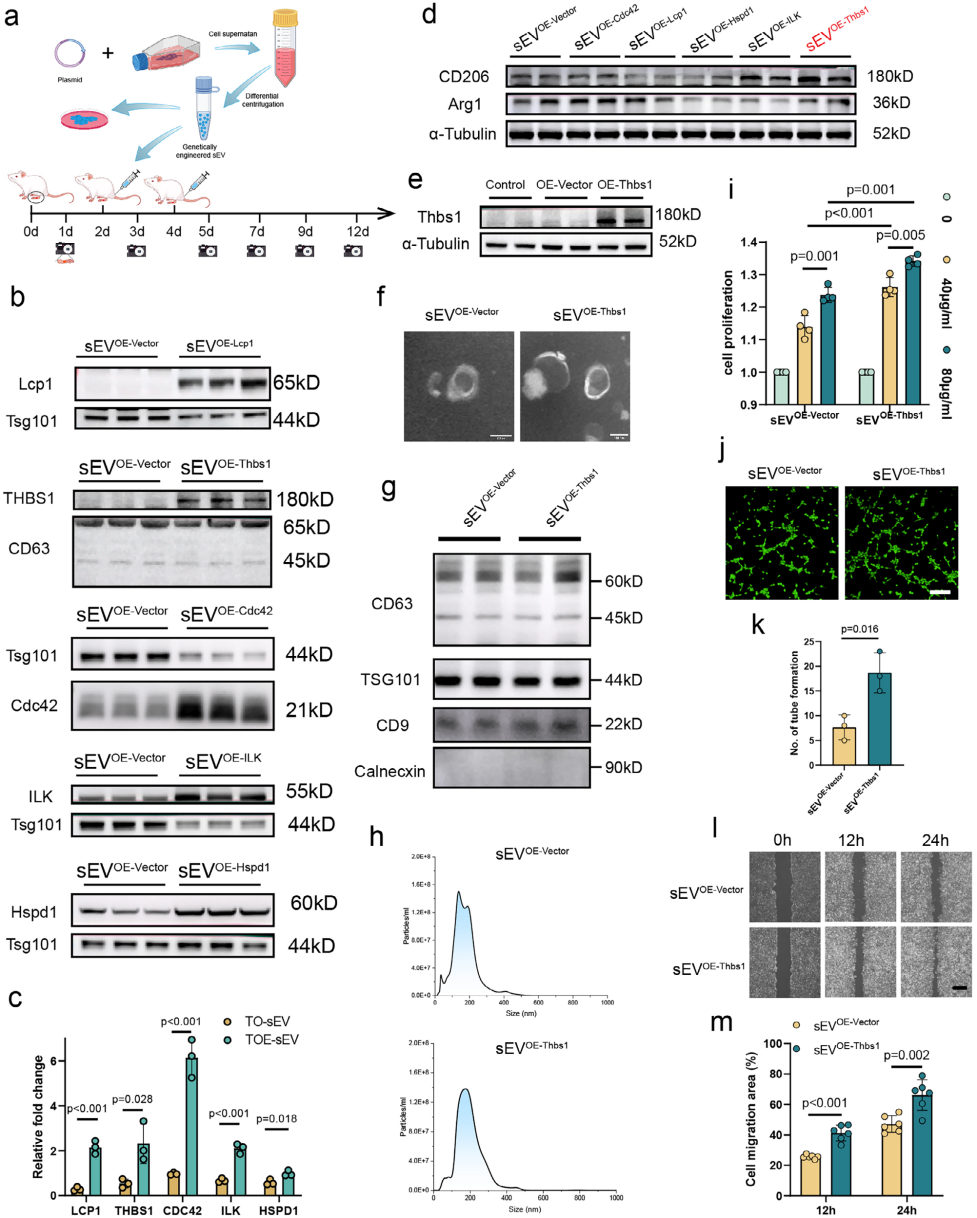
The Thbs1‐enriched engineered sEV exhibited regenerative activities in vitro. (a) Schematic diagram illustrated that supernatant‐derived engineered sEVs from 293T cells overexpressing five differentially expressed candidate proteins were isolated for subsequent in vitro and in vivo functional validation experiments. (b,c) Western blot analysis was performed to validate the expression of corresponding target proteins in engineered sEVs isolated from 293T cells overexpressing the five candidate genes. (*n* = 3) (d) The engineered sEVs overexpressing five candidate genes were applied to IL‐4‐induced BMDM cells for 24 h to evaluate alterations in M2 polarization markers (Arg1 and CD206). Thbs1‐loaded sEVs (sEV^OE‐Thbs1^) increased expression of Arg1 and CD206 markers. (e) Western blot analysis of total Thbs1 expression in untransfected, empty vector‐transfected, and Thbs1‐overexpressing 293T cells revealed negligible endogenous Thbs1 production, thereby eliminating potential confounding effects from intrinsic Thbs1 activity in subsequent functional studies. (f) Transmission electron microscopy (TEM) revealed characteristic cup‐shaped, double‐membrane structures of isolated from the sEV^OE‐Vector^ and sEV^OE‐Thbs1^ group. (scale bar: 100 nm) (g) Western blot analysis confirmed the presence of sEV markers (CD63, CD9, and TSG101) in the sEV^OE‐Vector^ and sEV^OE‐Thbs1^ groups. (h) NTA analysis revealed size distributions (a primary peak < 200 nm) of sEVs across the sEV^OE‐Vector^ and sEV^OE‐Thbs1^ groups. (i) CCK‐8 assay revealed the time‐dependent effects of sEVs on HUVEC viability at 24 h post‐treatment. (*n* = 4) (j,k) Tube formation assay demonstrated the differential angiogenic potential of the sEV^OE‐Vector^ and sEV^OE‐Thbs1^ group. (scale bar: 200 µm, *n* = 3) (l,m) Quantitative analysis of scratch wound closure revealed that sEV^OE‐Thbs1^ enhanced HUVEC cells migration compared to sEV^OE‐Vector^ at 12 and 24 h. (scale bar: 500 µm, *n* = 6) Data are presented as the mean ± SD. ns, not significant. *p* < 0.05 was regard as statistically significant.

To investigate the functional roles of specific proteins in sEV‐mediated wound healing. HEK293T cells were selected for their high transfection efficiency and sEV yield. These cells were engineered to overexpress Lcp1, Thbs1, Cdc42, ILK, and Hspd1. These sEVs were subsequently isolated from the cell supernatant for in vitro and in vivo functional studies (Figure 7a).

Optimal overexpression conditions were established by systematically evaluating protein expression levels in HEK293T cells cultured in 10 cm dishes using varying plasmid amounts (0, 1, 6, 12, and 24 µg) and transfection durations (24 and 48 h) for each target gene (Lcp1, Thbs1, Cdc42, ILK, and Hspd1). Western blot analysis identified the specific plasmid dose and time point that yielded maximal target protein expression, which were subsequently adopted for sEV production (Figure S6).

Following 48 h of transfection, sEVs were isolated from the conditioned media of HEK293T cells transfected with plasmids encoding Lcp1, Thbs1, Cdc42, ILK and Hspd1. Western blot analysis confirmed significantly elevated levels of each target protein in the respective engineered sEVs compared to empty vector controls, thereby verifying successful protein loading into sEVs (Figure 7b,c).

Preliminary screening of the five engineered sEVs for their effects on IL‐4‐induced M2 polarization of BMDMs revealed that Thbs1‐enriched sEVs (sEV^OE‐Thbs1^) uniquely enhanced the expression of M2 markers compared to control sEV (sEV^OE‐Vector^). Consequently, the sEV^OE‐Thbs1^ were prioritized for further mechanistic studies in in vitro and in vivo wound healing models. To confirm the specificity of Thbs1 overexpression, total Thbs1 protein levels (endogenous and exogenous) were assessed in untransfected HEK293T cells, empty vector‐transfected cells, and Thbs1‐overexpressing cells. Both untransfected and empty vector‐transfected cells exhibited negligible Thbs1 expression, thus ruling out interference from endogenous Thbs1 and eliminating the need for a Thbs1‐knockout control group in subsequent experiments (Figure 7d,e).

Both the sEV^OE‐Thbs1^and sEV^OE‐Vector^ exhibited characteristic cup‐shaped or discoid morphology with double‐layered membranes under the TEM analysis, and expressed exosomal markers (CD63, CD9, and TSG101), and were negative for Calnexin. NTA revealed a size distribution with a prominent primary peak below 200 nm, consistent with the expected sEV size range (Figure 7f–h).

**FIGURE 8:**
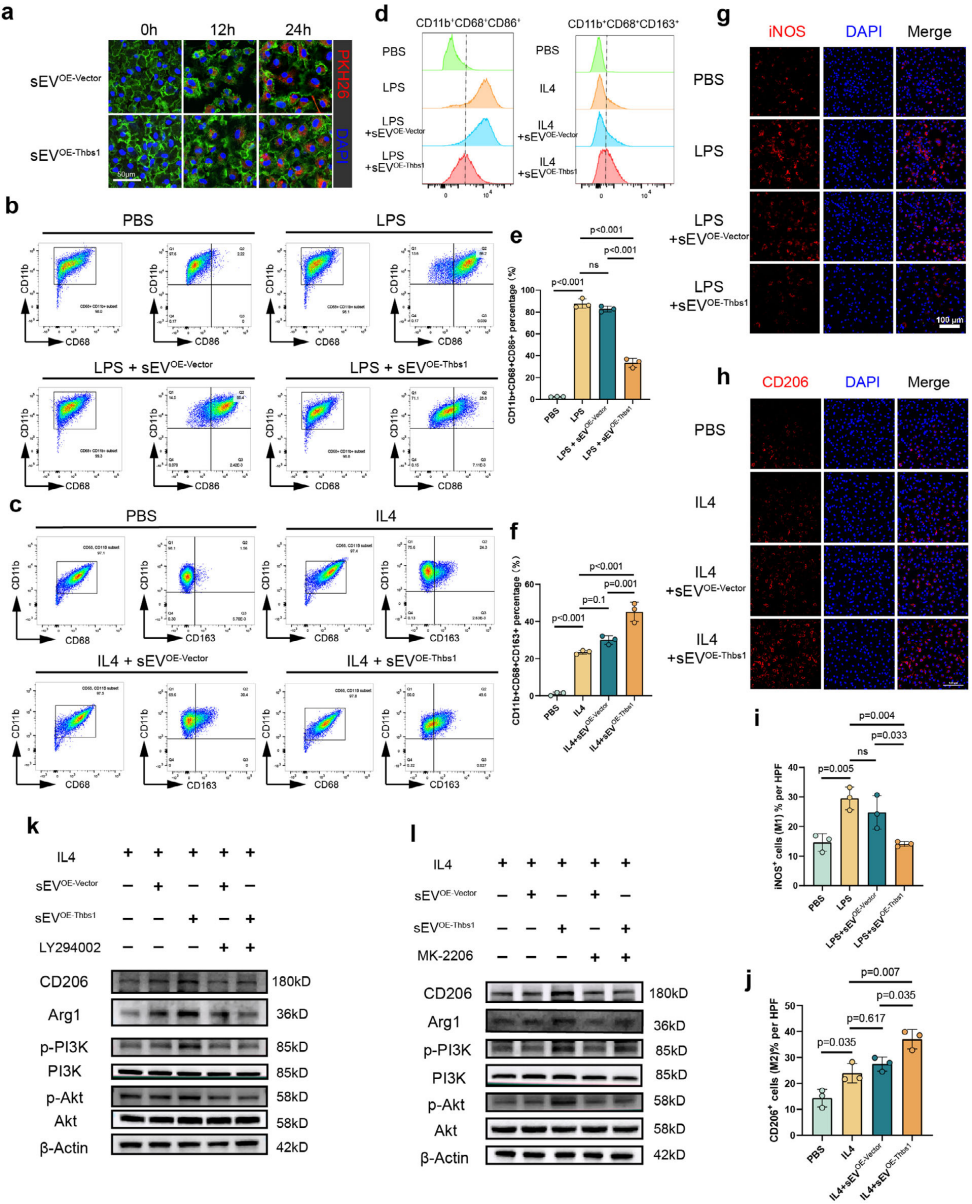
The Thbs1‐enriched engineered sEV exhibited immunomodulatory activities in vitro. (a) PKH26‐labeled the sEV^OE‐Vector^ and sEV^OE‐Thbs1^ group were internalized by BMDMs within 12 and 24 h. (Scale bar: 50 µm) (b,e) Flow cytometry was performed to evaluate the effects of sEV^OE‐Vector^ and sEV^OE‐Thbs1^ on LPS‐induced M1 BMDM macrophage polarization. (*n* = 3) (c,f) Flow cytometry was performed to evaluate the effects of sEV^OE‐Vector^ and sEV^OE‐Thbs1^ on IL4‐induced M2 BMDM macrophage polarization. (*n* = 3) d. Flow cytometry histogram overlay revealed that sEV^OE‐Thbs1^ treatment significantly attenuated LPS‐induced M1 macrophage polarization while promoting IL‐4‐driven M2 macrophage polarization compared to sEV^OE‐Vector^ controls. (g,i) Immunofluorescence imaging analysis was performed to measure the effects of sEV^OE‐Vector^ and sEV^OE‐Thbs1^ on LPS‐induced M1 BMDM macrophage polarization. (scale bars: 100 µm, *n* = 3) (h,j) Immunofluorescence imaging analysis was performed to measure the effects of sEV^OE‐Vector^ and sEV^OE‐Thbs1^ on IL‐4‐induced M2 BMDM macrophage polarization. (scale bars: 100 µm, *n* = 3) (k,l) Western blot analysis was performed to evaluate the effects of sEV^OE‐Vector^ and sEV^OE‐Thbs1^ on IL‐4‐induced M2 BMDM macrophage polarization markers (Arg1 and CD206) and PI3K/AKT signaling pathway activation. Data are presented as the mean ± SD. ns, not significant. *p* < 0.05 was regard as statistically significant.

In HUVECs, sEV^OE‐Vector^ promoted cellular proliferation in a dose‐dependent manner, and enhanced migratory capacity and tube‐forming ability in Matrigel assays (Figure 7i–m). Both the sEV^OE‐Vector^ and sEV^OE‐Thbs1^ were efficiently internalized by BMDMs, as confirmed by fluorescence tracking (Figure 8a). Flow cytometric analysis demonstrated that while sEV^OE‐Vector^ treatment had no significant modulatory effect on this LPS‐induced M1 polarization, sEV^OE‐Thbs1^ potently suppressed M1 marker expression. Conversely, sEV^OE‐Vector^ showed no modulatory effect, whereas sEV^OE‐Thbs1^ synergistically enhanced IL‐4‐mediated M2 polarization (Figure 8b–f).

Immunofluorescence analysis further elucidated the regulatory effects of thses sEVs on macrophage polarization dynamics. Treatment with sEV^OE‐Thbs1^ significantly attenuated LPS‐induced M1 polarization, whereas sEV^OE‐Vector^ exhibited no significant modulatory effect. Conversely, in the M2 polarization paradigm, sEV^OE‐Thbs1^ synergistically enhanced IL‐4‐induced M2 polarization, as evidenced by increased expression of M2 markers. In contrast, sEV^OE‐Vector^ showed no significant impact on M2 marker expression, consistent with its lack of effect on M1 polarization (Figure 8g–j). These findings collectively demonstrated the selective immunomodulatory capacity of sEV^OE‐Thbs1^ in bidirectional regulation of macrophage polarization, promoting a shift from an M1 to an M2 phenotype. Western blot analysis corroborated these findings, showing that LPS‐induced iNOS expression was significantly reduced by sEV^OE‐Thbs1^ but remained unaffected by sEV^OE‐Vector^ treatment. In contrast, sEV^OE‐Thbs1^ significantly augmented IL‐4‐induced expression of M2 markers (Arg1 and CD206) compared to sEV^OE‐Vector^. Importantly, we found that the sEV^OE‐Thbs1^‐mediated promotion of IL‐4‐induced M2 polarization was associated with activation of the PI3K/AKT pathway. Pharmacological inhibition of either PI3K (LY294002) or AKT (MK‐2206) effectively suppressed the upregulation of M2 markers, indicating that sEV^OE‐Thbs1^ regulated macrophage polarization, at least in part, through the PI3K/AKT signaling pathway in IL‐4‐stimulated BMDMs (Figure 8k,l).

### 3.7 The Thbs1‐Enriched Engineered sEV Promote Wound Healing with Angiogenesis and Modulation of Macrophage Polarization In Vivo

**FIGURE 9:**
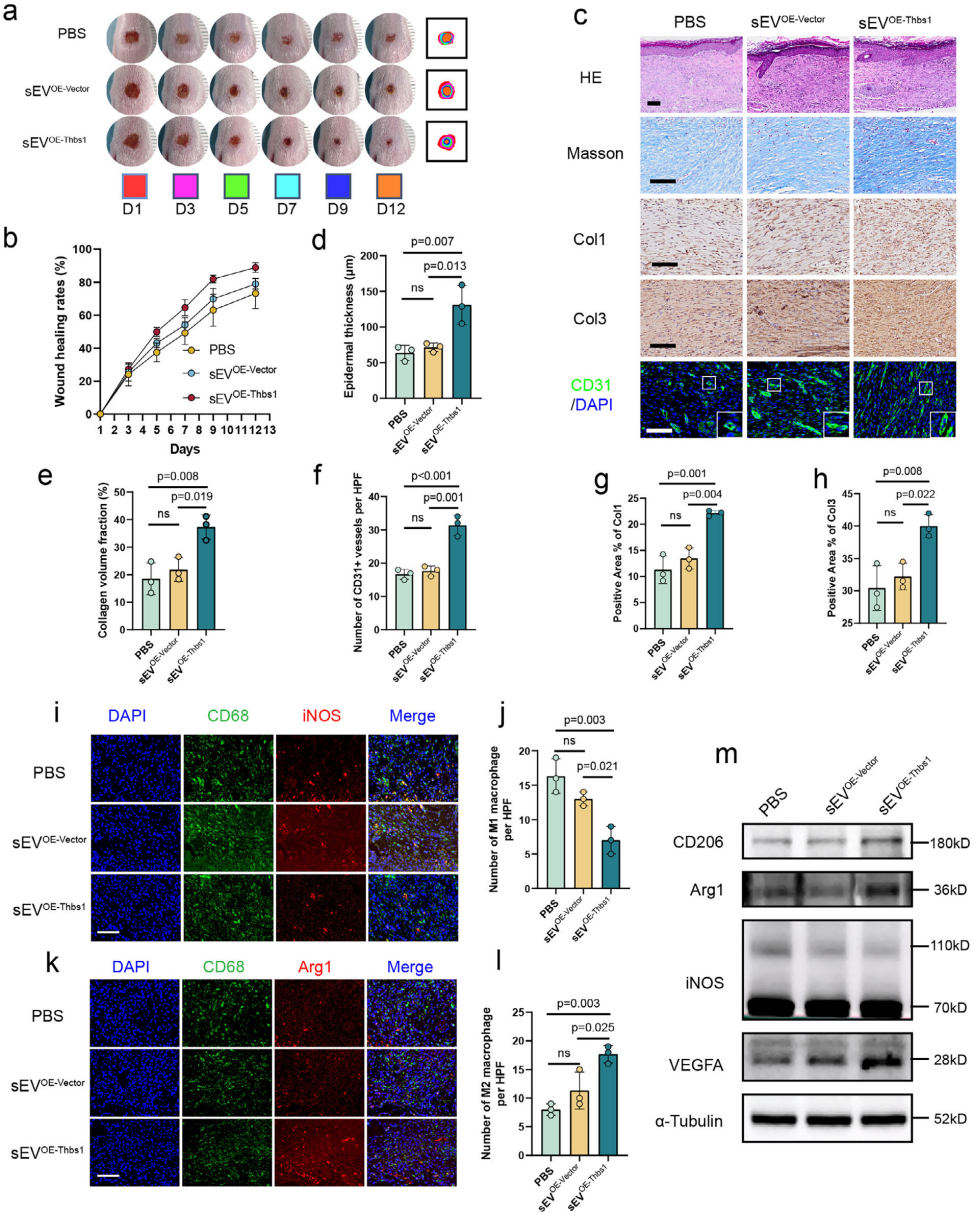
The Thbs1‐enriched engineered sEV promote wound healing with angiogenesis and modulation of macrophage polarization in vivo. (a) Gross images of rat dorsal foot wound healing progression on days 1, 3, 5, 7, 10, and 12 post‐injury demonstrated that local administration of sEV^OE‐Thbs1^ on postoperative days 2 and 4 significantly enhanced wound closure rates compared to sEV^OE‐Vector^ controls. (b) Quantitative analysis of wound healing rates across the five groups was performed using ImageJ software. (*n* = 6) (c–h) Representative histological images (H&E and Masson staining) and immunohistochemical/immunofluorescence staining (Col1, Col3, and CD31) with corresponding quantitative analyses are presented for three groups at day 12 post‐injury. (Scale bars: 100 µm, *n* = 3). (i–l) Immunofluorescence analysis of wound tissues collected on day 5 post‐injury demonstrated differential expression levels of iNOS (M1 macrophage marker) Arg1 and CD206 (M2 macrophage marker) and among the PBS, sEV^OE‐Vector^ and sEV^OE‐Thbs1^ groups. (scale bars: 100 µm, *n* = 3) (m) Western blot analysis of wound tissues collected on day 5 post‐injury revealed differential expression of COL3A1, COL1A1, iNOS, CD206, Arg1, and VEGFA among the PBS, sEV^OE‐Vector^ and sEV^OE‐Thbs1^ groups. Data are presented as the mean ± SD. ns, not significant. *p* < 0.05 was regard as statistically significant.

A full‐thickness excisional wound model was established on the dorsal foot of eighteen 8‐week‐old SD rats. Engineered sEVs were administered via local injection into the wound bed on days 2 and 4 post‐injury. On days 5, 7, 9, and 12 post‐injury, the sEV^OE‐Thbs1^ group showed significantly accelerated wound closure relative to both the sEV^OE‐Vector^ and PBS control groups. No significant difference was observed between the sEV^OE‐Vector^ and PBS groups at these time points (Figure 9a,b; and Table S3). Histopathological evaluation revealed that the sEV^OE‐Thbs1^ group exhibited superior tissue regeneration relative to both sEV^OE‐Vector^ and PBS groups. The sEV^OE‐Thbs1^ group exhibited significantly increased epidermal thickness, augmented collagen deposition, enhanced neovascularization as assessed by CD31 immunofluorescence, and upregulated expression of Col1 and Col3 (Figure 9c–h). Immunofluorescence analysis at day 5 post‐injury demonstrated distinct macrophage polarization patterns among the treatment groups. The PBS group displayed the highest accumulation of LPS‐induced M1 macrophages (iNOS^+^cells), while the sEV^OE‐Vector^ group failed to significantly modulate M1 macrophage populations. In contrast, the sEV^OE‐Thbs1^ group effectively reduced M1 macrophage infiltration, while markedly increasing the proportion of IL4‐induced M2 macrophages (CD206^+^cells), an effect not observed with sEV^OE‐Vector^ (Figure 9i–l). Western blot analysis corroborated these findings, showing that sEV^OE‐Thbs1^ upregulated expression of M2 markers (Arg1 and CD206) and VEGFA in IL4‐induced BMDM while downregulating the M1 marker (iNOS) expression in LPS‐induced BMDM compared to both PBS and sEV^OE‐Vector^ groups (Figure 9m). These findings collectively suggested that the sEV^OE‐Thbs1^ might have promoted cutaneous wound healing through dual mechanisms of enhancing angiogenesis and modulating macrophage polarization.

## 4 Discussion

This study established a novel mechanobiological paradigm wherein rESWT application to the tibial osteotomy site (TOE) systemically enhanced distant wound healing via sEV‐mediated signaling. This approach offered a promising less‐invasive clinical alternative for challenging wound cases, particularly in diabetic or aged patients with impaired healing capacity.

Wound healing is a complex process influenced by numerous endogenous and exogenous factors. Imperfect healing can lead to dysregulated inflammatory responses, excessive fibrosis, or uncontrolled cell proliferation, thereby contributing to pathological conditions such as cancer, fibrosis, or chronic inflammation [32]. Conventional wound management strategies such as debridement, skin transplantation, advanced dressing applications, and hyperbaric oxygen therapy have remained constrained by prolonged healing processes, substantial financial burdens, immunological incompatibility, and frequent infectious complications, collectively driving the demand for innovative regenerative solutions [5, 6].

Recently, the modified Ilizarov tibial cortical transverse transport (TTT) technique, which leverages the tension‐stress effect, has been successfully employed in clinical practice for years to treat diabetic foot ulcers and lower limb ischemic ulcers [9, 33], with reported healing rates as high as 90% [34, 35]. Intriguingly, Li et al. extended this approach to cranial bone transport (CBT), which enhanced meningeal lymphatic drainage to mitigate ischemic stroke damage, promoted rapid neuronal functional recovery [36], and ameliorated Alzheimer's disease pathology [37].

Inspired by TTT, our study innovatively replaced the gradual mechanical distraction with rapid shock wave stimulation to evaluate its effects on wound healing, with the ultimate goal of improving diabetic foot ulcer treatment. Comparative analysis revealed that TOE achieved healing outcomes comparable to TTT, while offering advantages in minimal invasiveness and reduced costs associated with external fixation. To establish an effective rESWT protocol, we initially selected parameters based on their impact on BMSC and MC3T3E1 viability. Previous finite element analyses had indicated that higher radial shockwave pressure amplitudes enhanced pressure transmission to osseous tissues [29]; however, these studies did not consider cellular biological responses. In the present study, we systematically assessed the effects of varying pressure levels and impulse counts on the viability of BMSCs and MC3T3E1 in vitro. Based on these findings, an optimal stimulation protocol was established, utilizing a pressure of 3 bar, 300 impulses, and a frequency of 5 Hz. To contextualize the selection of rESWT, we compared our findings with prior studies that had investigated the effects of rESWT on mesenchymal stem cells. Notably, one study reported that a protocol of 2 bar, 600 impulses, and 5 Hz promoted subchondral bone progenitor cell self‐renewal [38]. Similarly, another study demonstrated that human mesenchymal stem cells treated with 2 bar, 1000 impulses, and 5 Hz exhibited maximal vitality after 11 days [39]. Discrepancies between these outcomes and our findings might have been attributed to differences in shock wave application methodologies. In our study, rESWT was delivered to BMSCs and MC3T3E1 osteoblasts suspended in Eppendorf tubes, whereas the referenced studies utilized a radial shock wave applicator to treat floating MSCs positioned below the liquid surface. Additionally, bone flap preservation in TOE significantly contributed to enhanced wound healing. Preliminary experiments on tibial osteotomy with bone flap preservation demonstrated accelerated wound healing following rESWT compared to non‐preserved groups, suggesting that preserved bone flaps might enhance deep tissue energy absorption from shock waves. In contrast, early soft tissue (e.g., blood clots) in non‐preserved groups might limit energy absorption, though this mechanical mechanism warranted further investigation.

Skin wound repair constitutes a highly complex and tightly regulated process that orchestrates the spatiotemporal reorganization of multiple cell types. Macrophages played a pivotal role in normal wound healing and tissue regeneration, serving as primary regulators of the inflammatory phase [40]. Monocytes were recruited to the wound site within 48–96 h post‐injury, where they differentiate into tissue‐activated macrophages [41]. During the early inflammatory phase, macrophages accumulating at ulcer sites exhibited a “classically activated” pro‐inflammatory M1 phenotype, characterized by expression of inflammatory cytokines and chemokines such as interleukin‐1β (IL‐1β), IL‐12, and tumor necrosis factor‐α (TNF‐α) [42]. To prevent excessive inflammatory responses, these macrophages subsequently polarized into an anti‐inflammatory M2 phenotype, secreting resolution mediators including IL‐10 and transforming growth factor‐β (TGF‐β) to promote inflammation resolution and tissue remodeling [43]. In our study, the TOE group exhibited a significant reduction in M1 macrophage markers and a concomitant increase in M2 markers compared to both TO and control groups at day 5 post‐injury. Notably, administration of the sEV inhibitor GW4869 reversed this pro‐reparative polarization, restoring M1 dominance and suppressing M2 populations, thus confirming the essential role of plasma‐derived sEVs in mediating TOE‐induced macrophage polarization in the wound tissues.

Extracellular vesicles can facilitate intercellular transfer of diverse functional cargoes, including proteins, lipids, microRNAs, other RNAs, and DNA [44, 45]. sEVs, ranging from 30–200 nm, are secreted by nearly all cell types [46]. Stem cell‐derived sEVs served as pivotal mediators of stem cell paracrine effects, offering an ideal cell‐free therapeutic strategy for wound healing [47]. Mesenchymal stem cell (MSC)‐derived sEVs carried pro‐angiogenic and immunomodulatory cytokines, such as VEGF, TGF‐β1, IL‐6, and IL‐10, which collectively enhanced vascularization and immune regulation [48]. Previous studies have reported that epidermal stem cell‐derived sEVs promoted alternative (M2) macrophage polarization, and accelerated diabetic ulcer healing [49, 50], while BMSC‐derived sEVs carrying miR‐146a‐5p promoted diabetic wound healing in mice by modulating macrophage M1/M2 polarization [51]. Analysis of scRNA‐seq data from the tibial osteotomy site indicated that MSCs might have served as the cellular source of sEVs mediating wound healing. Additionally, our study demonstrated that shock wave (SW) stimulation promoted the secretion of sEVs from BMSCs. The P2X7 receptor (P2X7R) is closely associated with extracellular vesicle secretion. Activation of P2X7R triggered downstream phosphorylation of p38 MAPK and activation of ROCK, thereby promoting the release of large extracellular vesicles. Furthermore, it induced NLRP3 inflammasome formation, facilitating the release of sEVs [52]. Our study indicated that SW stimulation enhanced extracellular ATP release, activated P2X7R and promoted p38 MAPK phosphorylation, which increased the expression of sEV markers, such as CD63 and TSG101. However, the mechanism by which p38 MAPK phosphorylation influenced sEV release required further investigation.

Our study demonstrated that sEVs isolated from the whole blood of the TOE group exhibited significant pro‐healing effects both in vitro and in vivo. Specifically, TOE‐sEVs enhanced HUVEC proliferation, migration, and tube formation, as well as the migration of HaCaT keratinocytes and HSFs. Furthermore, TOE‐sEVs modulated macrophage polarization by reducing LPS‐induced M1 markers and increasing IL‐4‐induced M2 markers. In vivo, TOE‐sEV treatment accelerated wound closure and shifted the macrophage balance toward a pro‐reparative phenotype. To further investigate which factors carried by TOE contributed to its effects, we performed proteomic analysis on sEVs.

Proteomic profiling of TOE‐sEVs versus TO‐sEVs identified five differentially expressed candidate proteins, which were subsequently investigated through engineered sEVs. Engineered sEVs, enhanced through genetic engineering, chemical modification, and polymer hybridization, offer improved targeting, stability, and drug‐loading capacity, with potential applications in cancer therapy, inflammatory diseases, and tissue regeneration [53]. Given their high EV secretion capacity and genetic tractability [54], HEK‐293T cells were selected as donor cells for sEV production. Critically, Thbs1‐overexpressing sEVs (sEV^OE‐Thbs1^) significantly enhanced IL‐4‐induced M2 polarization markers, promoted HUVEC proliferation, migration, and tube formation, and accelerated in vivo wound closure by concurrently modulating macrophage polarization and stimulating angiogenesis.

Thrombospondin‐1 (Thbs1, also known as TSP‐1) is a multifunctional extracellular matrix protein that regulates diverse biological processes, including tissue healing [55]. However, its role in wound healing remains controversial. Emerging evidence demonstrated Thbs1 inhibited cutaneous wound repair, with transgenic mice overexpressing Thbs1 in keratinocytes exhibiting delayed healing, reduced granulation tissue formation, and impaired angiogenesis [56]. Additionally, Thbs1 suppressed fibroblast migration in vitro and in vivo [56]. Compellingly, rescue experiments revealed that miR‐27b‐mediated Thbs1 downregulation restored bone marrow‐derived angiogenic cell function and accelerated wound healing [57], while miR‐221‐3p similarly promoted diabetic wound repair through Thbs1 targeting [58]. These collective findings established Thbs1 as a negative regulator of skin wound healing through its pleiotropic suppression of angiogenesis, fibroblast motility, and granulation tissue formation. Conversely, public single‐cell RNA sequencing data from murine wound skin revealed that Thbs1+ keratinocytes represented a distinct migratory subpopulation that actively promoted epidermal wound healing [59], and TSP‐1 deficiency resulted in impaired wound healing and prolonged inflammatory responses [60, 61]. Furthermore, accumulating evidence identified TSP‐1 as a critical regulator of immune responses [62]. In the presence of inflammatory cytokines, TSP‐1 accelerated neutrophil and macrophage apoptosis in a dose‐dependent manner, and enhanced macrophage‐mediated efferocytosis of neutrophils [63], consistent with prior findings that TSP‐1 served as a molecular bridge between neutrophils and macrophages to facilitate phagocytic clearance [61]. Notably, under anti‐inflammatory conditions (IL‐4/IL‐13 or IL‐10), TSP‐1 upregulated M2 macrophage markers (CD206 and Arg1), promoting a pro‐reparative (M2) phenotype [63].

These results are consistent with our findings that Thbs1‐overexpressing sEV significantly enhances IL‐4‐induced macrophage polarization, as evidenced by upregulated expression of M2 markers (CD206 and Arg1). Mechanistically, this effect appeared to be mediated through PI3K/Akt signaling pathway activation. This aligned with prior findings that Thbs1 strongly activated the PI3K pathway in glioblastoma [64] and oral squamous cell carcinoma [65].

### 4.1 Limitation of Studies

The current rESWT parameters for tibial osteotomy site stimulation were optimized based on the in vitro viability assays of BMSC and MC3T3E1 osteoblasts, it remained to be systematically investigated, however, whether these parameters would be optimal for promoting heterotopic wound healing, particularly in the context of diabetic foot wounds. Additionally, the mechanobiological mechanisms underlying rESWT‐induced effects at the osteotomy site remained unelucidated. Future studies should employ finite element modeling to characterize mechanical force distribution and cellular response thresholds at the osteotomy site.

While sEVs played a significant role in mediating TOE‐accelerated wound healing, the potential contributions of alternative signaling pathways remained unexplored. Additionally, the plasma sEVs isolated via differential ultracentrifugation may contain heterogeneous impurities, which could also confound the results. Future research will thus utilize commercial sEV isolation kits to achieve higher purity. Although sc‐RNA seq data suggested BMSCs as the primary source of pro‐healing sEVs, the ubiquitous expression of conventional sEV markers across multiple cell types necessitated rigorous validation using lineage‐specific genetic models.

Notably, stem cell‐derived sEVs represent an advanced therapeutic strategy for diabetic foot. However, their clinical application lacks standardized protocols, such as unified dosage and administration frequency, and the heterogeneity in sEVs composition may lead to variability in therapeutic efficacy. In this study, plasma‐derived sEVs were not used directly as therapeutics but served as carriers for TOE to enhance ectopic wound repair. Should sEVs be developed as stand‐alone therapeutic agents, further studies would be necessary to elucidate their targeting mechanisms, pharmacokinetic profiles, optimal dosing, and treatment duration. Compared to stem cell‐derived sEVs, the TOE model offers superior potential for procedural standardization, analogous to the established use of TTT combined with localized wound care for diabetic foot ulcers (DFU), thereby supporting more consistent and effective wound healing. At the same time, caution is warranted against potential complications, including wound infection, nonunion, and osteomyelitis.

The contributions of other sEV cargo components, such as non‐coding RNAs, lipids, or post‐translationally modified proteins, to wound healing remained uncharacterized, though we performed comprehensive proteomic profiling of TOE‐sEVs. Consequently, the mechanistic basis underlying TOE‐sEV‐mediated wound repair had not been fully elucidated. In subsequent studies, the components within TOE‐sEVs, such as microRNAs or mRNAs, should be systematically explored to further elucidate their roles in wound healing.

While Thbs1‐overexpressing engineered sEV provided a robust model for investigating TOE‐sEV function, key aspects such as the loading efficiency of recombinant Thbs1, safety profiles, and pharmacokinetics remained unexplored. Consequently, further research was essential to evaluate the feasibility and safety of directly employing engineered sEVs as a therapeutic strategy for wound healing. Moreover, whether pathways beyond PI3K/AKT signaling were involved in Thbs1‐overexpressing engineered sEV‐mediated M2 polarization of IL‐4‐induced BMDMs remained unknown.

Finally, the therapeutic efficacy and underlying mechanisms of the TOE strategy for diabetic ulcer healing remained to be fully elucidated. Future studies should explore the application of TOE in diabetic foot ulcer treatment through comprehensive animal models and clinical trials to validate its clinical potential.

## Funding

This work was supported by the Natural Science Foundation of China (Grant number 31970090) and Natural Science Foundation of Jilin (Grant number YDZJ202601ZYTS761).

## Ethics Statement

This study was approved by the Ethics Committee of the First Hospital of Jilin University (Approval No. JDYY20250651).

## Conflicts of Interest

The authors declare no conflicts of interest.

## Supporting information




**Supporting File 1**: advs73673‐sup‐0001‐SuppMat.docx.

**Supporting File 2**: advs73673‐sup‐0002‐Data.zip.

## Data Availability

The data that support the findings of this study are available on request from the corresponding author. Single‐cell data were uploaded to the GEO database (GSE314229).
