# Supplementary material for: Early Radial Extracorporeal Shockwave Stimulation on Proximal Tibial Circular Osteotomy Site Enhanced Heterotopic Skin Wound Healing via Small Extracellular Vesicles
Source: Adv Sci (Weinh). 2026 Jan 8;13(16):e17257. doi: 10.1002/advs.202517257 (PMC13042648; doi:10.1002/advs.202517257)
Supplement: Supplementary file 1 — Supporting File 1: advs73673‐sup‐0001‐SuppMat.docx. [file ADVS-13-e17257-s001.docx]

**Early Radial Extracorporeal Shockwave Stimulation on Proximal Tibial Circular Osteotomy Site Enhanced** **Heterotopic Skin Wound Healing via Small Extracellular Vesicles**

***Supplementary Material***

**Supplementary Fig.1. The effects of radial extracorporeal wave treatment (rESWT) with varying parameters on the viability of BMSC and MC3T3E1 osteoblast cells.**

**Supplementary Fig.2. Comparison of wound healing rates among TOE, TTT, rESWT alone, and TOE without bone flap preservation.**

**Supplementary Fig.3. Marker genes annotated for each indicated cell type in the scRNA-seq data.**

**Supplementary Fig.4. GO and KEGG analyses of differentially expressed genes in mesenchymal cells (MSCs) between (a)TO-2d-4h and TOE-2d-4h, (b)TO-2d-24h and TOE-2d-24h, (c)TO-4d-24h and TOE-4d-24h in the scRNA-seq data.**

**Supplementary Fig.5. Violin plots illustrating the distribution of exosomal markers (CD9, CD63, CD81, TSG101, Rab27a, Flot1, and Flot2) across distinct cell subpopulations.**

**Supplementary Fig.6. Overexpression of five candidate genes using varying plasmid amounts and their expression levels at 24 and 48 hours.**

**Supplementary Table.1. Quantitative analysis of wound healing rates across the five groups, corresponding to Figure 1d.**

**Supplementary Table.2. Quantitative analysis of wound healing rates across PBS, TO-sEV, and TOE-sEV groups, corresponding to Figure 4d.**

**Supplementary Table.3. Quantitative analysis of wound healing rates across PBS, sEV^OE-Vector^, and sEV^OE-Thbs1^ groups, corresponding to Figure 9b.**

**Supplementary Table.4. Quantitative analysis of wound healing rates across the five groups, corresponding to Supplementary Fig.2.**


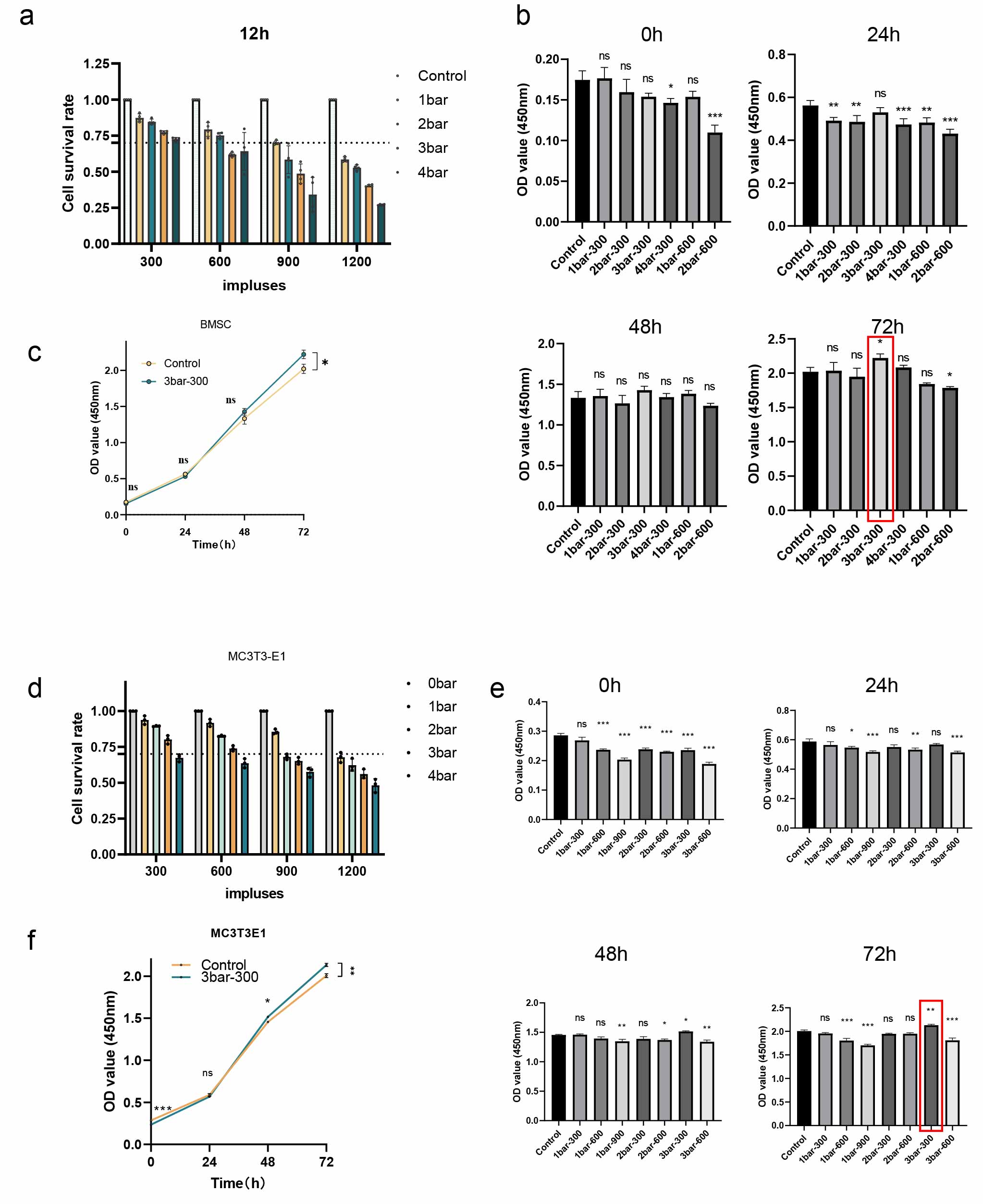


**Supplementary Fig.1. The effects of radial extracorporeal wave treatment (rESWT) with varying parameters on the viability of BMSC and MC3T3E1 osteoblast cells. a and d.** At the 12-hour time point, the viability of both BMSC and MC3T3E1 osteoblast cells exhibited a progressive decline with increasing shock wave pressure (1–4 bar) and stimulation frequency (300–1200 impulses), suggesting that higher energy doses may adversely affect cell survival. Based on these observations, only pressure-impulse combinations maintaining >70% viability at 12 hours were selected for subsequent experiments to ensure cellular fitness while retaining therapeutic potential (n=4). **b.** Immediately after treatment, the 4 bar-300 impulse and 2 bar-600 impulse groups showed significantly lower BMSC viability compared to controls (p<0.05). At 24 hours, all groups except 3 bar-300 impulses (which showed marginal reduction) exhibited significant decreases in BMSC viability. By 48 hours, no significant differences persisted between any treatment group and controls. Notably, at 72 hours, divergent effects emerged: the 3 bar-300 impulse group demonstrated significantly enhanced BMSC viability (p<0.05), while the 2 bar-600 impulse group remained suppressed (p<0.05), suggesting parameter-dependent temporal patterns in cellular recovery and proliferation. **c.** The line graph demonstrated a sustained increase in optical density (OD) values from 0 to 72 hours in the 3 bar-300 impulse group, with values significantly exceeding those of the control group by 72 hours (*p < 0.05). **e.** Immediately after treatment, all groups showed significantly lower MC3T3E1 osteoblast viability compared to controls (p<0.05), except for the 1 bar-300 impulse group. At 24 hours, all groups exhibited significant decreases in MC3T3E1 osteoblast viability, except for the 1 bar-300 impulse, 2 bar-300 impulse, and 3 bar-300 impulse groups. At 48 and 72 hours, the 3 bar-300 impulse group showed a significant increase in MC3T3E1 osteoblast viability compared to the control group **(f.)**. These results suggested that 3 bar-300 impulse stimulation not only enhances the proliferative activity of BMSC and MC3T3E1 osteoblast but also maintains this beneficial effect without observable cytotoxic accumulation throughout the 72-hour observation period. Data are presented as the mean ± SD. ns, not significant. p < 0.05 was regard as statistically significant.


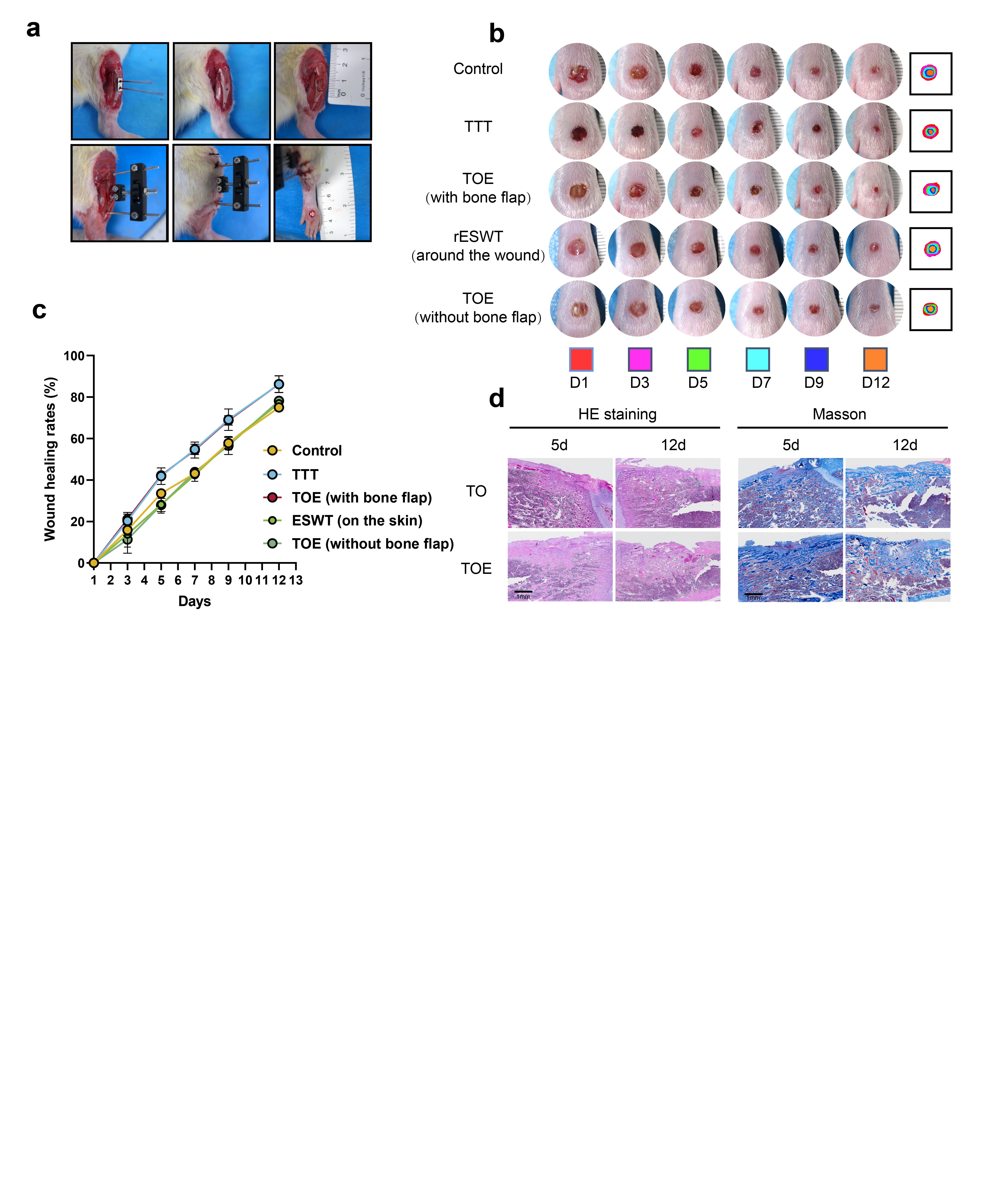


**Supplementary Fig.2. Comparison of wound healing rates among TOE, TTT, rESWT alone, and TOE without bone flap preservation.**

**a.** Surgical Procedure for Tibial Transverse Transport (TTT). In brief, eight-week-old rats were anesthetized with isoflurane, and the proximal of tibia was exposed. A drill guide was used to position and drill holes with 0.8 mm diameter Kirschner wires (K-wires) to outline the bone segment. After removing the guide, additional K-wires were employed around the initial holes to create an 8 mm × 4 mm rectangular bone segment, which was then mobilized. Screws were sequentially installed according to the external fixator assembly protocol. Finally, a 4 mm diameter full-thickness skin defect was created on the distal dorsal foot. Following a three-day postoperative latency period, the bone segment was transversely distracted outward at 0.25 mm every 12 hours for three days, achieving a total transport distance of 1.5 mm. After maintaining this position for three days, the bone segment was compressed inward at the same rate of 0.25 mm every 12 hours for three days.

**b.** Gross images of rat dorsal foot wounds on days 1, 3, 5, 7, 9 and 12 post-injury. The control group consisted of a skin wound healing model without any additional interventions. TTT underwent Tibial Transverse Transport Surgical Procedure. TOE (with bone flap preservation) underwent both tibial osteotomy and rESWT. TOE (without bone flap preservation) underwent both tibial osteotomy (removal of the bone flap) and rESWT. The rESWT (alone) group received rESWT applied directly to the wound periphery without any tibial surgical intervention.

**c.** Quantitative analysis of wound healing rates across the five experimental groups was performed using ImageJ software. (n=3)

**d.** Representative H&E and Masson-stained sections of the tibial osteotomy sites on postoperative days 5 and 12 between TO and TOE groups.


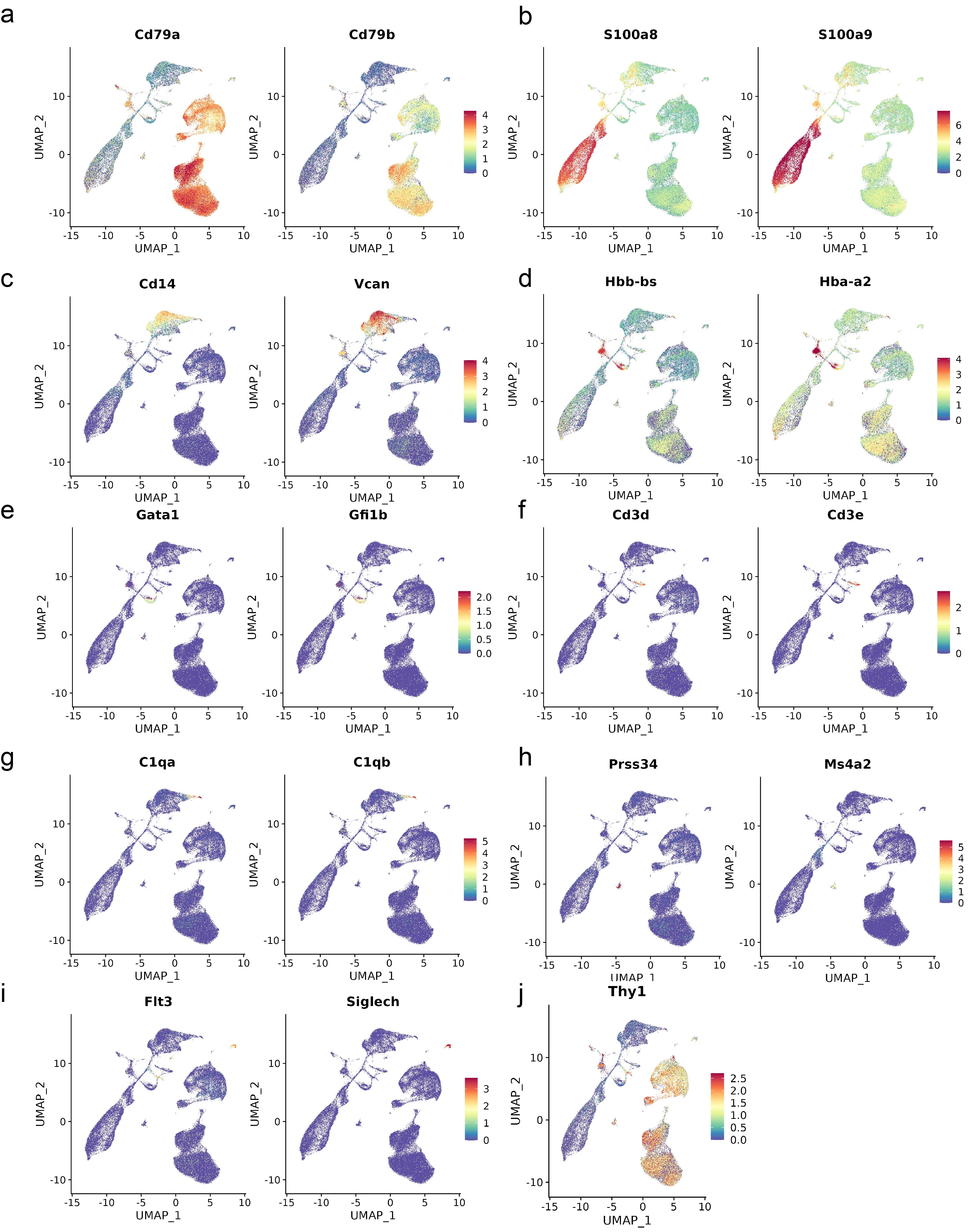


**Supplementary Fig.3. Marker genes annotated for each indicated cell type in the scRNA-seq data. a.** CD79a and CD79b for B cells. **b.** S100a8 and S100a9 for neutrophils. **c.** CD14 and Vcan for monocyte. **d.** Hbb-bs and Hba-a2 for erythrocyte. **e.** Gata1 and Gfi1b for hematopoietic stem and progenitor cells (HSPCs). **f.**CD3d and CD3e for T-cell and NK-cells. **g.** C1qa and C1qb for macrophages. **h.** Prss34 and Ms4a2 for mast cells and basophils. **i.** Flt3 and Siglech for DC cells. **j.** Thy1 for mesenchymal cells.


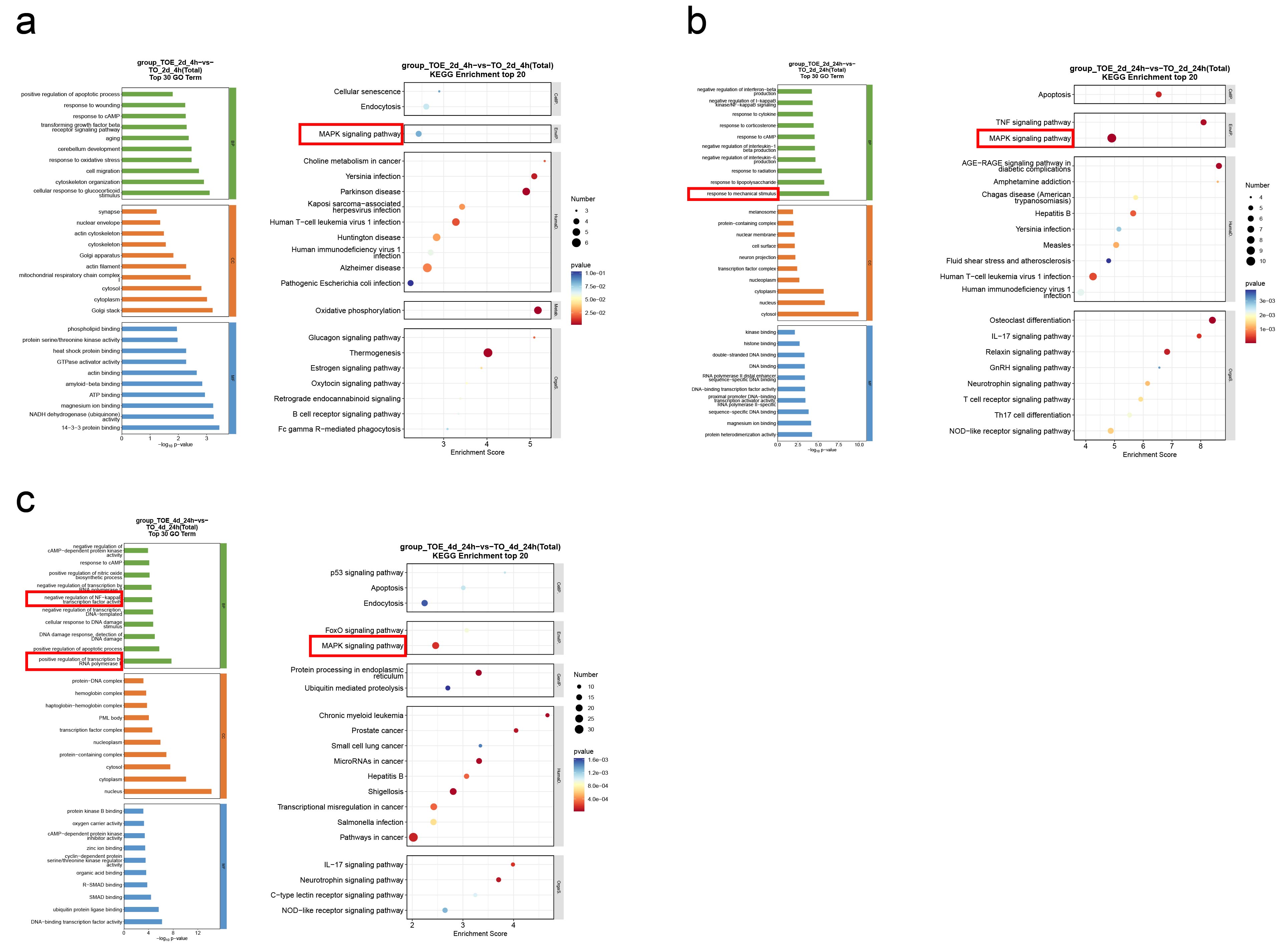


**Supplementary Fig.4.** GO and KEGG analyses of differentially expressed genes in mesenchymal cells (MSCs) between (**a**)TO-2d-4h and TOE-2d-4h, (**b**)TO-2d-24h and TOE-2d-24h, (**c**)TO-4d-24h and TOE-4d-24h in the scRNA-seq data. Across the three pairwise comparisons (TO-2d-4h vs. TOE-2d-4h, TO-2d-24h vs. TOE-2d-24h, and TO-4d-24h vs. TOE-4d-24h), KEGG pathway enrichment consistently highlighted the MAPK signaling pathway. In the TO-2d-24h vs. TOE-2d-24h comparison, GO biological process (BP) terms were primarily enriched in "response to mechanical stimulus." In the TO-4d-24h vs. TOE-4d-24h comparison, BP enrichments were predominantly associated with positive regulation of transcription by RNA polymerase II and negative regulation of NF-κB transcription factor activity. This suggested that shock wave stimulation on day 2 post-injury may primarily elicite a mechanical stimulus response in MSCs at the tibial osteotomy site, whereas stimulation on day 4 induce transcriptional modulation and inflammatory control activities.


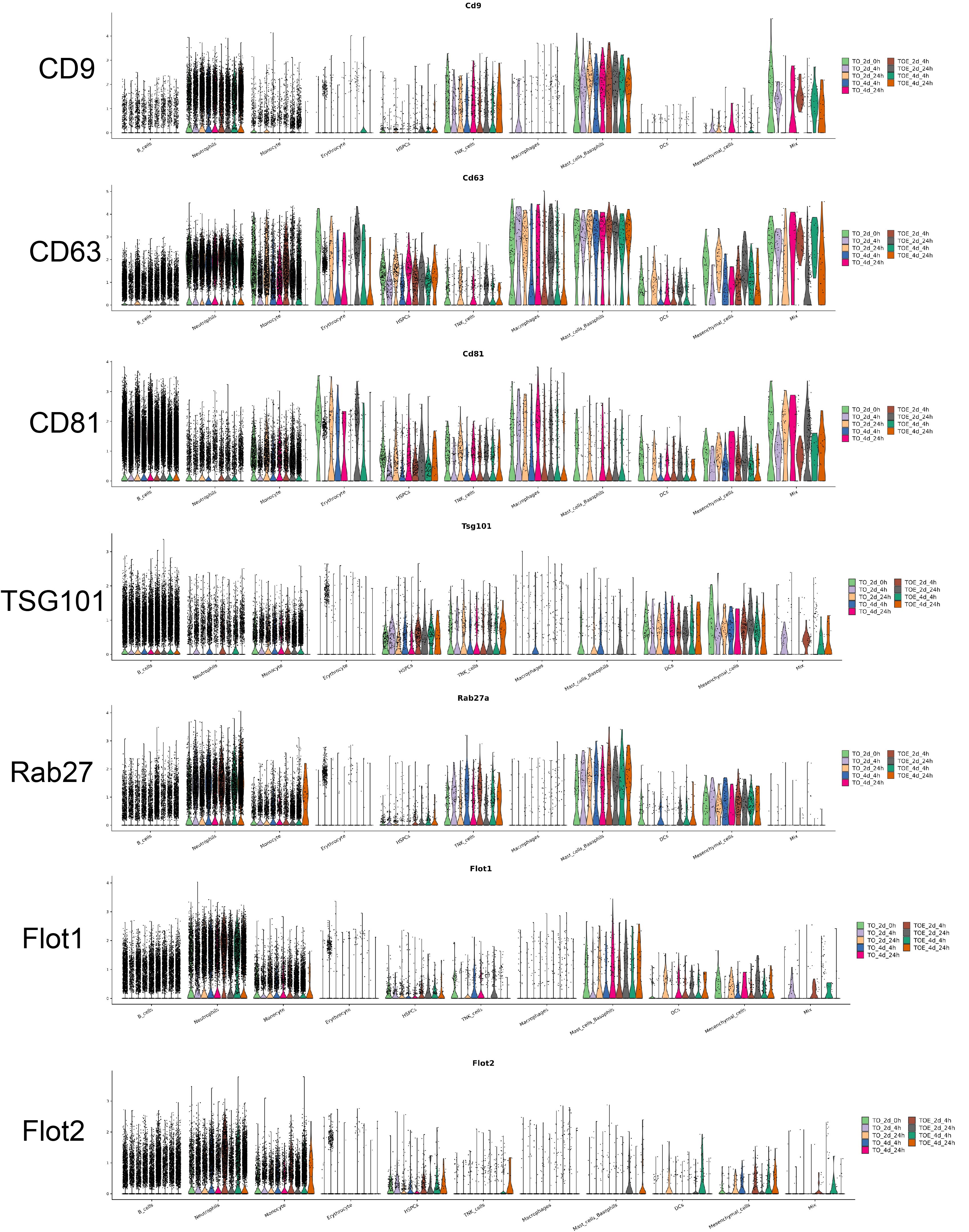


**Supplementary Fig.5.** Violin plots illustrating the distribution of exosomal markers (CD9, CD63, CD81, TSG101, Rab27a, Flot1, and Flot2) across distinct cell subpopulations.


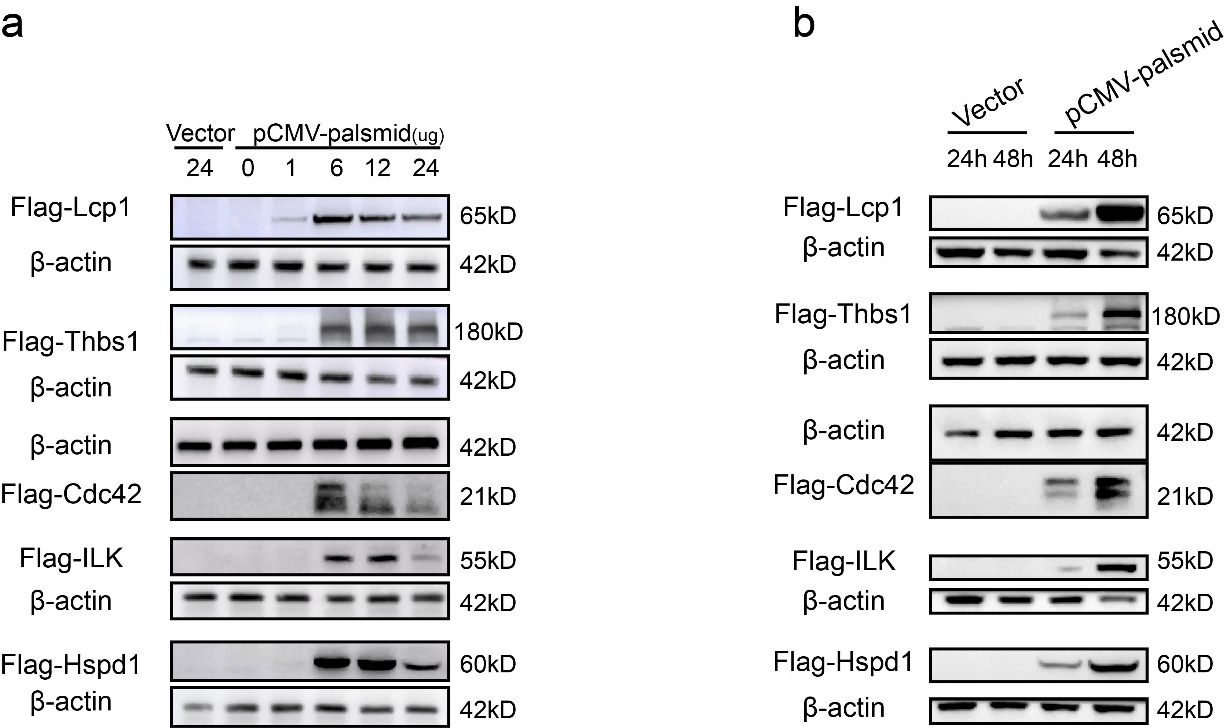


**Supplementary Fig.6. Overexpression of five candidate genes using varying plasmid amounts and their expression levels at 24 and 48 hours.**

**a.** Western blot analysis confirmed the expression of tagged proteins in 293T cells transfected with five overexpression plasmids (0, 1, 6, 12, and 24 μg/10-cm dish) and empty vector control (24 μg) at 24h post-transfection.

**b.** Western blot analysis detected the expression of tagged proteins in 293T cells at 24h and 48h post-transfection with overexpression plasmids (6 μg for Lcp1 and Cdc42, 12 μg each for Thbs1, ILK, and Hspd1). The expression levels of all plasmids were higher at 48 h compared to 24 h.

**Supplementary Table.1.** Quantitative analysis of wound healing rates across the five groups, corresponding to Figure 1d. Data are presented as the mean ± SD. p < 0.05 was regarded as statistically significant.

|  | **Wound healing rate** | **day1** | **day3*** | **day5** | **day7** | **Day9** | **day12** |
| --- | --- | --- | --- | --- | --- | --- | --- |
| Group 1 | **Control** | 0 | 21.70±3.61% | 43.34±4.71% | 51.12±5.21% | 58.67±4.12% | 64.87±4.24% |
| Group 2 | **TO** | 0 | 30.72±7.94% | 46.82±5.74% | 58.02±8.42% | 63.81±5.48% | 75.16±4.84% |
| Group 3 | **TE** | 0 | 22.24±9.09% | 42.45±5.17% | 55.76±6.20% | 61.25±6.54% | 69.22±6.04% |
| Group 4 | **TOE(contralateral)** | 0 | 30.85±14.24% | 54.06±6.29% | 72.18±4.41% | 80.9±3.64% | 91.11±1.90% |
| Group 5 | **TOE(ipsilateral)** | 0 | 32.01±4.45% | 64.43±5.18% | 69.72±3.70% | 85.66±4.12% | 94.78±2.30% |
| P (1vs 2) | | | 0.250 | 0.801 | 0.272 | 0.387 | 0.002 |
| P (1vs 3) | | | 1.000 | 0.998 | 0.647 | 0.889 | 0.391 |
| P (1vs 4) | | | 0.732 | 0.017 | <0.001 | <0.001 | <0.001 |
| P (1vs 5) | | | **0.013** | **<0.001** | <0.001 | <0.001 | <0.001 |
| P (2vs 3) | | | 0.613 | 0.639 | 0.961 | 0.893 | 0.131 |
| P (2vs 4) | | | 1.000 | 0.178 | 0.002 | <0.001 | <0.001 |
| P (2vs 5) | | | 1.000 | **<0.001** | 0.015 | <0.001 | <0.001 |
| P (3vs4) | | | 0.878 | 0.009 | <0.001 | <0.001 | <0.001 |
| P (3vs5) | | | 0.303 | **<0.001** | 0.003 | <0.001 | <0.001 |
| P (4vs5) | | | 1.000 | **0.022** | 0.947 | 0.462 | 0.557 |
| * On day 3, data from all groups conformed to a normal distribution but did not satisfy the assumption of homogeneity of variance; thus, ANOVA followed by Dunnett's T3 post-hoc test was employed. For the remaining days, ANOVA followed by Tukey's post-hoc test was used. | | | | | | | |

|  | **Wound healing rate (n=6)** | **day1** | **day3** | **day5** | **day7** | **day9** | **day12** |
| --- | --- | --- | --- | --- | --- | --- | --- |
| Group 1 | **PBS** | 0 | 20.12±3.29% | 37.99±1.97% | 54.58±6.43% | 67.06±6.00% | 77.73±3.48% |
| Group 2 | **TO-sEV** | 0 | 20.02±6.00% | 41.75±6.77% | 56.88±7.40% | 71.50±4.82% | 82.56±3.83% |
| Group 3 | **TOE-sEV** | 0 | 28.28±8.00% | 46.58±6.69% | 75.26±5.57% | 85.99±5.60% | 92.89±2.91% |
| P (1vs 2) | | | 0.078 | 0.494 | 0.817 | 0.365 | 0.067 |
| P (1vs 3) | | | 0.083 | 0.045 | <0.001 | <0.001 | <0.001 |
| P (2vs 3) | | | 0.078 | 0.322 | 0.001 | 0.001 | <0.001 |

**Supplementary Table.2.** Quantitative analysis of wound healing rates across PBS, TO-sEV, and TOE-sEV groups, corresponding to Figure 4d. Data are presented as the mean ± SD. For all days, ANOVA followed by Tukey's post-hoc test was used. p < 0.05 was regarded as statistically significant.

**Supplementary Table.3.** Quantitative analysis of wound healing rates across PBS, sEV^OE-Vector^, and sEV^OE-Thbs1^ groups, corresponding to Figure 9b. Data are presented as the mean ± SD. p < 0.05 was regarded as statistically significant.

|  | **Wound healing rate (n=6)** | **day1** | **day3*** | **day5** | **day7** | **day9*** | **day12*** |
| --- | --- | --- | --- | --- | --- | --- | --- |
| Group 1 | **PBS** | 0 | 24.11±6.99% | 37.47±5.62% | 49.16±6.92% | 63±9.67% | 73.21±9.26% |
| Group 2 | **sEV^OE-Vector^** | 0 | 25.51±5.42% | 43.10±2.71% | 54.05±4.36% | 69.86±6.32% | 78.85±3.12% |
| Group 3 | **sEV^OE-Thbs1^** | 0 | 27.27±2.86% | 49.86±2.69% | 64.56±4.92% | 81.89±2.33% | 88.85±2.92% |
| P (1vs 2) | | | 0.971 | 0.062 | 0.302 | 0.426 | 0.461 |
| P (1vs 3) | | | 0.682 | <0.001 | 0.001 | 0.011 | 0.021 |
| P (2vs 3) | | | 0.86 | 0.024 | 0.013 | 0.011 | 0.001 |
| * On day 3, 10, and 12, data from all groups conformed to a normal distribution but did not satisfy the assumption of homogeneity of variance; thus, ANOVA followed by Dunnett's T3 post-hoc test was employed. For the remaining days, ANOVA followed by Tukey's post-hoc test was used. | | | | | | | |

**Supplementary Table.4.** Quantitative analysis of wound healing rates across the five groups, corresponding to Supplementary Fig.2. Data are presented as the mean ± SD. For the all days, ANOVA followed by Tukey's post-hoc test was used. p < 0.05 was regarded as statistically significant.

|  | **Wound healing rate (n=3)** | **day1** | **day3** | **day5** | **day7** | **day9** | **day12** |
| --- | --- | --- | --- | --- | --- | --- | --- |
| Group 1 | **Control** | 0 | 15.85±2.58% | 33.51±1.49% | 43.15±1.87% | 57.78±2.77% | 74.98±1.04% |
| Group 2 | **TTT** | 0 | 20.27±4.15% | 41.86±4.02% | 54.75±2.53% | 69.08±5.14% | 86.19±4.04% |
| Group 3 | **TOE (with bone flap preservation)** | 0 | 21.06±2.50% | 42.20±1.81% | 54.45±3.87% | 68.72±2.27% | 86.22±4.06% |
| Group 4 | **rESWT alone** | 0 | 13.81±6.11% | 28.18±4.15% | 42.43±3.05% | 57.72±3.23% | 76.93±1.26% |
| Group 5 | **TOE (without bone flap preservation)** | 0 | 11.33±6.61% | 27.95±3.18% | 43.85±0.79% | 56.56±4.27% | 78.15±0.98% |
|  |  |  |  |  |  |  |  |
| P (1vs 2) | | | 0.779 | 0.052 | 0.002 | 0.024 | 0.003 |
| **P (1vs 3)** | | | 0.667 | 0.042 | 0.003 | 0.029 | 0.003 |
| P (1vs 4) | | | 0.982 | 0.296 | 0.997 | 1 | 0.896 |
| P (1vs 5) | | | 0.766 | 0.263 | 0.997 | 0.993 | 0.619 |
| **P (2vs 3)** | | | 1 | 1 | 1 | 1 | 1 |
| P (2vs 4) | | | 0.486 | 0.002 | 0.001 | 0.024 | 0.012 |
| P (2vs 5) | | | 0.215 | 0.002 | 0.003 | 0.013 | 0.029 |
| **P (3vs 4)** | | | 0.384 | 0.002 | 0.002 | 0.028 | 0.012 |
| **P (3vs 5)** | | | 0.16 | 0.002 | 0.004 | 0.016 | 0.028 |
| P (4vs 5) | | | 0.964 | 1 | 0.961 | 0.995 | 0.979 |
