# Supplementary material for: Early Radial Extracorporeal Shockwave Stimulation on Proximal Tibial Circular Osteotomy Site Enhanced Heterotopic Skin Wound Healing via Small Extracellular Vesicles
Source: Adv Sci (Weinh). 2026 Jan 8;13(16):e17257. doi: 10.1002/advs.202517257 (PMC13042648; doi:10.1002/advs.202517257)
Supplement: Supplementary file 2 — Supporting File 2: advs73673‐sup‐0002‐Data.zip. [file ADVS-13-e17257-s002.zip › advs73673-sup-0002-Data/IHC and IF data.pdf]

Figure 1 e and f

Con
TO
TE
TOE Contra-lateral
TOE ipsi-lateral

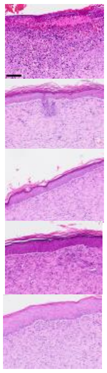

Epidermal thickness(μm)

#1	#2	#3
40.46	49.68	39.22
38.08	49.60	40.38
47.30	41.54	43.22
66.92	81.90	58.84
99.22	74.98	71.52

Con
TO
TE
TOE Contra-lateral
TOE ipsi-lateral

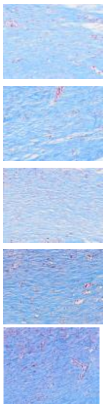

Collagen volume fraction(%)

#1	#2	#3
26.61	28.63	38.21
20.04	40.20	32.86
32.33	35.11	42.75
61.26	51.55	50.72
59.29	67.64	54.20

Con
TO
TE
TOE Contra-lateral
TOE ipsi-lateral

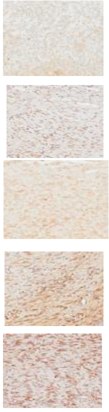

Col 1 area(%)

#1	#2	#3
6.36	7.44	8.64
7.81	7.10	8.86
5.88	8.50	8.35
16.50	17.04	11.99
12.94	14.24	18.10

Con
TO
TE
TOE Contra-lateral
TOE ipsi-lateral

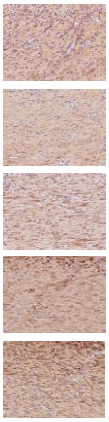

Col 3 area(%)

#1	#2	#3
20.02	25.19	27.81
20.49	21.62	26.69
21.38	25.29	19.87
33.69	30.04	29.26
32.51	33.84	36.47

Con
TO
TE
TOE Contra-lateral
TOE ipsi-lateral

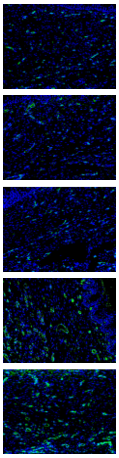

CD31+ vessels

#1	#2	#3
26	17	28
28	15	20
15	26	24
52	45	40
40	70	63

Figure 1 j and k

Epidermal thickness(μm)

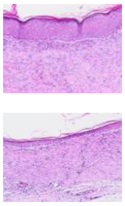

	#1	#2	#3
TOE	120	87.34	79.38
TOE+ GW4869	23.43	53.34	45.30

Col 1 area(%)

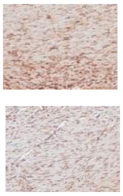

	#1	#2	#3
TOE	19.06	22.26	18.28
TOE+ GW4869	10.54	10.44	11.41

CD31+ vessels

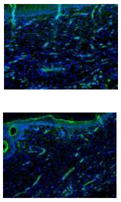

	#1	#2	#3
TOE	37	42	32
TOE+ GW4869	17	29	24

Collagen volume fraction(%)

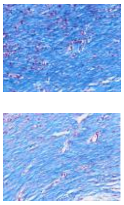

	#1	#2	#3
TOE	42.18	40.11	48.90
TOE+ GW4869	25.90	22.48	17.34

Col 3 area(%)

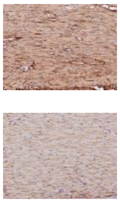

	#1	#2	#3
TOE	44.59	46.05	31.91
TOE+ GW4869	27.19	25.14	23.14

Figure 3 h and i

Length of wound (μm)

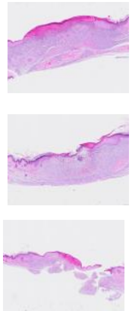

	#1	#2	#3
PBS	629.55	332.51	925.57
TO-sEV	1228.78	1155.64	1277.89
TOE-sEV	1314.81	1235.86	1489.36

Collagen volume fraction(%)

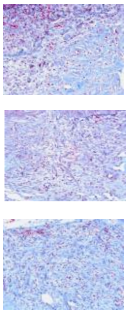

	#1	#2	#3
PBS	10.28	9.22	10.89
TO-sEV	10.85	12.23	8.98
TOE-sEV	8.49	10.54	13.37

Col 1 area(%)

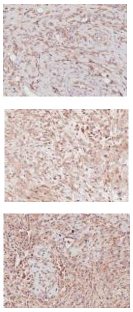

	#1	#2	#3
PBS	11.72	18.38	19.29
TO-sEV	26.33	24.78	25.47
TOE-sEV	41.79	32.43	34.85

Col 3 area(%)

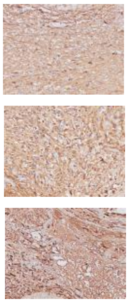

	#1	#2	#3
PBS	24.07	14.75	27.73
TO-sEV	38.05	35.34	29.50
TOE-sEV	42.37	56.62	41.23

PBS TO-sEV TOE-sEV

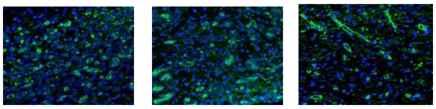

Figure 9 c-h

Epidermal thickness(μm)

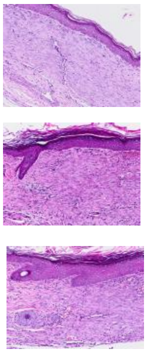

	#1	#2	#3
PBS	71.54	67.83	51.70
$sEV^{OE-Vector}$	64.66	76.73	72.82
$sEV^{OE-Thbs1}$	128.95	159.11	104.73

Collagen volume fraction(%)

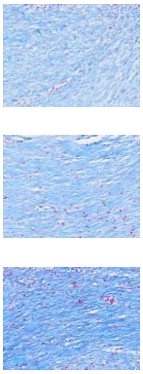

	#1	#2	#3
PBS	13.41	17.35	24.76
$sEV^{OE-Vector}$	18.34	20.57	38.22
$sEV^{OE-Thbs1}$	24.76	26.53	41.16

Col 1 area(%)

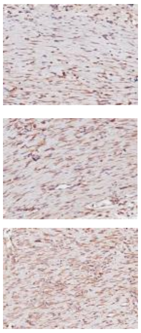

	#1	#2	#3
PBS	10.40	9.20	14.27
$sEV^{OE-Vector}$	11.53	13.28	15.56
$sEV^{OE-Thbs1}$	22.23	21.58	22.59

Col 3 area(%)

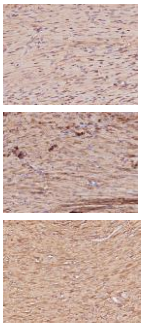

	#1	#2	#3
PBS	29.59	27.48	34.24
$sEV^{OE-Vector}$	31.52	30.58	34.51
$sEV^{OE-Thbs1}$	38.51	39.52	41.95

CD31+ vessels

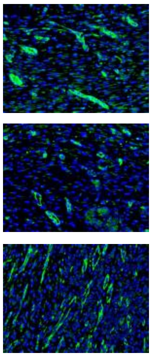

	#1	#2	#3
PBS	15	18	17
$sEV^{OE-Vector}$	16	19	18
$sEV^{OE-Thbs1}$	32	34	28

Figure 1n

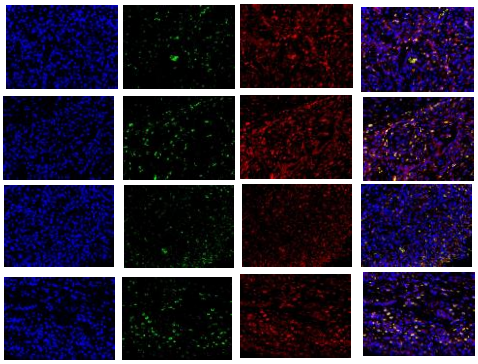

Figure 1 o

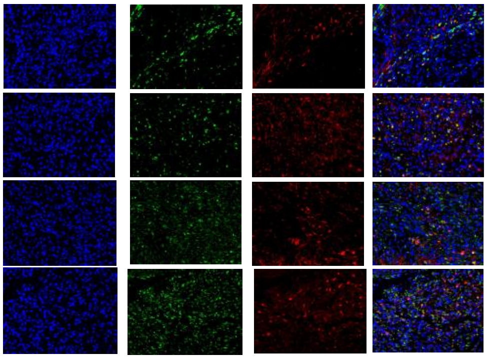

Figure 1 p

M1	Control	TO	TOE	TOE+G W4869
#1	43	44	25	35
#2	35	38	16	30
#3	38	32	22	47

M2	Cont rol	TO	TOE	TOE+G W4869
#1	10	10	14	13
#2	6	8	17	8
#3	9	11	15	9

Figure 3 j and l

PBS LPS LPS+TO-sEV LPS+TOE-sEV

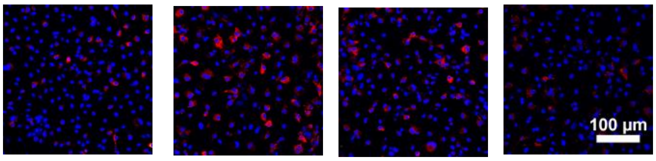

M1	PBS	LPS	LPS+TO-sEV	LPS+TOE-sEV
#1	11.26	22.91	15.79	11.46
#2	8.678	21.9	19.78	12.5
#3	9.877	18.22	16.44	13.54

Figure 3 k and m

PBS IL4 IL4+TO-sEV IL4+TOE-sEV

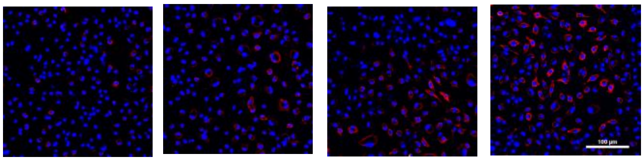

M2	PBS	IL4	IL4+TO-sEV	IL4+TOE-sEV
#1	7.32	15.47	18.18	26.34
#2	7.23	13.71	13.09	25.58
#3	6.87	10.61	17.22	24.46

Figure 4 j and l

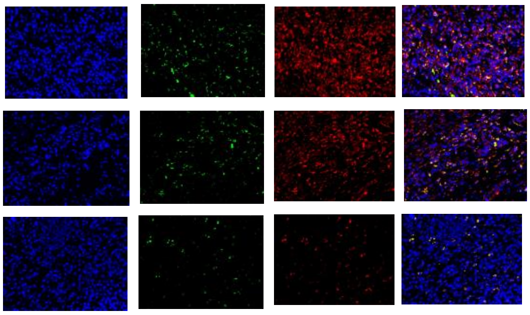

M1	PBS	TO-sEV	TOE-sEV
#1	42	19	14
#2	51	29	7
#3	38	25	9

Figure 4 k and m

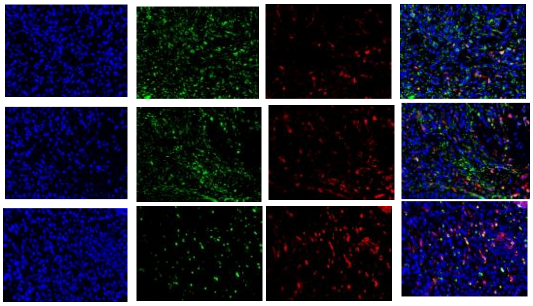

M2	PBS	TO-sEV	TOE-sEV
#1	13	10	28
#2	4	14	22
#3	10	17	34

Figure 8 g and h

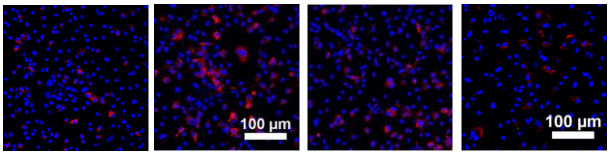

M1	PBS	LPS	LPS+sEVO E-Vector	LPS+sEVO E-Vector
#1	11.27	33.2	19.41	13.21
#2	16.89	29.78	30.67	14.14
#3	15.73	25.53	24.23	14.88

Figure 8 i and j

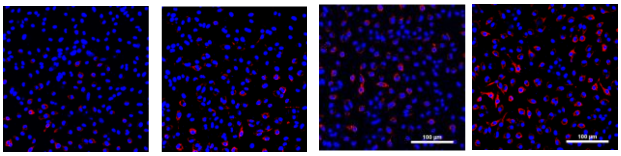

M2	PBS	IL4	IL4+sEVO E-Vector	IL4+sEVOE -Vector
#1	17.22	21.59	29.84	32.77
#2	10.61	22.04	27.93	39.84
#3	15.18	28.23	24.52	38.51

Figure 9 i and j

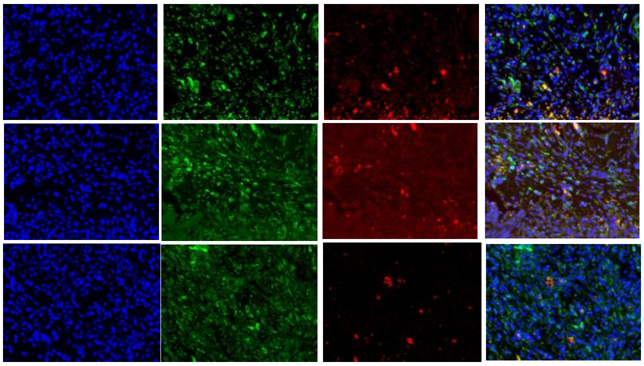

M1	PBS	sEVOE-Vector	sEVOE-Thbs1
#1	19	13	5
#2	16	14	9
#3	14	12	7

Figure 9 k and l

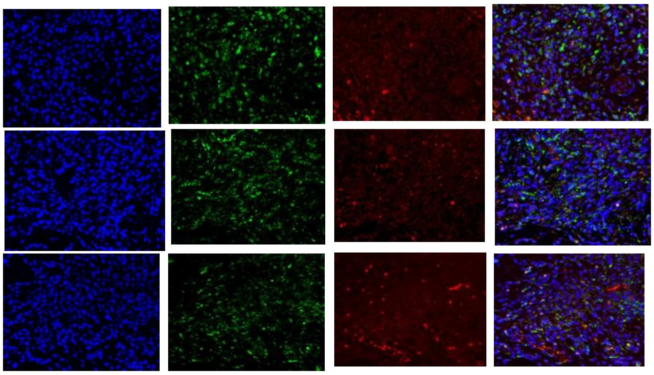

M2	PBS	sEVOE-Vector	sEVOE-Thbs1
#1	7	9	18
#2	8	10	19
#3	9	15	16
