# Supplementary material for: Early Radial Extracorporeal Shockwave Stimulation on Proximal Tibial Circular Osteotomy Site Enhanced Heterotopic Skin Wound Healing via Small Extracellular Vesicles
Source: Adv Sci (Weinh). 2026 Jan 8;13(16):e17257. doi: 10.1002/advs.202517257 (PMC13042648; doi:10.1002/advs.202517257)
Supplement: Supplementary file 2 — Supporting File 2: advs73673‐sup‐0002‐Data.zip. [file ADVS-13-e17257-s002.zip › advs73673-sup-0002-Data/Other raw data.pdf]

Figure 2e

	TO	TOE	TOE + GW4869
#1	1.53e+010	2.21e+010	1.61e+010
#2	1.4e+010	2.11e+010	1.77e+010
#3	1.76e+010	2.52e+010	1.9e+010

Figure 2j

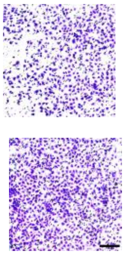

#1	#2	#3
410	451	406
543	599	736

Figure 2f

24h	PBS				TO-sEV				TOE-sEV			
#1	1	1	1	1	1.079861	1.063657	1.024884	1.015625	1.133681	1.115741	1.128472	1.152778
#2	1	1	1	1	1.081676	1.06362	1.095782	1.133023	1.184934	1.160671	1.151079	1.138101
#3	1	1	1	1	1.15643	1.16244	1.14496	1.12913	1.20994	1.2285	1.19847	1.19902

48h	PBS				TO-sEV				TOE-sEV			
#1	1	1	1	1	1.137064	1.143097	1.146448	1.180295	1.232574	1.211126	1.215483	1.201408
#2	1	1	1	1	1.059782	1.155086	1.131866	1.173107	1.220239	1.255588	1.219199	1.229943
#3	1	1	1	1	1.249833	1.265844	1.226484	1.21481	1.29553	1.363576	1.333889	1.334556

Figure 2h

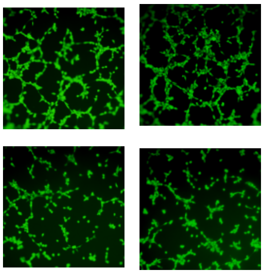

	TO-sEV	TOE-sEV	TO non-sEV	TOE non-sEV
#1	25	36	5	7
#2	26	34	12	14
#3	28	43	13	15

Figure 2l

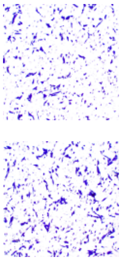

**#1**	**#2**	**#3**
145	147	155
257	272	245

Figure 2i

12h

24h

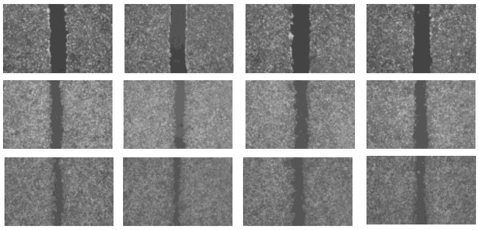

**12h**	**TO-sEV**	**TOE-sEV**	**TO non-sEV**	**TOE non-sEV**
#1	44.45113	50.77497	38.36332	34.8321
#2	43.94111	52.40535	35.91287	41.54029
#3	46.21606	49.59005	34.65683	45.18369

**24h**	**TO-sEV**	**TOE-sEV**	**TO non-sEV**	**TOE non-sEV**
#1	62.31579	69.5465	55.42658	53.21571
#2	59.79615	67.94762	50.41559	56.52691
#3	61.14962	68.08029	49.0641	56.11802

Figure 2k

12h

24h

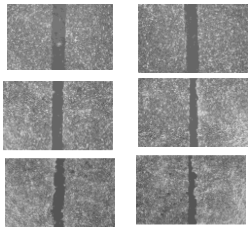

	12h		24h	
	**TO-sEV**	**TOE-sEV**	**TO-sEV**	**TOE-sEV**
#1	26.23778	35.85257	35.9508	43.0752
#2	32.47863	28.22037	41.46773	40.78478
#3	17.87302	36.84637	31.74603	68.50426
#4	24.00121	30.39178	30.75061	39.02625
#5	30.76384	32.20398	32.30554	51.55431
#6	23.67116	40.29516	24.73423	55.18126

Figure 3b-d

M1	PBS	LPS	LPS+ TO- sEV	LPS+ TOE- sEV
#1	4.53	92.4	92.2	39.6
#2	2.73	93.1	80.3	53.4
#3	2	89.3	85	45.7

Figure 3e-g

M2	PBS	IL4	IL4+ TO- sEV	IL4+ TOE- sEV
#1	2.16	23.7	38.7	55.2
#2	1.91	22.9	35.4	54.5
#3	1.68	24.9	29.9	54.2

Figure 5i

	BMSC-sEV	SW-BMSC-sEV
#1	4.87e+009	8.67e+009
#2	6.71e+009	9.25e+009
#3	3.95e+009	7.51e+009

Figure 7l

sEV$^{OE-Vector}$ sEV$^{OE-Thbs1}$

12h

48h

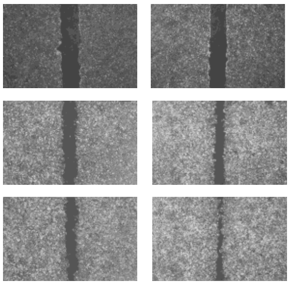

	12h		24h	
	sEV^{OE-} Vector	sEV^{OE-} Thbs1	sEV^{OE-} Vector	sEV^{OE-} Thbs1
#1	25.41798	33.27068	40.9983	49.40476
#2	25.25568	37.86834	54.94318	71.06583
#3	25.66675	42.86822	42.48746	60.54264
#4	27.60894	46.18321	47.30505	69.13168
#5	26.24274	40.8897	52.00129	67.58074
#6	23.83204	46.59166	45.32814	78.88815

Figure 7j

sEV$^{OE-Vector}$

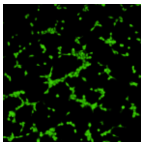

sEV$^{OE-Thbs1}$

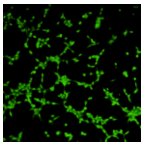

#1	#2	#3
8	15	8
10	18	10

Figure 7i

	PBS				sEV$^{OE-Vector}$				sEV$^{OE-Thbs1}$			
#1	1	1	1	1	1.100814	1.141719	1.129412	1.18371	1.271674	1.229683	1.228959	1.220271
#2	1	1	1	1	1.250327	1.253809	1.236744	1.303265	1.32451	1.345407	1.362473	1.333217
#3	1	1	1	1	1.100814	1.141719	1.129412	1.18371	1.271674	1.229683	1.228959	1.220271

Figure 8 b and e

	PBS	IL4	PBS+ sEVOE-Vector	PBS+ sEV OE-Thbs1
#1	2.43	92.7	85.4	34.6
#2	2.22	84.7	82.9	28.8
#3	2.65	86.2	80.2	36.9

Figure 8 c and f

	PBS	IL4	IL4+ sEVOE-Vector	IL4+ sEV OE-Thbs1
#1	1.77	22.3	27.5	46.6
#2	1.56	24.3	30.4	49.6
#3	0.08	23.8	32.1	39.4
