# Supplementary material for: Early Radial Extracorporeal Shockwave Stimulation on Proximal Tibial Circular Osteotomy Site Enhanced Heterotopic Skin Wound Healing via Small Extracellular Vesicles
Source: Adv Sci (Weinh). 2026 Jan 8;13(16):e17257. doi: 10.1002/advs.202517257 (PMC13042648; doi:10.1002/advs.202517257)

Figure 1. l

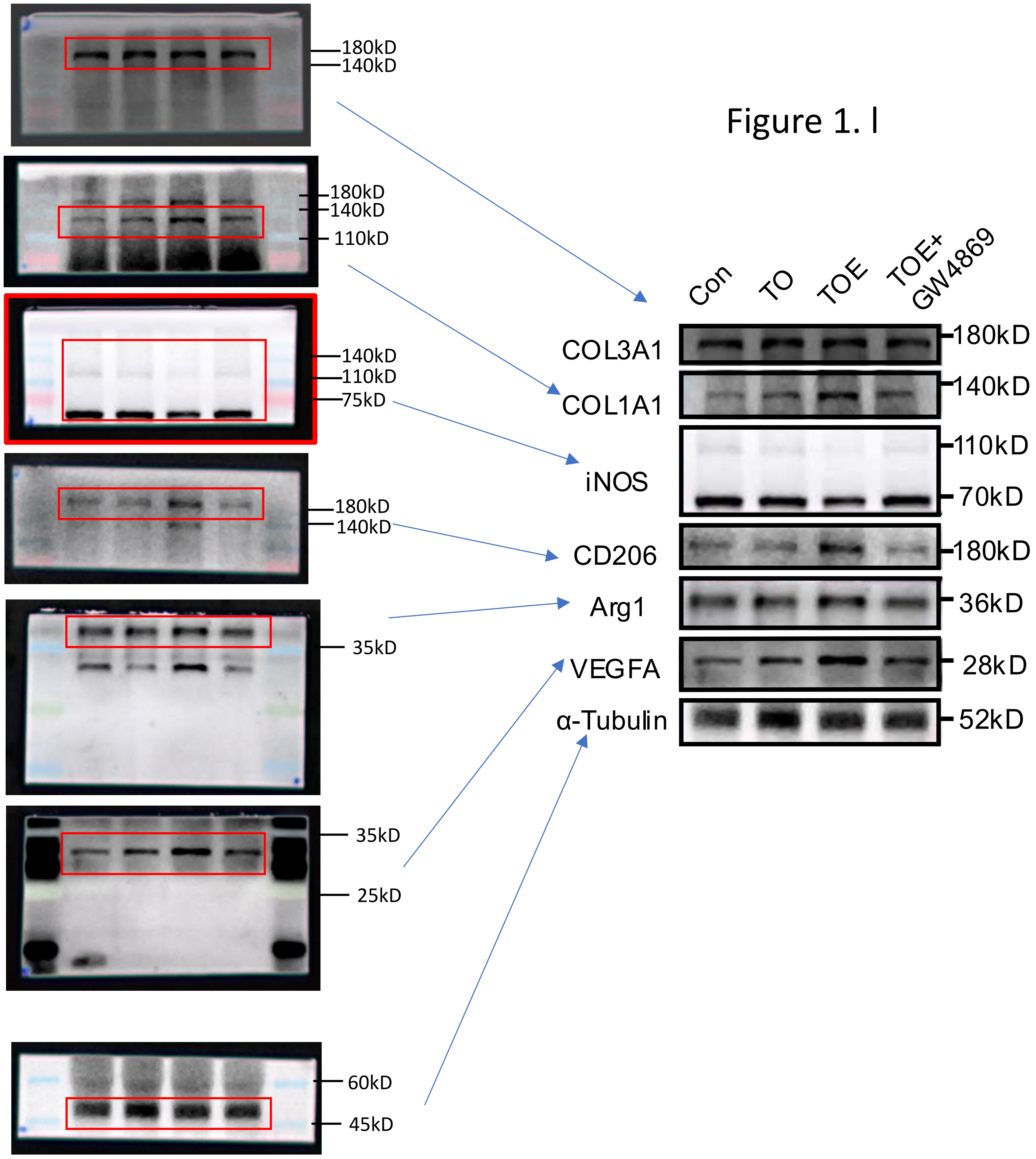
180kD
140kD
180kD
140kD
110kD
140kD
110kD
75kD
180kD
140kD
35kD
35kD
25kD
60kD
45kD
Con
TO
TOE
TOE+ GW4869
COL3A1
COL1A1
iNOS
CD206
Arg1
VEGFA
α-Tubulin
180kD
140kD
110kD
70kD
180kD
36kD
28kD
52kD

Figure 2.c

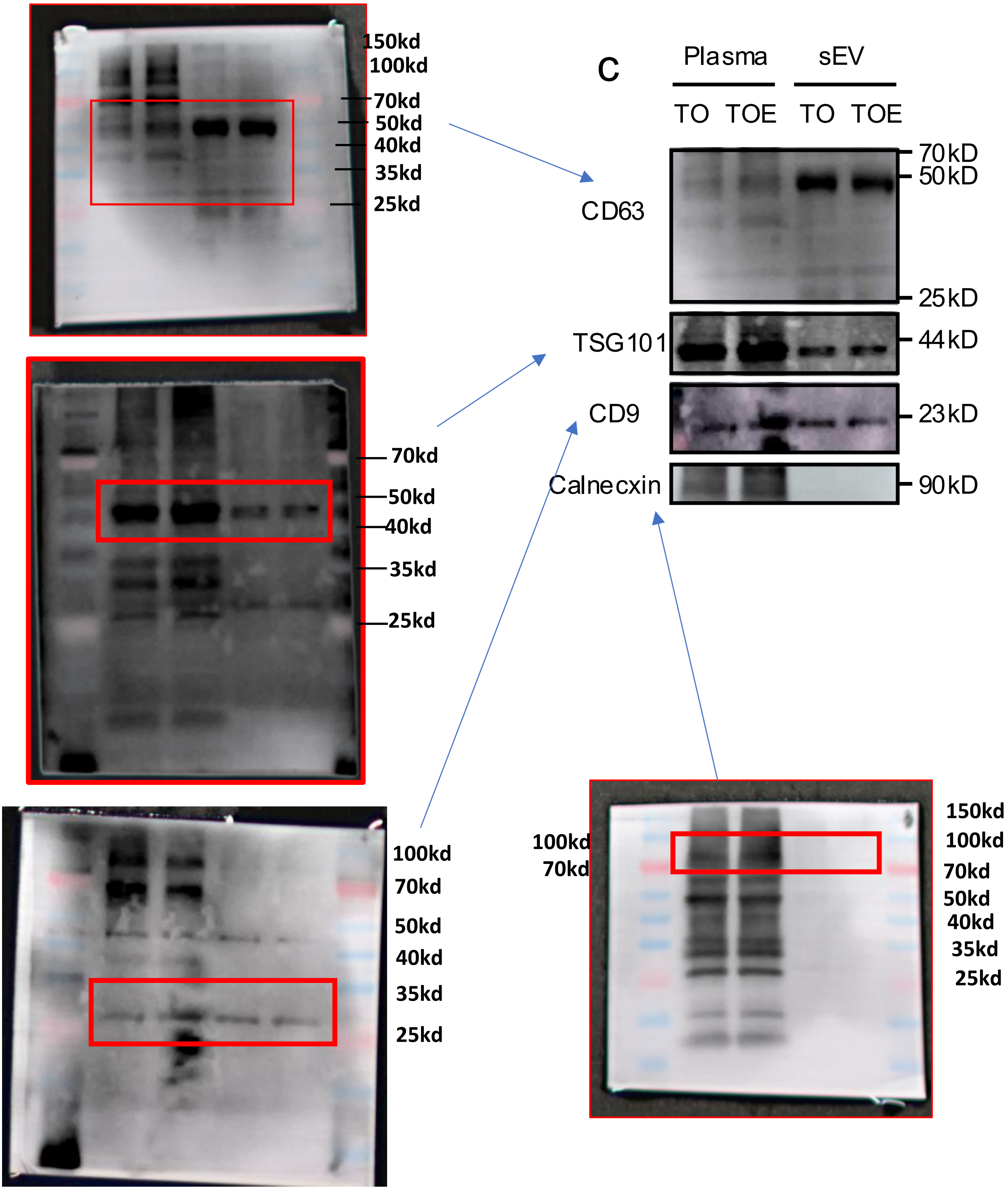

150kd
100kd
70kd
50kd
40kd
35kd
25kd
C
Plasma
sEV
TO
TOE
TO
TOE
CD63
70kD
50kD
25kD
TSG101
44kD
CD9
23kD
Calnecxin
90kD
70kd
50kd
40kd
35kd
25kd
100kd
70kd
50kd
40kd
35kd
25kd
100kd
70kd
150kd
100kd
70kd
50kd
40kd
35kd
25kd

Figure 3.h&i

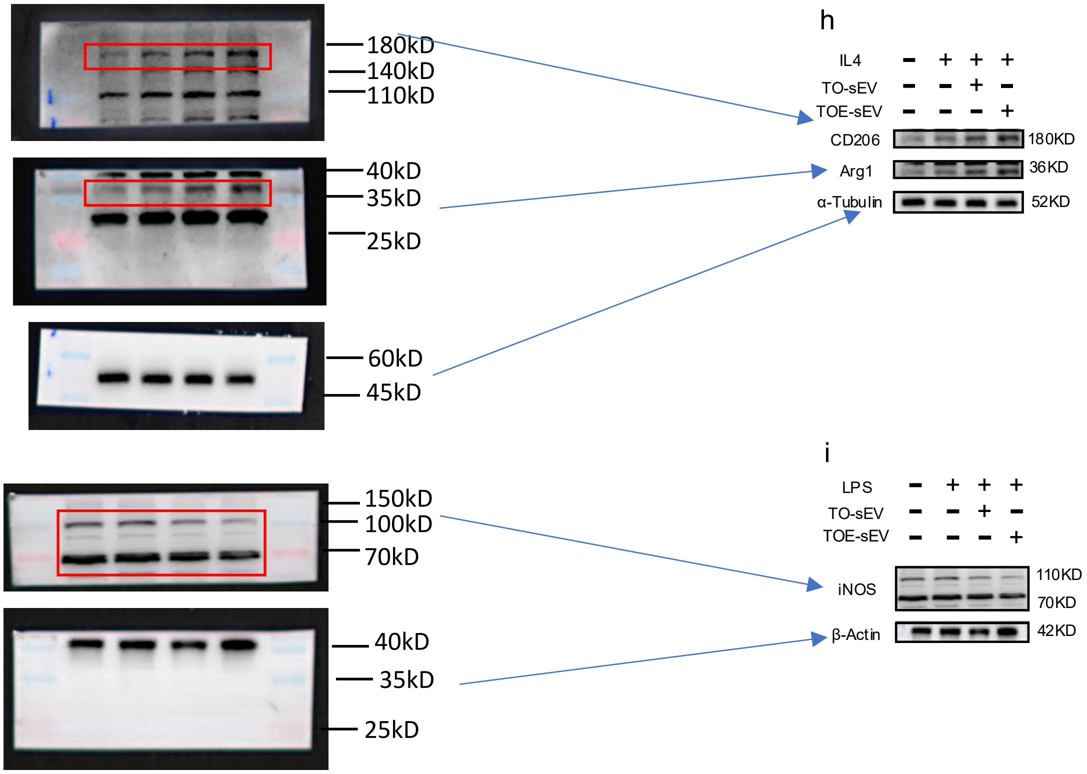
180kD
140kD
110kD
40kD
35kD
25kD
60kD
45kD
150kD
100kD
70kD
40kD
35kD
25kD
h
IL4 − + + +
TO-sEV − − + −
TOE-sEV − − − +
CD206
180KD
Arg1
36KD
α-Tubulin
52KD
i
LPS − + + +
TO-sEV − − + −
TOE-sEV − − − +
iNOS
110KD
70KD
β-Actin
42KD

Figure 4.e

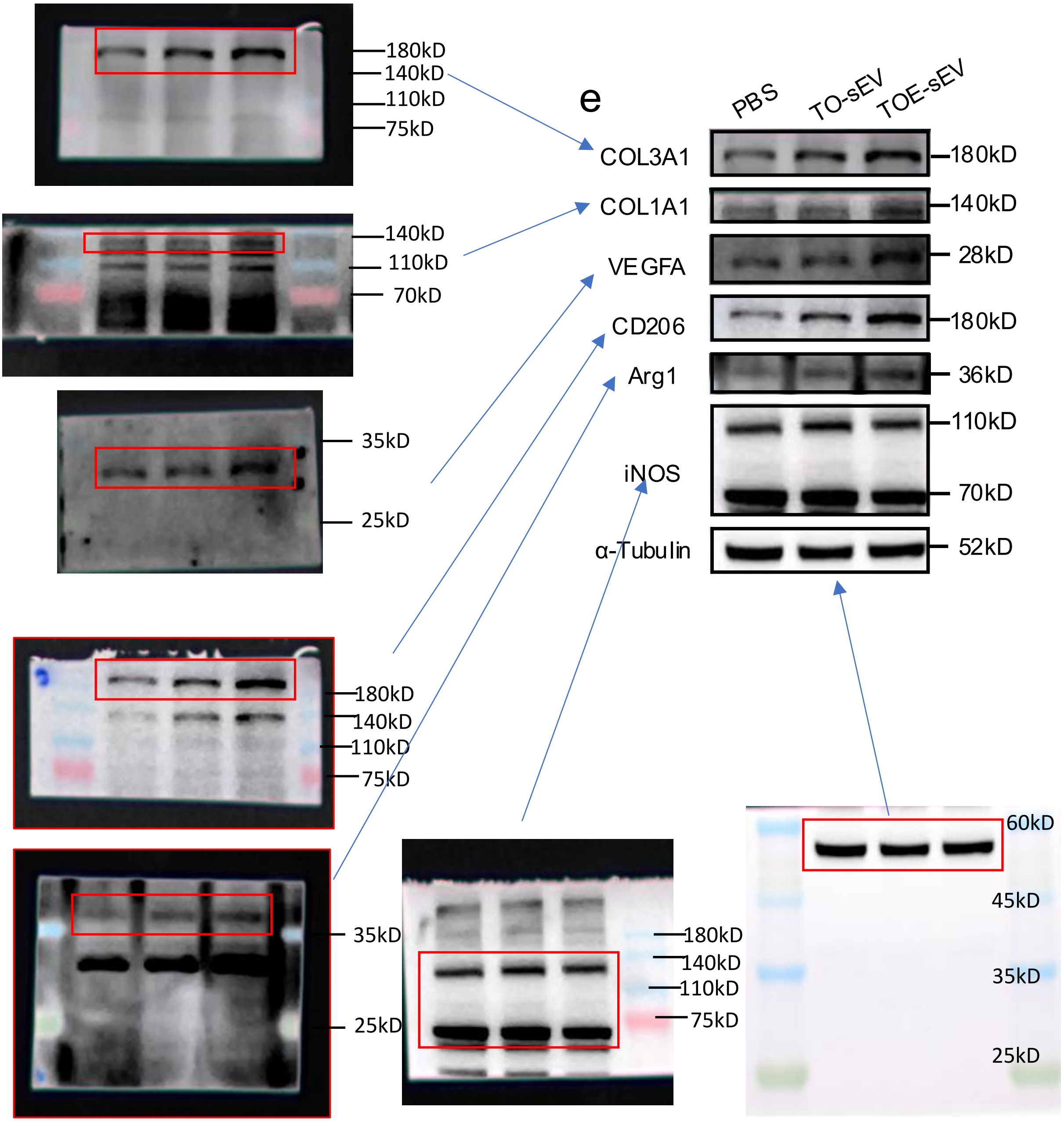
e
PBS
TO-sEV
TOE-sEV
COL3A1
COL1A1
VEGFA
CD206
Arg1
iNOS
α-Tubulin
180kD
140kD
28kD
180kD
36kD
110kD
70kD
52kD
180kD
140kD
110kD
75kD
140kD
110kD
70kD
35kD
25kD
180kD
140kD
110kD
75kD
35kD
25kD
180kD
140kD
110kD
75kD
60kD
45kD
35kD
25kD

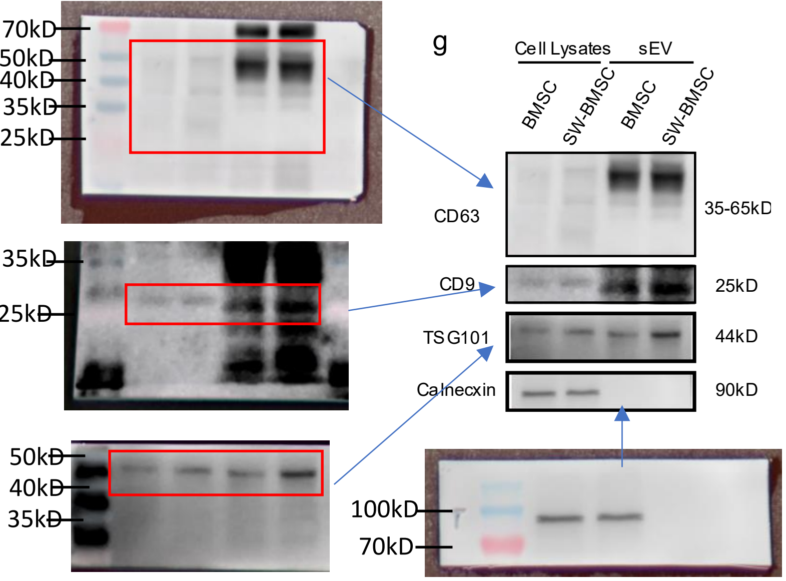

Figure 5.g

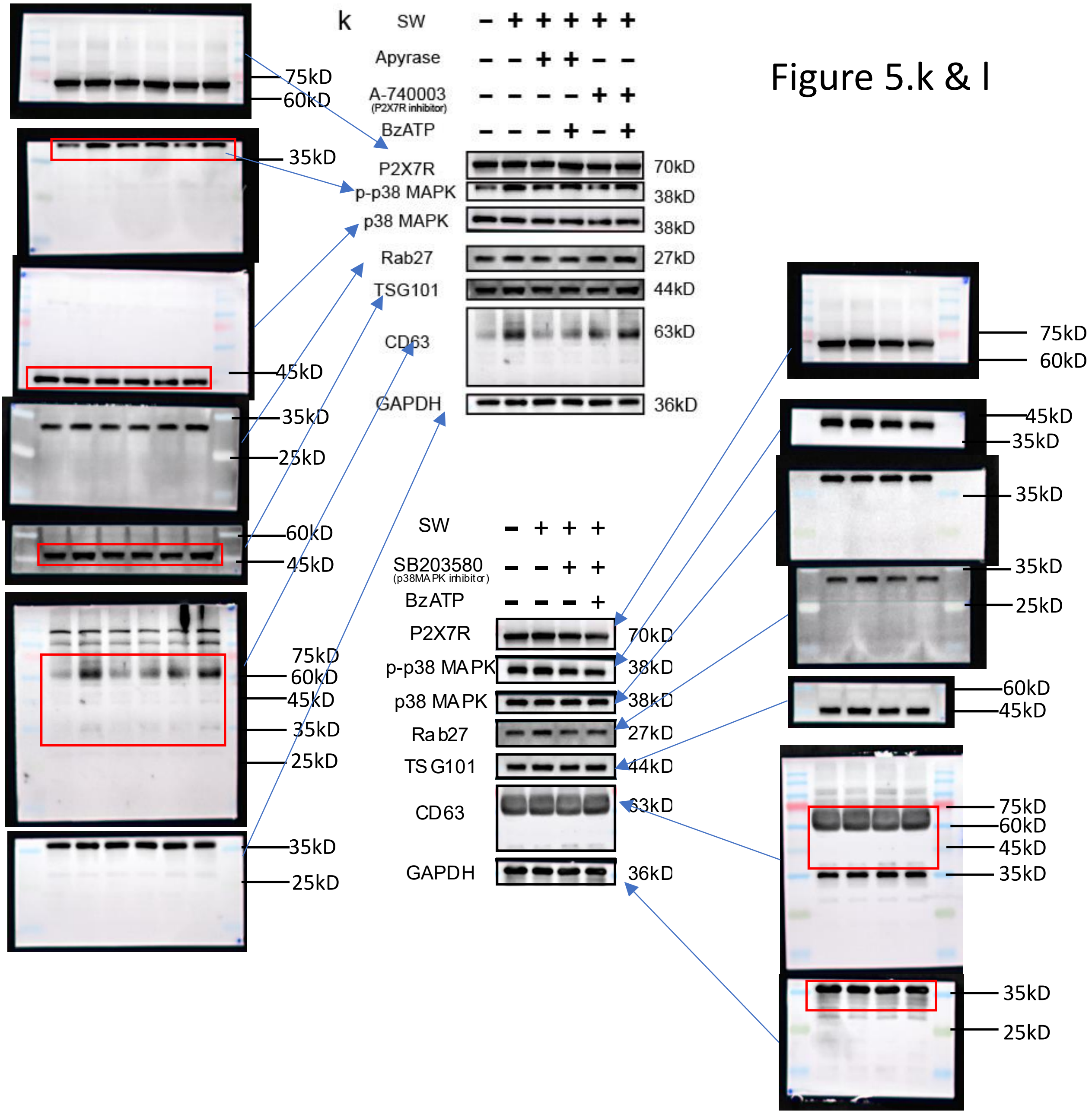

Figure 5.k & l

Figure 6.h

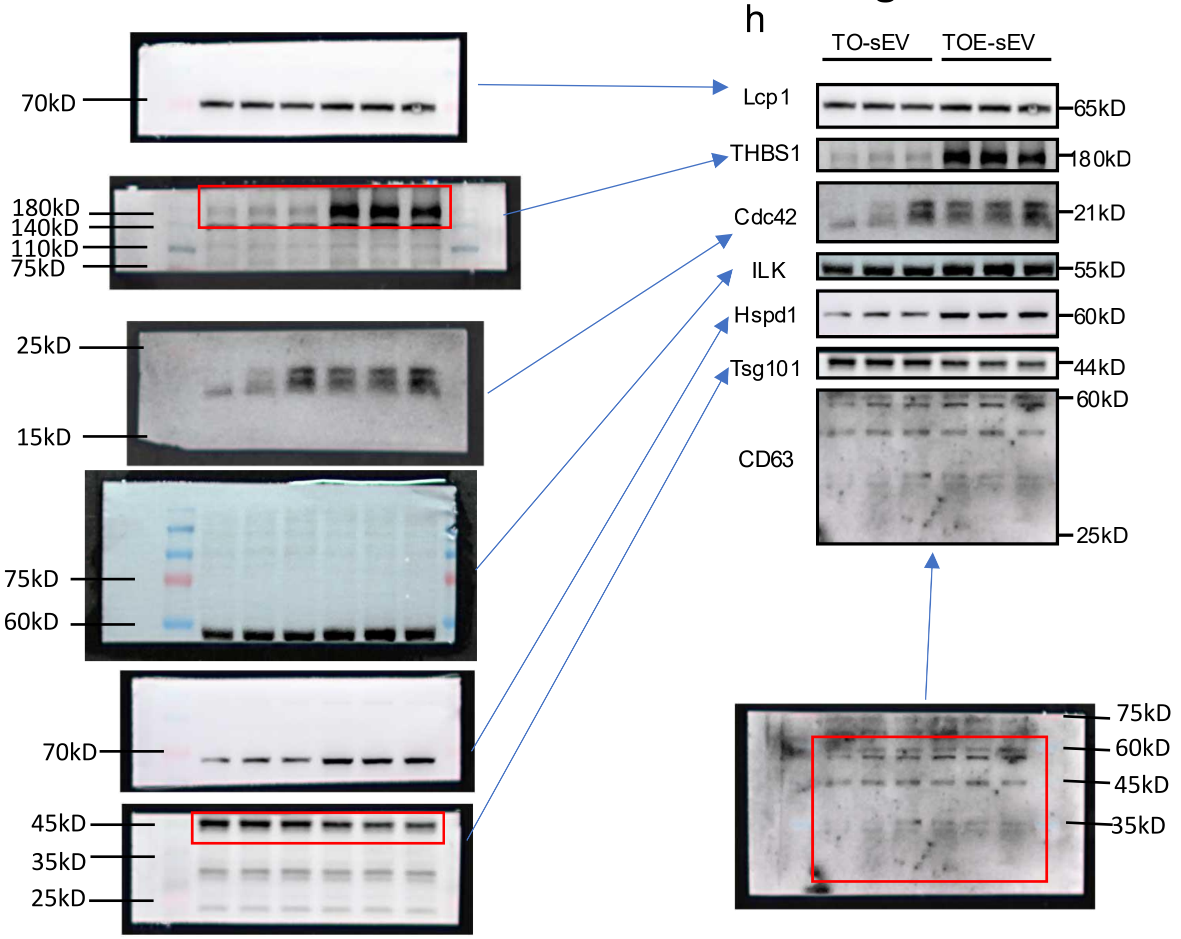

Figure 7.b

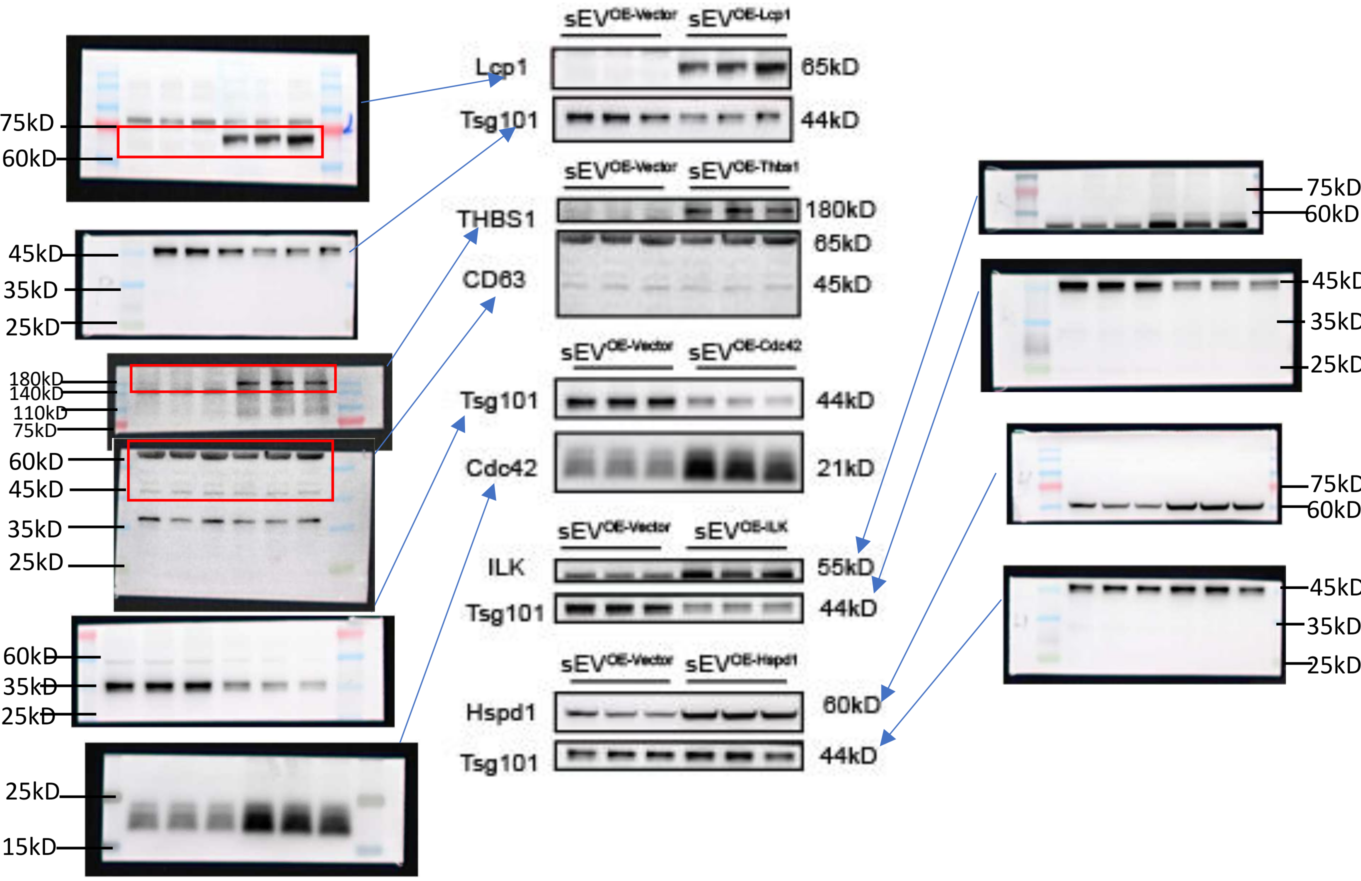

Figure 7.d

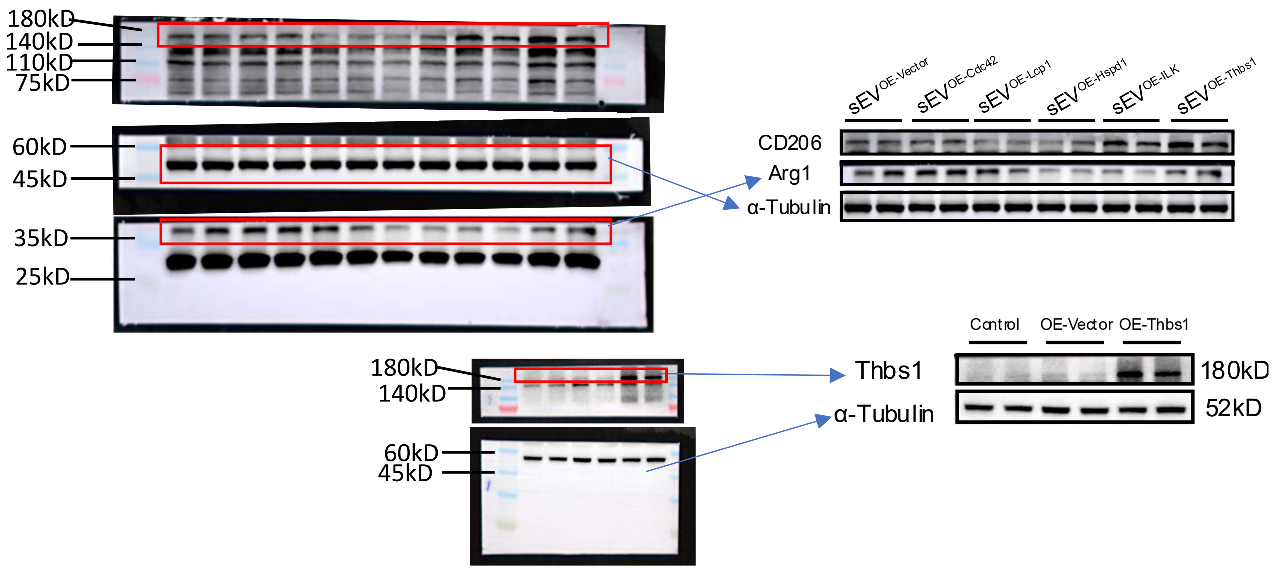

Figure 7.g

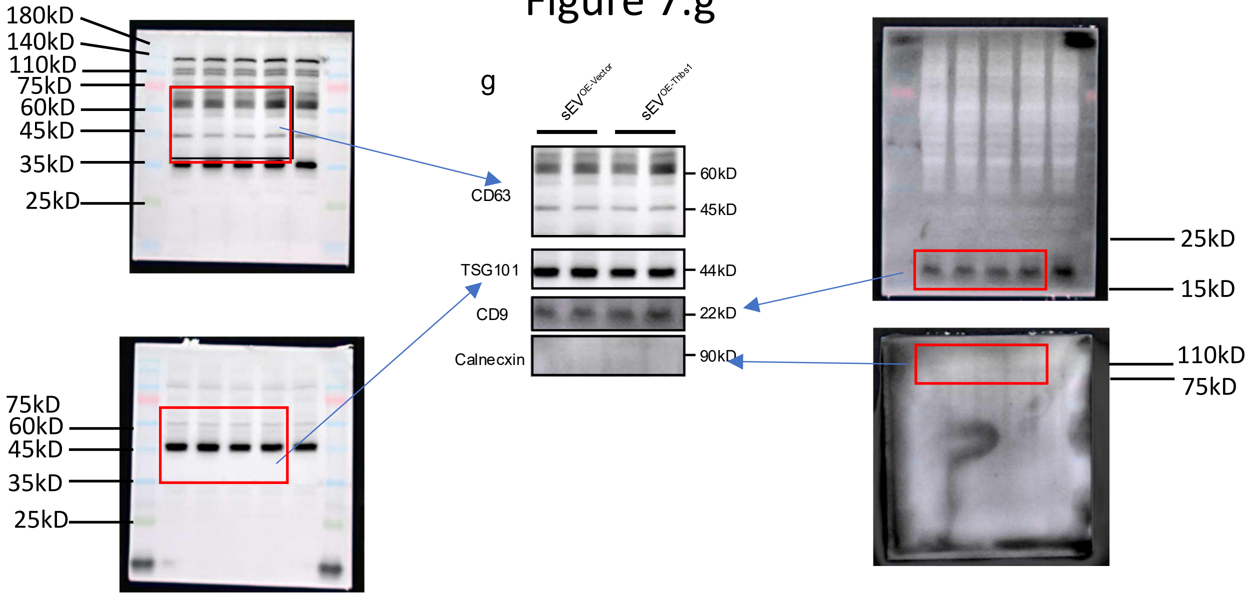

Figure 8.k &l

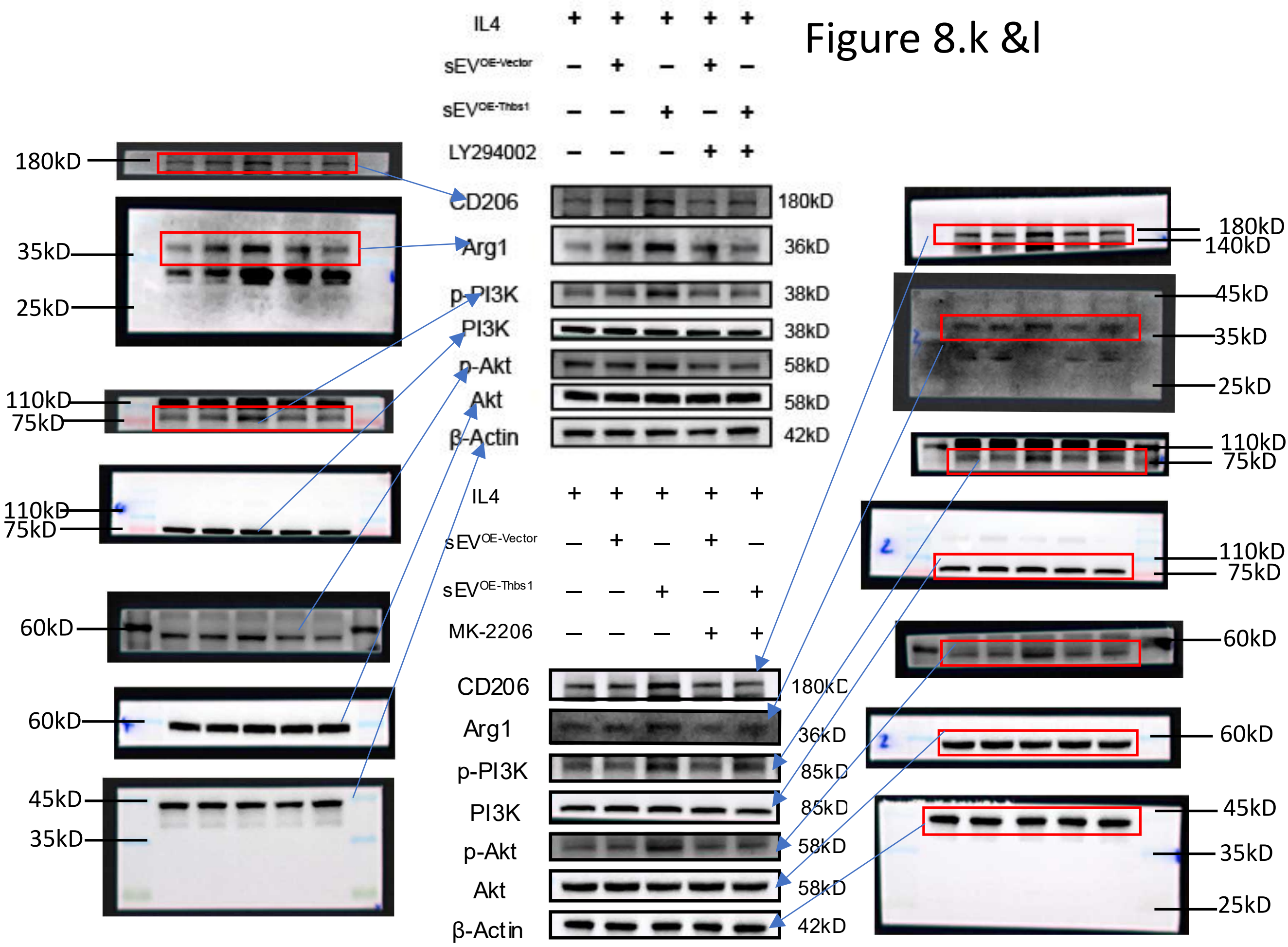

Figure 9.m

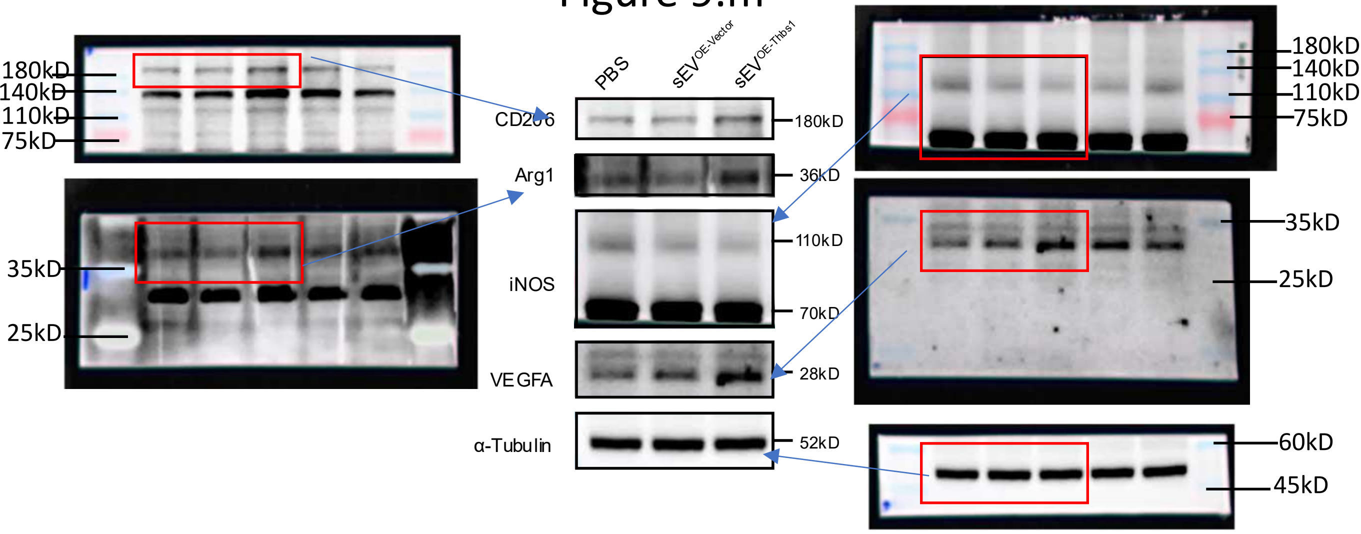

Supplement: Supplementary file 2 — Supporting File 2: advs73673‐sup‐0002‐Data.zip. [file ADVS-13-e17257-s002.zip › advs73673-sup-0002-Data/Western blot_raw data.pdf]
